# Pyridine-Ureas as Potential Anticancer Agents: Synthesis and In Vitro Biological Evaluation

**DOI:** 10.3390/molecules23061459

**Published:** 2018-06-15

**Authors:** Mohamed El-Naggar, Hadia Almahli, Hany S. Ibrahim, Wagdy M. Eldehna, Hatem A. Abdel-Aziz

**Affiliations:** 1 Chemistry Department, Faculty of Sciences, University of Sharjah, Sharjah 27272, UAE; m5elnaggar@yahoo.com; 2 Department of Chemistry, Chemistry Research Laboratory, University of Oxford, 12 Mansfield Road, Oxford OX1 3TA, UK; hadia.almahli@yahoo.com; 3 Department of Pharmaceutical Chemistry, Faculty of Pharmacy, Egyptian Russian University, Badr City, Cairo 11829, Egypt; hany.s.ibrahim@gmail.com; 4 Department of Pharmaceutical Chemistry, Faculty of Pharmacy, Kafrelsheikh University, Kafrelsheikh 33516, Egypt; 5 Department of Applied Organic Chemistry, National Research Center, Dokki, Cairo 12622, Egypt; hatem_741@yahoo.com

**Keywords:** pyridine-urea, breast cancer, anticancer, VEGFR-2, synthesis, ADME

## Abstract

In our endeavor towards the development of effective anticancer agents, a novel series of pyridine-ureas **8a**–**n** were synthesized. All the newly prepared derivatives were evaluated in vitro for their growth inhibitory activity towards the proliferation of breast cancer MCF-7 cell line. Compounds **8e** and **8n** were found to be the most active congeners against MCF-7 cells (IC_50_ = 0.22 and 1.88 µM after 48 h treatment; 0.11 and 0.80 µM after 72 h treatment, respectively) with increased activity compared to the reference drug doxorubicin (IC_50_ = 1.93 µM). Moreover, eight selected pyridines **8b**, **8d**, **8e**, **8i**, **8j** and **8l**–**n** were evaluated for their in vitro anticancer activity according to the US-NCI protocol. Pyridines **8b** and **8e** proved to be the most effective anticancer agents in the NCI assay with mean inhibition = 43 and 49%, respectively. Both **8b** and **8e** exhibited anti-proliferative activity against all tested cancer cell lines from all subpanels growth inhibition (GI for **8b**; 12–78%, GI for **8e**; 15–91%). Pyridines **8b** and **8e** were screened in vitro for their inhibitory activity against VEGFR-2. Both compounds inhibited VEGFR-2 at micromolar IC_50_ values 5.0 ± 1.91 and 3.93 ± 0.73 µM, respectively. The most active pyridines were filtered according to the Lipinski and Veber rules and all of them passed these filters. Finally, several ADME descriptors were predicted for the active pyridines through a theoretical kinetic study.

## 1. Introduction

Cancer represents one of the most important health problems worldwide because it is deemed to be the second major cause of mortality throughout the world after cardiovascular diseases. The high mortality rate that occurs in cancer patients is due to the late diagnosis, and consequently the delayed initiation of the medical treatment of the disease. Although there are a large number of chemotherapeutic drugs available, the medical need is still largely unmet. According to the latest GLOBOCAN statistics, about 14.1 million newly diagnosed cancer cases were recorded. Among the newly diagnosed cases in females, breast cancer ranked in the first position worldwide and was regarded as the main cause of deaths attributed to cancer [1,2]. Accordingly, there is a pressing necessity to pay much effort to modify drug leads from the point of view of drug design to afford new bioactive chemical entities that offer improvements over current therapies.

**Figure 1:**
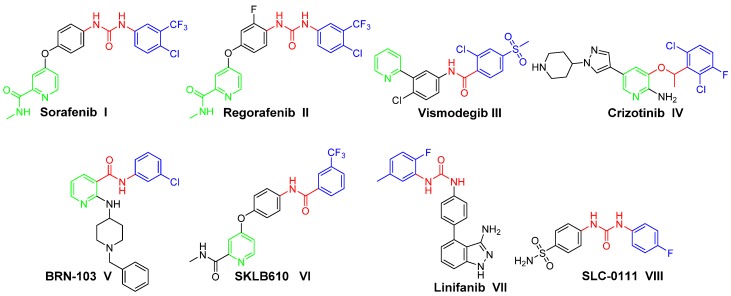
Structures of some pyridine-/urea-based approved anticancer drugs (**I**–**IV**), or in clinical trials (**V**–**XIII**), and the target conjugates (**4a**–**n** and **7a**–**d**).

In the current medical era, non-fused pyridines have emerged as a substantial class of heterocycles, which are endowed with diverse biological activities, chiefly anticancer activities [3,4,5]. Several pyridine-based small molecules have been approved as anticancer drugs, to name just a few: Sorafenib **I** (Nexavar^®^, Figure 1), Regorafenib **II** (Stivarga^®^, Figure 1), Vismodegib **III** (Erivedge^®^, Figure 1) and Crizotinib **IV** (Xalkori^®^, Figure 1) [6,7,8]. Subsequently, extensive efforts have been devoted to develop several pyridine-based derivatives as effective anticancer agents. BRN-103 **V** (Figure 1) is a nicotinamide derivative that suppresses the VEGF-induced phosphorylation of VEGFR-2 and the activation of AKT and eNOS. Also, it inhibits VEGF-induced migration, proliferation and capillary-like tube formation of HUVECs [9]. SKLB610 **VI** (Figure 1) is a multi-targeted kinase inhibitor activity towards VEGFR-2 and FGFR-2. SKLB610 has anti-proliferative effects, especially on human colorectal cancer cell line HCT-116 and human NSCLC cell line A549, with significant activity on tumor xenografts in nude mice without evident toxicity [10]. 

On the other hand, urea derivatives represent one of the most useful classes of anticancer agents, with a wide range of activities towards various tumors [11]. Urea functionality is the main pharmacophoric feature in several anticancer drugs such as Sorafenib **I** and Regorafenib **II**. Linifanib **VII** (Figure 1), an ureido indazole derivative, has selective inhibitory activity towards VEGFR and PDGFRs. Linifanib is in phase II clinical trials for patients with locally advanced or metastatic non-small cell lung cancer [12]. SLC-0111 **VIII** (Figure 1), an ureido benzenesulfonamide derivative, is currently in phase I/II clinical trials as a potential anticancer drug. SLC-0111 exhibited selectivity towards inhibition of the transmembrane isoforms human carbonic anhydrase (hCA) IX/XII (over the cytosolic isoforms hCA I/II). Also, SLC-0111 has the ability to block human breast cancer invasion, delay tumor growth and diminish the cancer stem cell population in vivo [13,14,15,16]. 

**Figure 2:**
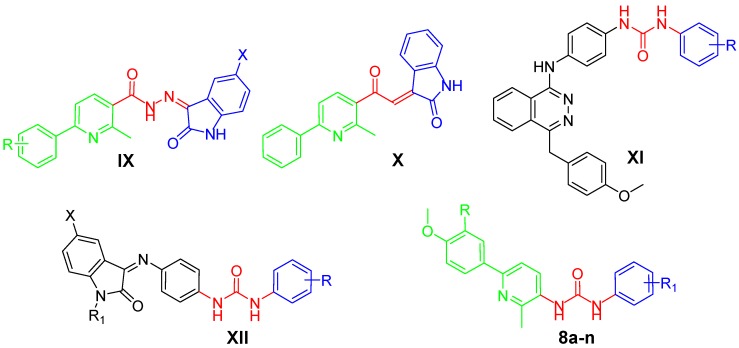
Structures of some pyridine-/urea-based anticancer agents reported by our research group (**IX**–**XII**), and the target pyridine-ureas **8a**–**n**.

Recently, our research team has paid much attention to developing diverse novel small molecules, based on the pyridine core (structures **IX** and **X**, Figure 2) or incorporating the ureido functionality (structures **XI** and **XII**, Figure 2), as potent anticancer agents. These molecules displayed promising anticancer activities through different molecular and enzymatic targets, such as cytotoxic action [17,18], VEGFR-2 inhibition [19,20] or tumor-associated hCA isoform IX and XII inhibition [21].

Taking the above into account and as a continuation of our endeavor towards developing novel anticancer agents [22,23,24,25], it was thought worthwhile to extend our examinations to probe certain pyridine-urea derivatives displaying potent anticancer activity. In this study, a new series of pyridine-ureas **8a**–**n** (Figure 2) was synthesized as potential anticancer agents. The latter synthesized pyridine-ureas were evaluated for their growth inhibitory activity towards the proliferation of breast cancer MCF-7 cell line. Moreover, eight selected pyridines **8b**, **8d**, **8e**, **8i**, **8j** and **8l**–**n** were evaluated for their in vitro anticancer activity according to US-NCI protocol over 58 cancer cell lines. Moreover, the most active pyridines were screened in vitro for their inhibitory activity against VEGFR-2. Finally, several ADME descriptors were predicted for the active pyridines through a theoretical kinetic study.

## 2. Results

### 2.1. Chemistry

**Scheme 1:**
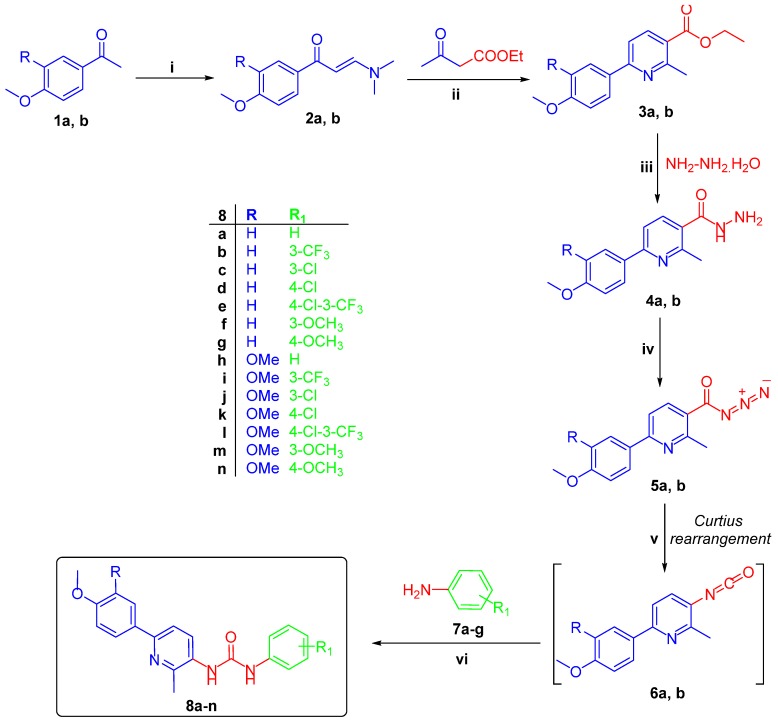
Synthesis of target pyridine-ureas **8a**–**n**; *Reagents and conditions*: (**i**) DMF-DMA, xylene, reflux 7hrs; (**ii**) NH_4_OAc, AcOH, reflux 4 h; (**iii**) Methanol, NH_2_NH_2_·H_2_O, reflux 2 h; (**iv**) NaNO_2_, AcOH, stirring 2 h; (**v**) Xylene, reflux 1 h; (**vi**) Xylene, reflux 3 h.

The target pyridine-ureas **8a**–**n** were synthesized adopting the chemical pathway outlined in Scheme 1. In a one-pot three-component heterocyclocondensation process, ethyl 6-(4-methoxyphenyl)-2-methylnicotinate **3a** and 6-(3,4-dimethoxyphenyl)-2-methylnicotinate **3b** were prepared through the reaction of enaminones **2a**,**b** with ethyl acetoacetate and ammonium acetate in glacial acetic acid under reflux temperature. Hydrazinolysis of esters **3a**,**b** were achieved through refluxing with hydrazine hydrate in methanol to afford hydrazides **4a**,**b** in 80% and 86% yield, respectively. Stirring of hydrazides **4a**,**b** with sodium nitrite in glacial acetic acid in an ice bath furnished nicotinoyl azide **5a**,**b**. Finally, preparation of pyridine-ureas **8a**–**n** were accomplished via addition of anilines **7a**–**g** to a pre-heated solution of nicotinoyl azide **5a**,**b** in xylene, with good yields; 72–83% (Scheme 1).

Postulated structures of the newly synthesized pyridine-ureas **8a**–**n** were in full agreement with their spectral and elemental analyses data (Supplementary Materials).

### 2.2. Biological Evaluation

#### 2.2.1. In Vitro Anti-Proliferative Activity

The in vitro anti-proliferative activity of the newly prepared pyridine-ureas **8a**–**n** was examined against breast cancer MCF-7 cell line. The assay was carried out, as triplicates, utilizing the 3-(4,5-dimethylthiazol-2-yl)-2,5-diphenyltetrazolium bromide (MTT) colorimetric assay as described by T. Mosmann [26]. Doxorubicin and Sorafenib were included in this assay as a reference drug. The results were expressed as median growth inhibitory concentration (IC_50_) values that represent the compound concentrations required to afford a 50% inhibition of cell growth after 48 and 72 h of incubation, compared to the untreated controls (Table 1).

From the obtained results, it was obvious that most of the prepared pyridine-ureas **8a**–**n** exhibited excellent to moderate anti-proliferative activity against MCF-7 breast cancer. Regarding the activity after 48 h treatment, compound **8e** (IC_50_ = 0.22 μM) was found to be the most potent derivative as it was 8.7 times more active than Doxorubicin (IC_50_ = 1.93 μM) and 20 times more than Sorafenib (IC_50_ = 4.50 μM). Also, compound **8n** displayed excellent anti-proliferative activity against MCF-7 cells (IC_50_ = 1.88 μM) which is comparable to Doxorubicin and better than Sorafenib. Furthermore, pyridines **8a**–**d**, **8g**, **8i**, **8k** and **8l** displayed potent activity against MCF-7 cells with IC_50_ range 3.03–7.03 µM. Moreover, pyridines **8j** and **8m** were moderately active with IC_50_ values of 10.09 and 23.02 μM, respectively. Unfortunately, compounds **8f** and **8h** did not display significant activity towards MCF-7 cells (IC_50_ > 50 μM). 

On the other hand, investigation of the anti-proliferative activity towards MCF-7 cells at 72 h of treatment elucidated that compounds **8g**, **8j** and **8l** possessed higher activity at 72 h compared to 48 h. The drop in activity after longer incubation time, 72 h, could be explained by the rapid metabolism for such compounds or that the tested MCF-7 cells were resistant to compounds **8g**, **8j** and **8l** upon 72 h treatment. With an exception of compound **8m** (IC_50_ = 13.1 μM) and compounds **8f** and **8h** (IC_50_ > 50 μM), all the tested pyridines possessed potent activity against MCF-7 cells with IC_50_ range 0.11–5.14 µM, upon 72 h treatment. 

#### 2.2.2. NCI, USA Cytotoxicity Assay towards 60 Cancer Cell Lines

The structures of all the newly prepared pyridine-ureas **8a**–**n** were submitted to the National Cancer Institute (NCI) Developmental Therapeutic Program. Eight compounds **8b**, **8d**, **8e**, **8i**, **8j** and **8l**–**n** were selected to be examined for their in vitro growth inhibitory activity. The anticancer assays were carried out according to the protocol of the Drug Evaluation Branch, NCI, Bethesda [27,28,29]. The selected pyridine-ureas were tested at one dose primary anticancer assay against a panel of approximately 58 cancer lines (concentration 10^−5^ M). The tested human cancer cell lines emerged from nine different cancer subtypes: leukemia, colon, melanoma, ovarian, lung, CNS, renal, breast and prostate cancers. The sulforhodamine B (SRB) protein assay was adopted to estimate the cell viability and growth [30]. The results were reported as mean-graph of the percentage growth of the treated cells, and presented as percentage growth inhibition (GI%) caused by the test compounds (Table 2). The gained data disclosed that most tested pyridines possessed distinctive patterns of selectivity and sensitivity towards the different NCI cancer cell panels (Supplementary Material).

**Figure 3:**
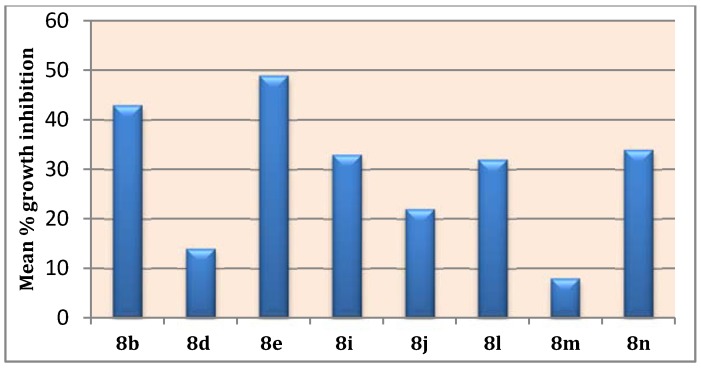
Mean % growth inhibitions of the tested pyridines over NCI 58 cell line panel.

The obtained GI% values (Table 2), showed that pyridine **8e** was found to be the most active member in this study with mean inhibition = 49%, Figure 3. Compound **8e** exhibited anti-proliferative activity against all tested cancer cell lines from all subpanels (GI; 15–91%) with a potent growth inhibitory effect over leukemia K-562, MOLT-4 and RPMI-8226, non-small cell lung cancer NCI-H522, colon cancer HCT-116, prostate cancer PC-3 and breast cancer T-47D with inhibition % 84, 76, 91, 76, 77, 86 and 79, respectively. Moreover, compound **8b** emerged as the second most active analogue (mean inhibition range =43%, Figure 3) with good activity against all cell lines (GI; 12–78%) except the ovarian cancer IGROV1 cell line. Additionally, pyridines **8i**, **8j**, **6l**, and **8n** showed moderate growth inhibitory activities (mean inhibition range: 22–34%, Figure 3) with a distinctive pattern of selectivity and sensitivity against different NCI cancer cell lines. Nevertheless, pyridines **8d** and **8m** possessed fair and selective growth inhibitory activities (mean inhibition range = 14 and 8%, respectively, Figure 3) towards sporadic cell lines. Notably, compound **8m** did not show any significant activity against the cell lines of CNS and ovarian subpanels.

**Figure 4:**
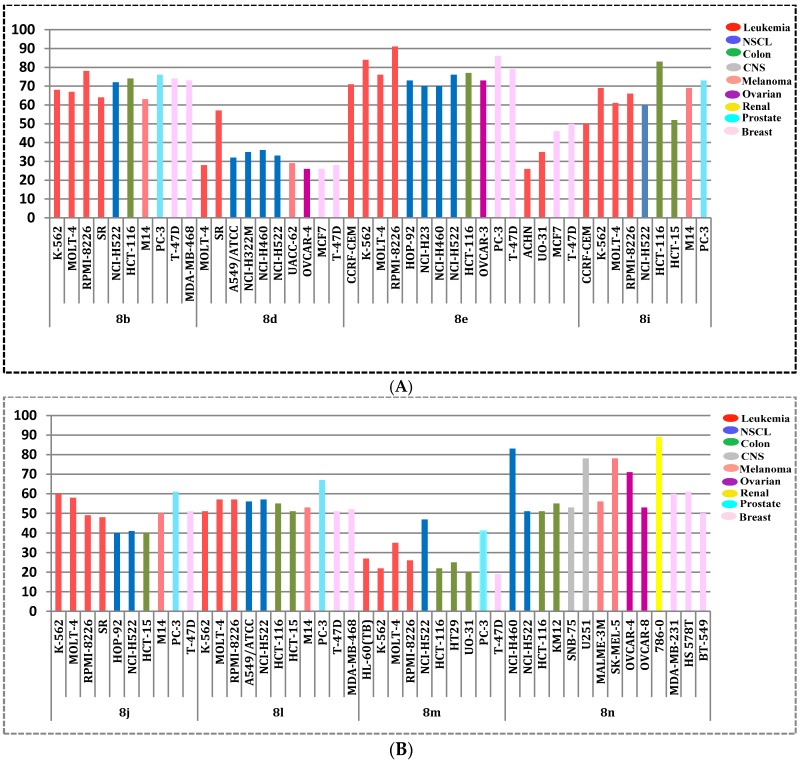
(**A**) The most sensitive cell lines towards the target pyridines **8b**, **8d**, **8e** and **8i** according to the GI%; (**B**) the most sensitive cell lines towards the target pyridines **8j**, **8l**, **8m** and **8n** according to the GI%.

Interestingly, all cell lines of the leukemia subpanel were sensitive to all the tested pyridines **8b**, **8d**, **8e**, **8i**, **8j** and **8l**–**n** (GI; 10–91%), except for compound **8n** towards CCRF-CEM cells. Besides, non-small cell lung cancer (A549/ATCC, HOP-92 and NCI-H522), colon cancer (HCT-116 and HT29), melanoma (M14 and SK-MEL-5), renal cancer (A498 and UO-31), prostate cancer (PC-3) and breast cancer (T-47D and MCF7) were susceptible cell lines to all the screened compounds. The most sensitive cell lines towards the target pyridines were displayed in Figure 4A,B.

#### 2.2.3. Cytotoxic Activity against Non-Tumorigenic Human WI-38 Cells

The cytotoxic activity of compounds **8b** and **8e** were evaluated against non-tumorigenic human lung fibroblast WI-38 cell line utilizing the MTT colorimetric assay developed by T. Mosmann [26] (Table 3). Interestingly, both the tested compounds **8b** and **8e** displayed non-significant weak cytotoxic activity towards WI-38 cell line with IC_50_ values of 91.5 ± 4.53 and 83.14 ± 5.21 μM, respectively.

#### 2.2.4. VEGFR-2 Inhibitory Assay

In an attempt to gain further mechanistic insights for the promising anti-proliferative activity of the prepared pyridine-ureas, compounds **8b** and **8e**, with significant anti-proliferative activity, were evaluated in vitro for their VEGFR-2 inhibitory activity. Sorafenib, a pyridine urea-based and well-known FDA-approved VEGFR-2 inhibitor, was used as the reference drug. The results are reported as median inhibition concentrations (IC_50_) which are determined as triplicate determinations from the standard curve and listed in Table 4. 

Results revealed that the tested pyridines **8b** and **8e** exhibited modest VEGFR-2 inhibitory activity with IC_50_ values of 5.0 ± 1.91 and 3.93 ± 0.73 μM, respectively, with respect to the reference drug Sorafenib (IC_50_ = 0.09 ± 0.01 μM). 

### 2.3. Physicochemical Properties and ADME Profiling

With a view to investigate drug-like physicochemical and pharmacokinetics properties of the target pyridine-ureas, several ADME descriptors for the active pyridines (**8a**, **8b**, **8e**, **8g**, **8i**, **8l** and **8n**) were estimated, in addition to the assessment of the criteria of both the Lipinski rule of five [31] and Veber rule [32]. A computer-aided theoretical kinetic study was performed via Discovery Studio 2.5 software (Accelrys, San Diego, CA, USA) to estimate different ADME descriptors for the most active anti-proliferative pyridines **8a**, **8b**, **8e**, **8g**, **8i**, **8l** and **8n**, Table 5. 

All the tested pyridines exhibited low aqueous solubility levels along with a good level of human intestinal absorption. Regarding blood-brain barrier penetration, the tested pyridines were discovered to possess certain penetrability to the blood-brain barrier. On the other hand, the investigated pyridines passed the filter of both the Lipinski rule of five and Veber rule, which implies that the synthesized pyridines have the privilege to possess good oral bioavailability, Table 6.

## 3. Experimental

### 3.1. Chemistry

Melting points were measured with a Stuart melting point apparatus (Bibby Scientific Limited, Staffordshire, UK) and were uncorrected. Infrared measurements (neat, thin film) were carried out using Schimadzu FT-IR 8400S spectrophotometer (Shimadzu, Kyoto, Japan). ^1^H-NMR and ^13^C-NMR experiments were carried out using Bruker AVF-400 (400/100 MHz) and AVC-500 (500/125 MHz) (Bruker, Karlsruhe, Germany), respectively. Chemical shifts (δ_H_) are reported relative to TMS as the internal standard. All coupling constant (*J*) values are given in hertz. Chemical shifts (δ_C_) were reported as follows: s, singlet; d, doublet; m, multiplet. High-resolution mass spectra (EI and ESI) were recorded using a Bruker MicroTOF spectrometer (Bruker Daltonics, Bremen, Germany) by the internal service at the University of Oxford. Analytical thin layer chromatography (TLC) (Merck KGaA, Darmstadt, Germany) on silica gel plates containing UV indicator was employed routinely to follow the course of reactions and to check the purity of products. All reagents and solvents were purified and dried by standard techniques. Compounds **2a**,**b**, **3a**,**b**, and **4a**,**b** [17,33] were previously prepared.

#### 3.1.1. Synthesis of 6-(4-Methoxyphenyl/3,4-dimethoxyphenyl)-2-methylnicotinohydrazide **4a**,**b**

To a suspension of esters **3a**,**b** (10 mmol) in methyl alcohol, 99% hydrazine hydrate (3 mL) was added. The reaction mixture was heated under reflux for 2 h. The precipitate formed was collected by filtration while hot, washed with water, dried and recrystallized from ethanol to produce hydrazides **4a**,**b** in 80% and 86% yield, respectively. The physical properties and spectral data of **4a**,**b** were identical with those reported [28].

#### 3.1.2. General Procedure for the Preparation of Target Pyridine-Ureas **8a**–**n**

In an ice bath, a mixture of hydrazides **4a**,**b** (5 mmol) and sodium nitrite (0.5 g, 7 mmol) was stirred in glacial acetic acid for 1 h, then stirring was continued at room temperature for another 1 h. The obtained solid was collected by filtration, washed with cold water and air-dried to furnish 6-(4-methoxyphenyl/3,4-dimethoxyphenyl)-2-methylnicotinoyl azide **5a**,**b**, which used in the next reaction without further purification. Then, the appropriate nicotinoyl azide **5a**,**b** was heated under reflux in dry xylene for 1 h before addition of anilines **7a**–**g**. The reaction mixture was refluxed for 3 h then allowed to cool to room temperature. The formed precipitate was filtered off, washed with cold acetone, dried and recrystallized from dioxane to furnish the target pyridines **8a**–**n**.

*1-(6-(4-Methoxyphenyl)-2-methylpyridin-3-yl)-3-phenylurea* (**8a**). White crystals (yield 72%), m.p. 205–207 °C; IR (KBr, *ν* cm^−1^) 3387 (NH), 1653 (C=O); ^1^H-NMR (CDCl_3_-*d*) δ ppm: 2.51 (s, 3H, -CH_3_), 3.86 (s, 3H, -OCH_3_), 6.29 (s, 1H, NH, D_2_O exchangeable); 6.40 (s, 1H, NH, D_2_O exchangeable), 6.98 (d, 2H, *J* = 8.8 Hz, Ar-H), 7.16 (m, 1H, Ar-H), 7.38 (m, 4H, Ar-H), 7.55 (d, 1H, *J* = 8.4 Hz, Ar-H), 7.93 (d, 2H, *J* = 8.8 Hz, Ar-H), 8.05 (d, 1H, *J* = 8.4 Hz, Ar-H); ^13^C-NMR (DMSO-*d*_6_) δ ppm: 21.38 (CH_3_), 55.18 (OCH_3_), 113.99, 116.96, 118.10, 121.96, 127.24, 128.25, 128.88, 131.17, 132.13, 139.59, 147.36, 149.00, 152.62 (C=O), 159.51 (=C-O-CH_3_); HRMS (ESI) *m*/*z* calcd for [M + H]^+^ (C_20_H_20_N_3_O_2_): 334.15500, found: 334.15499; Anal. Calcd. for C_20_H_20_N_3_O_2_ (333.39): C, 72.05; H, 5.74; N, 12.60; found C, 72.04; H, 5.71; N, 12.61.

*1-(6-(4-Methoxyphenyl)-2-methylpyridin-3-yl)-3-(3-(trifluoromethyl)phenyl) Urea* (**8b**). White crystals (yield 75%), m.p. 189–191 °C; IR (KBr, *ν* cm^−1^) 3405 (NH), 1716 (C=O); ^1^H-NMR (CDCl_3_-*d*) δ ppm: 2.60 (s, 3H, -CH_3_), 3.87 (s, 3H, -OCH_3_), 6.28 (s, 1H, NH, D_2_O exchangeable); 6.62 (s, 1H, NH, D_2_O exchangeable), 6.99 (d, 2H, *J* = 8.8 Hz, Ar-H), 7.35 (d, 1H, *J* = 7.7 Hz, Ar-H), 7.44 (t, 1H, *J* = 8 Hz, Ar-H), 7.58 (d, 1H, *J* = 8.4 Hz, Ar-H), 7.61 (d, 1H, *J* = 8 Hz, Ar-H), 7.68 (s, 1H, Ar-H), 7.89–7.99 (m, 3H, Ar-H); ^13^C-NMR (DMSO-*d*_6_) δ ppm: 21.34 (CH_3_), 55.18 (OCH_3_), 114.00, 116.97, 118.20, 121.70, 123.12, 125.28, 127.32, 128.97, 129.45, 129.70, 130.03, 131.09, 131.70, 140.49, 148.03, 149.52, 152.67 (C=O), 159.59 (=C-O-CH_3_); HRMS (ESI) *m*/*z* calcd for [M + H]^+^ (C_21_H_19_N_3_O_2_F_3_): 402.14239, found: 302.14220; Anal. Calcd. for C_21_H_19_N_3_O_2_F_3_ (401.39): C, 62.84; H, 4.52; N, 10.47; found C, 62.81; H, 4.52; N, 10.44.

*1-(3-Chlorophenyl)-3-(6-(4-methoxyphenyl)-2-methylpyridin-3-yl) Urea* (**8c**). White crystals (yield 81%), m.p. 212–214 °C; IR (KBr, *ν* cm^−1^) 3395 (NH), 1733 (C=O); ^1^H-NMR (MeOD-*d_4_*) δ ppm: 2.50 (s, 3H, CH_3_), 3.78 (s, 3H, -OCH_3_), 6.28 (s, 1H, NH, D_2_O exchangeable); 6.62 (s, 1H, NH, D_2_O exchangeable), 6.89–6.99 (m, 3H_,_ Ar-H), 7.15–7.25 (m, 2H, Ar-H), 7.52 (dd, 2H, *J* = 2.1 Hz, *J* = 8.4 Hz, Ar-H), 7.80 (d, 2H, *J* = 8.8 Hz, Ar-H), 8.09 (d, 1H, *J* = 8.4 Hz, Ar-H); ^13^C-NMR (DMSO-*d*_6_) δ ppm: 21.35 (CH_3_), 55.18 (OCH_3_), 114.00, 116.55, 116.97, 117.47, 121.57, 127.30, 128.76, 130.49, 131.10, 131.77, 133.26, 141.15, 147.85, 149.40, 152.52 (C=O), 159.57 (=C-O-CH_3_); HRMS (ESI) *m*/*z* calcd for [M + H]^+^ (C_20_H_19_N_3_O_2_Cl): 368.11603, found: 368.11605; Anal. Calcd. for C_20_H_19_N_3_O_2_Cl (367.83): C, 65.31; H, 4.93; N, 11.42; found C, 65.34; H, 4.90; N, 11.41.

*1-(4-Chlorophenyl)-3-(6-(4-methoxyphenyl)-2-methylpyridin-3-yl) Urea* (**8d**). White crystals (yield 83%), m.p. 231–232 °C; IR (KBr, *ν* cm^−1^) 3387 (NH), 1733 (C=O); ^1^H-NMR (CDCl_3_-*d*) δ ppm: 2.57 (s, 3H, -CH_3_), 3.87 (s, 3H, -OCH_3_), 6.18 (s, 1H, NH, D_2_O exchangeable); 6.37 (s, 1H, NH, D_2_O exchangeable), 6.99 (d, 2H, *J* = 8.8 Hz, Ar-H), 7.27–7.38 (m, 3H, Ar-H), 7.52–7.60 (m, 2H, Ar-H), 7.91–8.00 (m, 3H, Ar-H); ^13^C-NMR (DMSO-*d*_6_) δ ppm: 21.36 (CH_3_), 55.18 (OCH_3_), 113.99, 116.97, 119.63, 125.44, 127.27, 128.71, 131.12, 131.91, 138.61, 147.68, 149.24, 152.55 (C=O), 159.54 (=C-O-CH_3_); HRMS (ESI) *m*/*z* calcd for [M + H]^+^ (C_20_H_19_N_3_O_2_Cl): 368.11603, found: 368.11603; Anal. Calcd. for C_20_H_19_N_3_O_2_Cl (367.83): C, 65.31; H, 4.93; N, 11.42; found C, 65.30; H, 4.92; N, 11.40.

*1-(4-Chloro-3-(trifluoromethyl)phenyl)-3-(6-(4-methoxyphenyl)-2-methylpyridin-3-yl)urea* (**8e**). White crystals (yield 74%), m.p. 195–197 °C; IR (KBr, *ν* cm^−1^) 3394 (NH), 1733 (C=O); ^1^H-NMR (CDCl_3_-d) δ ppm: 2.61 (s, 3H, -CH_3_), 3.87 (s, 3H, -OCH_3_), 6.25 (s, 1H, NH, D_2_O exchangeable); 6.60 (s, 1H, NH, D_2_O exchangeable), 6.99 (d, 2H, *J* = 8.5 Hz, Ar-H), 7.44 (d, 1H, *J* = 8.7 Hz, Ar-H), 7.57–7.71 (m, 3H, Ar-H), 7.90–7.95 (m, 3H, Ar-H); ^13^C-NMR (DMSO-*d*_6_) δ ppm: 21.32 (CH_3_), 55.18 (OCH_3_), 114.01, 116.60, 116.98, 121.72, 122.37, 122.92, 123.86, 126.88, 127.35, 129.34, 131.05, 131.50, 132.10, 139.26, 148.37, 149.77, 152.59 (C=O), 159.62 (=C-O-CH_3_); HRMS (ESI) *m*/*z* calcd for [M + H]^+^ (C_21_H_18_N_3_O_2_ClF_3_): 436.10342, found: 436.10332; Anal. Calcd. for C_21_H_18_N_3_O_2_ClF_3_ (435.83): C, 57.87; H, 3.93; N, 9.64; found C, 57.82; H, 3.90; N, 9.61.

*1-(3-Methoxyphenyl)-3-(6-(4-methoxyphenyl)-2-methylpyridin-3-yl) Urea* (**8f**). White crystals (yield 83%), m.p. 227–230 °C; IR (KBr, *ν* cm^−1^) 3395 (NH), 1733 (C=O); ^1^H-NMR (CDCl_3_-*d*) δ ppm: 2.49 (s, 3H, CH_3_), 3.81 (s, 3H, -OCH_3_), 3.83 (s, 3H, -OCH_3_), 6.30 (s, 1H, NH, D_2_O exchangeable), 6.60 (s, 1H, NH, D_2_O exchangeable), 6.91–7.00 (m, 3H, Ar-H), 7.15-7.27 (m, 2H, Ar-H), 7.53 (dd, 2H, *J* = 2.1 Hz, *J* = 8.4 Hz, Ar-H), 7.80 (d, 2H, *J* = 8.8 Hz, Ar-H), 8.09 (d, 1H, *J* = 8.4 Hz, Ar-H); ^13^C-NMR (DMSO-*d*_6_) δ ppm: 21.32 (CH_3_), 55.18 (2 OCH_3_), 114.01, 116.60, 116.98, 121.72, 122.37, 122.92, 123.86, 126.88, 127.35, 129.34, 131.05, 131.50, 132.10, 139.26, 148.37, 149.77, 152.59 (C=O), 159.62 (=C-O-CH_3_); HRMS (ESI) *m*/*z* calcd for [M + H]^+^ (C_21_H_22_N_3_O_3_): 364.16557, found: 364.16565.

*1-(4-Methoxyphenyl)-3-(6-(4-methoxyphenyl)-2-methylpyridin-3-yl) Urea* (**8g**). White crystals (yield 80%), m.p. 241–242 °C; IR (KBr, *ν* cm^−1^) 3387 (NH), 1733 (C=O); ^1^H-NMR (CDCl_3_-*d*) δ ppm: 2.42 (s, 3H, -CH_3_), 3.83 (s, 3H, -OCH_3_), 3.85 (s, 3H, -OCH_3_), 6.22 (s, 1H, NH, D_2_O exchangeable), 6.60 (s, 1H, NH, D_2_O exchangeable), 6.91–7.00 (m, 4H, Ar-H), 7.53 (d, 2H, *J* = 8.4 Hz, Ar-H), 7.91 (d, 2H, *J* = 8.8 Hz, Ar-H), 8.13 (d, 2H, *J* = 8.4 Hz, Ar-H); ^13^C-NMR (DMSO-*d*_6_) δ ppm: 21.37 (CH_3_), 55.17 (2 OCH_3_), 159.47 (CO), 113.97, 114.07, 116.94, 119.87, 127.20, 128.02, 131.20, 132.33, 132.61, 147.15, 148.77, 152.77, 154.51 (C=O), 159.47 (=C-O-CH_3_); HRMS (ESI) *m*/*z* calcd for [M + H]^+^ (C_21_H_22_N_3_O_3_): 364.16557, found: 364.16597; Anal. Calcd. for C_21_H_22_N_3_O_3_ (363.42): C, 69.41; H, 5.82; N, 11.56; found C, 69.44; H, 5.80; N, 11.56.

*1-(6-(3,4-Dimethoxyphenyl)-2-methylpyridin-3-yl)-3-phenylurea* (**8h**). White crystals (yield 77%), m.p. 238–239 °C; IR (KBr, *ν* cm^−1^) 3398 (NH), 1733 (C=O); ^1^H-NMR (CDCl_3_-*d*) δ ppm: 2.42 (s, 3H, CH_3_), 3.83 (s, 3H, -OCH_3_), 3.85 (s, 3H, -OCH_3_), 6.32 (s, 1H, NH, D_2_O exchangeable); 6.44 (s, 1H, NH, D_2_O exchangeable), 6.94 (d, 1H, *J* = 8.6 Hz, Ar-H), 7.39–7.42 (m, 5H, Ar-H), 7.51–7.62 (m, 3H, Ar-H), 8.08 (d, 1H, *J* = 8.8 Hz, Ar-H); ^13^C-NMR (DMSO-*d*_6_) δ ppm: 21.42 (CH_3_), 55.52 (2 OCH_3_), 109.48, 111.73, 117.20, 118.12, 118.50, 121.96, 128.13, 128.88, 131.43, 132.20, 139.59, 147.25, 148.83, 149.00, 149.19, 152.63 (C=O); HRMS (ESI) *m*/*z* calcd for [M + H]^+^ (C_21_H_22_N_3_O_3_): 364.16557, found: 364.16528; Anal. Calcd. for C_21_H_22_N_3_O_3_ (363.42): C, 69.41; H, 5.82; N, 11.56; found C, 69.40; H, 5.80; N, 11.52.

*1-(6-(3,4-Dimethoxyphenyl)-2-methylpyridin-3-yl)-3-(3-(trifluoromethyl)phenyl) Urea* (**8i**). White crystals (yield 80%), m.p. 235–237 °C; IR (KBr, *ν* cm^−1^) 3396 (NH), 1733 (C=O); ^1^H-NMR (CDCl_3_-*d*) δ ppm: 2.55 (s, 3H, CH_3_), 3.93 (s, 3H, -OCH_3_), 3.98 (s, 3H, -OCH_3_), 6.56 (s, 1H, NH, D_2_O exchangeable), 6.93 (d, 1H, *J* = 8.6 Hz, Ar-H), 7.00 (s, 1H, Ar-H), 7.33 (d, 1H, *J* = 7.6 Hz, Ar-H), 7.40 (t, 1H, *J* = 7.6 Hz, Ar-H), 7.47 (dd, 1H, *J* = 2.0 Hz, *J* = 8.5 Hz, Ar-H), 7.52-7.62 (m, 2H, Ar-H), 7.65 (s, 1H, Ar-H), 7.97 (d, 1H, *J* = 8.6 Hz, Ar-H); ^13^C-NMR (DMSO-*d*_6_) δ ppm: 21.38 (CH_3_), 55.53 (2 OCH_3_), 109.53, 111.73, 114.02, 117.21, 118.60, 121.72, 123.12, 125.28, 128.82, 129.70, 130.04, 131.33, 131.77, 140.47, 147.89, 148.84, 149.28, 149.52, 152.65 (C=O); HRMS (ESI) *m*/*z* calcd for [M + H]^+^ (C_22_H_21_N_3_O_3_F_3_): 432.15295, found: 432.15283; Anal. Calcd. for C_22_H_21_N_3_O_3_F_3_ (431.42): C, 61.25; H, 4.67; N, 9.74; found C, 61.22; H, 4.65; N, 9.71.

*1-(3-Chlorophenyl)-3-(6-(3,4-dimethoxyphenyl)-2-methylpyridin-3-yl) Urea* (**8j**). White crystals (yield 76%), m.p. 249–250 °C; IR (KBr, *ν* cm^−1^) 3373 (NH), 1733 (C=O); ^1^H-NMR (CDCl_3_-*d*) δ ppm: 2.57 (s, 3H, -CH_3_), 3.94 (s, 3H, -OCH_3_), 3.99 (s, 3H, -OCH_3_), 6.37 (s, 1H, NH, D_2_O exchangeable), 6.62 (s, 1H, NH, D_2_O exchangeable), 6.94 (d, 1H, *J* = 8.4 Hz, Ar-H), 7.05–7.13 (m, 1H, Ar-H), 7.45–7.53 (m, 4H, Ar-H), 7.56 (d, 1H, *J* = 8.4 Hz, Ar-H), 7.63 (s, 1H, Ar-H), 7.99 (d, 1H, *J* = 8.4 Hz, Ar-H); ^13^C-NMR (DMSO-*d*_6_) δ ppm: 21.41 (CH_3_), 55.54 (2 OCH_3_), 109.52, 111.74, 116.57, 117.22, 117.48, 118.58, 121.59, 128.63, 130.51, 131.35, 131.85, 133.27, 141.16, 147.73, 148.84, 149.26, 149.41, 152.53 (C=O); HRMS (ESI) *m*/*z* calcd for [M + H]^+^ (C_21_H_21_N_3_O_3_Cl): 398.12660, found: 398.12642; Anal. Calcd. for C_21_H_21_N_3_O_3_Cl (397.86): C, 63.40; H, 5.07; N, 10.56; found C, 63.41; H, 5.02; N, 10.54.

*1-(4-Chlorophenyl)-3-(6-(3,4-dimethoxyphenyl)-2-methylpyridin-3-yl) Urea* (**8k**). White crystals (yield 81%), m.p. 266–267 °C; IR (KBr, *ν* cm^−1^) 3388 (NH), 1733 (C=O); ^1^H-NMR (CDCl_3_-*d*) δ ppm: 2.57 (s, 3H, CH_3_), 3.94 (s, 3H, -OCH_3_), 4.00 (s, 3H, -OCH_3_), 6.22 (s, 1H, NH, D_2_O exchangeable), 6.41 (s, 1H, NH, D_2_O exchangeable), 6.95 (d, 1H, *J* = 8.5 Hz, Ar-H), 7.30–7.35 (m, 4H_,_ Ar-H), 7.51 (d, 1H, *J* = 8.8 Hz, Ar-H), 7.54–7.65 (m, 2H_,_ Ar-H), 7.99 (d, 1H, *J* = 8.5 Hz, Ar-H); ^13^C-NMR (DMSO-*d*_6_) δ ppm: 21.41 (CH_3_), 55.51 (2 OCH_3_), 109.50, 111.73, 117.20, 118.54, 119.64, 125.43, 128.46, 128.70, 131.38, 131.99, 138.63, 147.59, 148.83, 149.25, 152.56 (C=O); HRMS (ESI) *m*/*z* calcd for [M + H]^+^ (C_21_H_21_N_3_O_3_Cl): 398.12660, found: 398.12673; Anal. Calcd. for C_21_H_21_N_3_O_3_Cl (397.86): C, 63.40; H, 5.07; N, 10.56; found C, 63.40; H, 5.04; N, 10.52.

*1-(4-Chloro-4-(trifluoromethyl)cyclohexa-2,5-dien-1-yl)-3-(6-(3,4-dimethoxyphenyl)-2-methylpyridin-3-yl)urea* (**8l**). White crystals (yield 79%), m.p. 261–263 °C; IR (KBr, *ν* cm^−1^) 3393 (NH), 1733 (C=O); ^1^H-NMR (CDCl_3_-*d*) δ ppm: 2.51 (s, 3H, CH_3_), 3.92 (s, 3H, -OCH_3_), 3.96 (s, 3H, -OCH_3_), 6.22 (s, 1H, NH, D_2_O exchangeable), 6.74 (s, 1H, NH, D_2_O exchangeable), 6.91 (d, 1H, *J* = 8.6 Hz, Ar-H), 7.28 (s, 1H, Ar-H), 7.35 (d, 1H, *J* = 9.0 Hz, Ar-H), 7.50 (dd, 2H_,_ *J* = 2.5, *J* = 8.7 Hz, Ar-H), 7.58 (d, 1H, *J* = 2.0 Hz, Ar-H), 7.65 (d, 1H, *J* = 2.4 Hz, Ar-H), 7.91 (d, 1H, *J* = 8.6 Hz, Ar-H); ^13^C-NMR (DMSO-*d*_6_) δ ppm: 21.38 (CH_3_), 55.53 (2 OCH_3_), 109.56, 111.73, 117.22, 118.65, 121.74, 122.34, 122.93, 129.30, 131.32, 131.62, 132.10, 139.34, 148.33, 148.84, 149.32, 149.78, 152.65 (C=O); HRMS (ESI) *m*/*z* calcd for [M + H]^+^ (C_22_H_20_N_3_O_3_ClF_3_): 466.11398, found: 466.11398; Anal. Calcd. for C_22_H_20_N_3_O_3_ClF_3_ (465.86): C, 56.72; H, 4.11; N, 9.02; found C, 59.51; H, 4.61; N, 9.20

*1-(6-(3,4-Dimethoxyphenyl)-2-methylpyridin-3-yl)-3-(3-methoxyphenyl) Urea* (**8m**). White crystals (yield 80%), m.p. 243–245 °C; IR (KBr, *ν* cm^−1^) 3391 (NH), 1733 (C=O); ^1^H-NMR (CDCl_3_-*d*) δ ppm: 2.51 (s, 3H, -CH_3_), 3.80 (s, 3H, -OCH_3_), 3.92 (s, 3H, -OCH_3_), 3.98 (s, 3H, -OCH_3_), 6.22 (s, 1H, NH, D_2_O exchangeable), 6.56 (s, 1H, NH, D_2_O exchangeable), 6.66–6.75 (m, 2H, Ar-H), 6.83–6.96 (m, 2H_,_ Ar-H), 7.05 (t, 1H, *J* = 2.3 Hz, Ar-H), 7.20–7.29 (m, 2H_,_ Ar-H), 7.60 (d, 1H, *J* = 2.0 Hz, Ar-H), 8.06 (d, 1H, *J* = 8.5 Hz, Ar-H); ^13^C-NMR (DMSO-*d*_6_) δ ppm: 21.40 (CH_3_), 54.93 (OCH_3_), 55.53 (2 OCH_3_), 103.87, 107.38, 109.49, 110.42, 111.73, 117.19, 118.51, 128.19, 129.65, 131.42, 132.13, 140.81, 147.28, 148.83, 149.05, 149.20, 152.55 (C=O); HRMS (ESI) *m*/*z* calcd for [M + H]^+^ (C_22_H_24_N_3_O_4_): 394.17613, found: 394.17608; Anal. Calcd. for C_22_H_24_N_3_O_4_ (393.44): C, 67.16; H, 5.89; N, 10.68; found C, 67.12; H, 5.89; N, 10.67.

*1-(6-(3,4-Dimethoxyphenyl)-2-methylpyridin-3-yl)-3-(4-methoxyphenyl) Urea* (**8n**). White crystals (yield 80%), m.p. 253–254 °C; IR (KBr, *ν* cm^−1^) 3392 (NH), 1733 (C=O); ^1^H-NMR (CDCl_3_-*d*) δ ppm: 2.42 (s, 3H, -CH_3_), 3.83 (s, 3H, -OCH_3_), 3.92 (s, 3H, -OCH_3_), 3.98 (s, 3H, -OCH_3_), 6.27 (s, 1H, NH, D_2_O exchangeable), 6.56 (s, 1H, NH, D_2_O exchangeable), 6.94 (dd, 3H, *J* = 5.1 Hz, *J* = 8.8 Hz, Ar-H), 7.29 (d, 2H, *J* = 7.4 Hz, Ar-H), 7.47 (d, 1H, *J* = 7.9 Hz, Ar-H), 7.54 (d, 1H, *J* = 8.2 Hz, Ar-H), 7.59 (s, 1H, Ar-H) 8.15 (d, 1H, *J* = 8.0 Hz, Ar-H); ^13^C-NMR (DMSO-*d*_6_) δ ppm: 21.43 (CH_3_), 55.18 (OCH_3_), 55.53 (2 OCH_3_), 109.47, 111.73, 114.06, 117.18, 118.47, 119.89, 127.96, 131.48, 132.43, 132.66, 147.09, 148.79, 148.83, 149.16, 152.81 (C=O), 154.51 (=C-O-CH_3_); HRMS (ESI) *m*/*z* calcd for [M + H]^+^ (C_22_H_24_N_3_O_4_): 394.17613, found: 394.17598; Anal. Calcd. for C_22_H_24_N_3_O_4_ (393.44): C, 67.16; H, 5.89; N, 10.68; found C, 67.11; H, 5.87; N, 10.65.

### 3.2. Biological Evaluation

#### 3.2.1. In Vitro Anti-Proliferative Activity towards Breast Cancer MCF7 Cell Line

MCF-7 (human breast cancer cell line) and WI-38 (human lung fibroblast cell line), were obtained from American Type Culture Collection (Manassas, VA, USA). The cells were propagated in DMEM and supplemented with 10% heat-inactivated FBS (Hyclone), 10 μg/mL of insulin (Manufacturer, Sigma, St. Louis, MO, USA), and 1% penicillin-streptomycin. MTT assay was utilized to examine the in vitro anti-proliferative activity of the newly prepared ureas following the reported procedures [26,34,35]. The 50% inhibitory concentration (IC_50_) was estimated, after 48 and 72 h for MCF-7 cells, from graphic plots of the dose response curve for each conc. using Graphpad Prism software (GraphPad Software, Inc., San Diego, CA, USA). The data presented are the mean of at least three separate experiments. 

#### 3.2.2. In Vitro Cytotoxic Activity by NCI-USA

The anticancer assays were performed in accordance with the protocol of the Drug Evaluation Branch, NCI, Bethesda [27,28,29]. A 48 h drug exposure protocol was used and sulforhodamine B (SRB) protein assay [30] was applied to estimate the cell viability and growth, as reported earlier [36,37].

#### 3.2.3. Measurement of Inhibitory Activity against VEGFR-2

VEGFR-2 enzyme inhibition was measured for pyridines **8b** and **8e** using an anti-phosphotyrosine antibody with the Alpha Screen system (PerkinElmer, Waltham, MA, USA) according to the manufacturer’s protocol and referring to reported instructions [19]. The concentration of the test compound causing 50% inhibition (IC_50_) was calculated from the concentration–inhibition response curve (triplicate determinations) and the data were compared with sorafenib as standard VEGFR-2 inhibitor.

#### 3.2.4. Physicochemical Properties and ADME Profiling

Physicochemical properties and ADME profiling for the active pyridines (**8a**, **8b**, **8e**, **8g**, **8i**, **8l** and **8n**) were performed using Discovery Studio 2.5 (Accelrys, San Diego, CA, USA). The examined pyridines were drawn as a small library and prepared via prepare ligand protocol in order to find the suitable orientation in 3D. Then, the prepared library was filtered adopting the Lipinski and Veber rules protocols. ADME profiling was predicted for the designed library using ADME descriptors protocol.

## 4. Conclusions

In summary, herein we report the synthesis of a novel series of pyridine-ureas **8a**–**n**. All the prepared pyridines were evaluated for their anti-proliferative activity towards the breast cancer MCF-7cell line. Pyridines **8e** and **8n** were the most active members towards MCF-7 cells (IC_50_ = 0.22 and 1.88 µM after 48 h treatment; 0.11 and 0.80 µM after 72 h treatment, respectively). Furthermore, eight selected pyridines **8b**, **8d**, **8e**, **8i**, **8j** and **8l**–**n** were examined for their in vitro anticancer activity according to US-NCI protocol. Pyridines **8b** and **8e** emerged as the most effective anticancer agents in the NCI assay with mean inhibition = 43 and 49%, respectively. Both **8b** and **8e** exhibited anti-proliferative activity against all tested cancer cell lines from all subpanels (GI for **8b**; 12–78%, GI for **8e**; 15–91%). Pyridines **8b** and **8e** were screened in vitro for their inhibitory activity against VEGFR-2. Both compounds inhibited VEGFR-2 at micromolar IC_50_ values 5.0 ± 1.91 and 3.93 ± 0.73 µM, respectively. The most active pyridines were filtered according to the Lipinski and Veber rules and all of them passed these filters. Finally, several ADME descriptors were predicted for the active pyridines through a theoretical kinetic study. Further mechanistic studies are in progress in our laboratories and will be reported upon in the future

## Figures and Tables

**Table 1:**
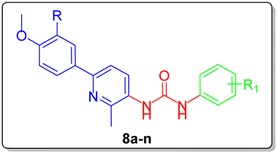
In vitro anti-proliferative activity of pyridine-ureas **8a**–**n** against MCF-7 breast cancer cell line.

Compound	R	R_1_	IC_50_ (µM) ^a^	
			48 h	72 h
**8a**	H	H	3.03 ± 0.22	2.83 ± 0.25
**8b**	H	3-CF_3_	5.66 ± 0.45	5.03 ± 0.29
**8c**	H	3-Cl	6.40 ± 0.41	3.52 ± 0.11
**8d**	H	4-Cl	7.10 ± 0.38	5.14 ± 0.46
**8e**	H	4-Cl-3-CF_3_	0.22 ± 0.02	0.11 ± 0.1
**8f**	H	3-OCH_3_	NA ^b^	NA ^b^
**8g**	H	4-OCH_3_	4.12 ± 0.27	27.24 ± 1.98
**8h**	OCH_3_	H	NA ^b^	NA ^b^
**8i**	OCH_3_	3-CF_3_	6.19 ± 0.54	5.80 ± 0.34
**8j**	OCH_3_	3-Cl	10.9 ± 1.03	26.2 ± 2.17
**8k**	OCH_3_	4-Cl	5.63 ± 0.36	3.45 ± 0.30
**8l**	OCH_3_	4-Cl-3-CF_3_	7.03 ± 0.61	21.43 ± 2.03
**8m**	OCH_3_	3-OCH_3_	23.02 ± 1.91	13.1 ± 1.12
**8n**	OCH_3_	4-OCH_3_	1.88 ± 0.12	0.80 ± 0.07
**Dox.**	-	-	1.93 ± 0.15	1.07 ± 0.07
**Sorafenib**	-	-	4.50 ± 0.30	1.71 ± 0.15

^a^ IC_50_ values are the mean ± S.D. of three separate experiments; ^b^ NA: Compounds having IC_50_ value > 50 µM.

**Table 2:** Percentage growth inhibition (GI%) of in vitro subpanel tumor cell lines at 10 μM concentration for compounds **8b**, **8d**, **8e**, **8i**, **8j** and **8l**–**n**.

Subpanel/Cell Line		Compound ^a^							
		8b (NSC 793907)	8d (NSC 793908)	8e (NSC 793925)	8i (NSC 793919)	8j (NSC 793924)	8l (NSC 793915)	8m (NSC 793929)	8n (NSC 793926)
**Leukemia**	CCRF-CEM	58	19	71	50	32	45	11	-
	HL-60(TB)	51	21	68	39	37	48	27	15
	K-562	68	16	84	69	60	51	22	27
	MOLT-4	67	28	76	61	58	57	35	15
	RPMI-8226	78	10	91	66	49	57	26	17
	SR	64	57	65	49	48	40	18	43
**Non-Small Cell Lung Cancer**	A549/ATCC	58	32	62	40	39	56	10	43
	EKVX	59	-	51	34	17	33	-	-
	HOP-62	33	19	38	-	23	23	-	44
	HOP-92	32	19	73	45	40	30	19	27
	NCI-H226	42	-	35	28	-	28	-	42
	NCI-H23	53	-	70	29	11	29	10	33
	NCI-H322M	36	35	29	-	15	13	-	14
	NCI-H460	52	36	70	39	17	48	-	83
	NCI-H522	72	33	76	60	41	57	47	51
**Colon Cancer**	COLO 205	16	-	20	12	-	-	-	-
	HCC-2998	19	-	31	16	12	-	-	17
	HCT-116	74	24	77	83	37	55	22	51
	HCT-15	60	15	63	52	40	51	-	-
	HT29	53	15	54	41	26	44	25	32
	KM12	55	-	63	34	22	30	-	55
	SW-620	19	23	33	-	-	10	-	36
**CNS Cancer**	SF-268	26	-	27	25	13	25	-	41
	SF-295	53	-	54	35	17	29	-	-
	SF-539	20	16	19	23	11	13	-	30
	SNB-19	33	-	43	22	-	22	-	29
	SNB-75	23	16	27	34	16	20	-	53
	U251	50	24	67	37	17	45	-	78
**Melanoma**	LOX IMVI	53	15	36	30	20	22	-	12
	MALME-3M	10	-	18	-	-	10	-	56
	M14	63	14	69	69	50	53	10	28
	MDA-MB-435	31	12	29	27	13	23	-	35
	SK-MEL-2	25	-	38	27	16	13	13	21
	SK-MEL-28	31	11	29	25	21	27	-	14
	SK-MEL-5	59	13	45	42	32	37	16	78
	UACC-257	39	22	43	19	29	44	-	45
	UACC-62	37	29	35	19	25	23	-	41
**Ovarian Cancer**	IGROV1	-	17	48	-	-	17	-	23
	OVCAR-3	59	-	73	31	10	43	-	-
	OVCAR-4	58	26	55	41	-	25	-	71
	OVCAR-5	17	-	11	-	12	15	-	-
	OVCAR-8	39	-	43	19	10	19	-	53
	NCI/ADR-RES	47	-	51	34	16	31	-	16
	SK-OV-3	18	18	32	-	17	16	-	26
**Renal Cancer**	786-0	23	10	44	31	29	25	-	89
	A498	48	21	43	39	31	43	15	29
	RXF 393	15	-	19	23	18	28	-	42
	SN12C	33	-	43	21	14	22	-	36
	TK-10	24	-	35	21	-	12	-	40
	UO-31	45	11	61	26	21	28	20	19
**Prostate**	PC-3	76	24	86	73	61	67	41	19
	DU-145	32	-	46	18	-	20	-	28
**Breast Cancer**	MCF7	59	26	64	38	25	42	10	35
	MDA-MB-231	32	-	15	31	13	12	-	60
	HS 578T	12	-	24	26	-	19	-	61
	BT-549	34	-	34	29	-	12	13	50
	T-47D	74	28	79	47	51	51	19	40
	MDA-MB-468	73	-	62	51	29	52	-	17

^a^ Only GI% higher than 10% are shown.

**Table 3:** Cytotoxic activity of compounds **8b** and **8e** against non-tumorigenic human lung fibroblast WI-38 cell line.

Compound	IC_50_ (μM) ^a^
	WI-38
**8b**	91.5 ± 4.53
**8e**	83.14 ± 5.21

**^a^** IC_50_ values are the mean ± S.D. of three separate experiments.

**Table 4:** IC_50_ values for the inhibitory activity of pyridine-urea **8e** and **8n** against VEGFR-2.

Compound	IC_50_ (μM) ^a^
	VEGFR-2
**8b**	5.00 ± 1.91
**8e**	3.93 ± 0.73
**Sorafenib**	0.09 ± 0.01

^a^ IC_50_ values are the mean ± S.D. of three separate experiments.

**Table 5:** ADME studies results for all the synthesized compounds.

Compound	ADMET Solubility ^1^	ADMET Solubility Level ^2^	ADMET Absorption Level ^3^	ADMET BBB ^4^	ADMET BBB Level ^4^
**8a**	−4.499	2	0	−0.045	2
**8b**	−5.93	2	0	0.247	1
**8e**	−6.698	1	0	0.452	1
**8g**	−4.554	2	0	−0.191	2
**8i**	−5.971	2	0	0.10	1
**8l**	−6.723	1	0	0.306	1
**8n**	−4.623	2	0	−0.337	2

^1^ The base 10 logarithm of the molar solubility as predicted by the regression model based by DS; ^2^ Categorical solubility level. (0 = extreme low, 1 = very low but possible, 2 = low, 3 = good, 4 = optimal); ^3^ Human intestinal absorption level. (0 = good, 1 = moderate, 2 = poor, 3 = very poor); ^4^ Blood brain barrier penetration. (0; ≥0.7 = very high penetrant, 1; 0 ≤ log BB < 0.7 = High penetrant, 2; −0.52 < LogBB < 0 = low, 3; logBB ≤ −0.52 = low, 4; NA = undefined).

**Table 6:** Lipinski rule of five parameters results for all the synthesized compounds.

Compound	H-Bond Donor ^1,^*	H-Bond Acceptor ^2,^*	Molecular Weight ^3^	ALogP ^4^	No. of Rotatable Bond ^5^	Polar Surface Area ^6^ (Å^2^)
**8a**	2	3	333.38	3.585	4	63.25
**8b**	2	3	401.38	4.527	5	63.25
**8e**	2	3	435.82	5.192	5	63.25
**8g**	2	4	363.41	3.569	5	72.48
**8i**	2	4	431.41	4.511	6	72.48
**8l**	2	4	465.85	5.175	6	72.48
**8n**	2	5	393.44	3.552	6	81.71

^1^ The upper limit of the number of hydrogen bond donors is 5; ^2^ the upper limit of the number of hydrogen bond acceptors is 10; ^3^ the upper limit of the molecular weight is 500; ^4^ the upper limit of the AlogP (the log value of octanol-water partition coefficient) is 500; ^5^ the upper limit of the number of rotatable bonds is 10; ^6^ the upper limit of the polar surface area is 140 Å^2^; * the upper limit of the sum of the hydrogen bond donors and acceptors is 12.

## Supplementary Materials

The following are available online. Spectral data and NCI One Dose Mean Graph.
