# Supplementary material for: Pyridine-Ureas as Potential Anticancer Agents: Synthesis and In Vitro Biological Evaluation
Source: Molecules. 2018 Jun 15;23(6):1459. doi: 10.3390/molecules23061459 (PMC6100082; doi:10.3390/molecules23061459)

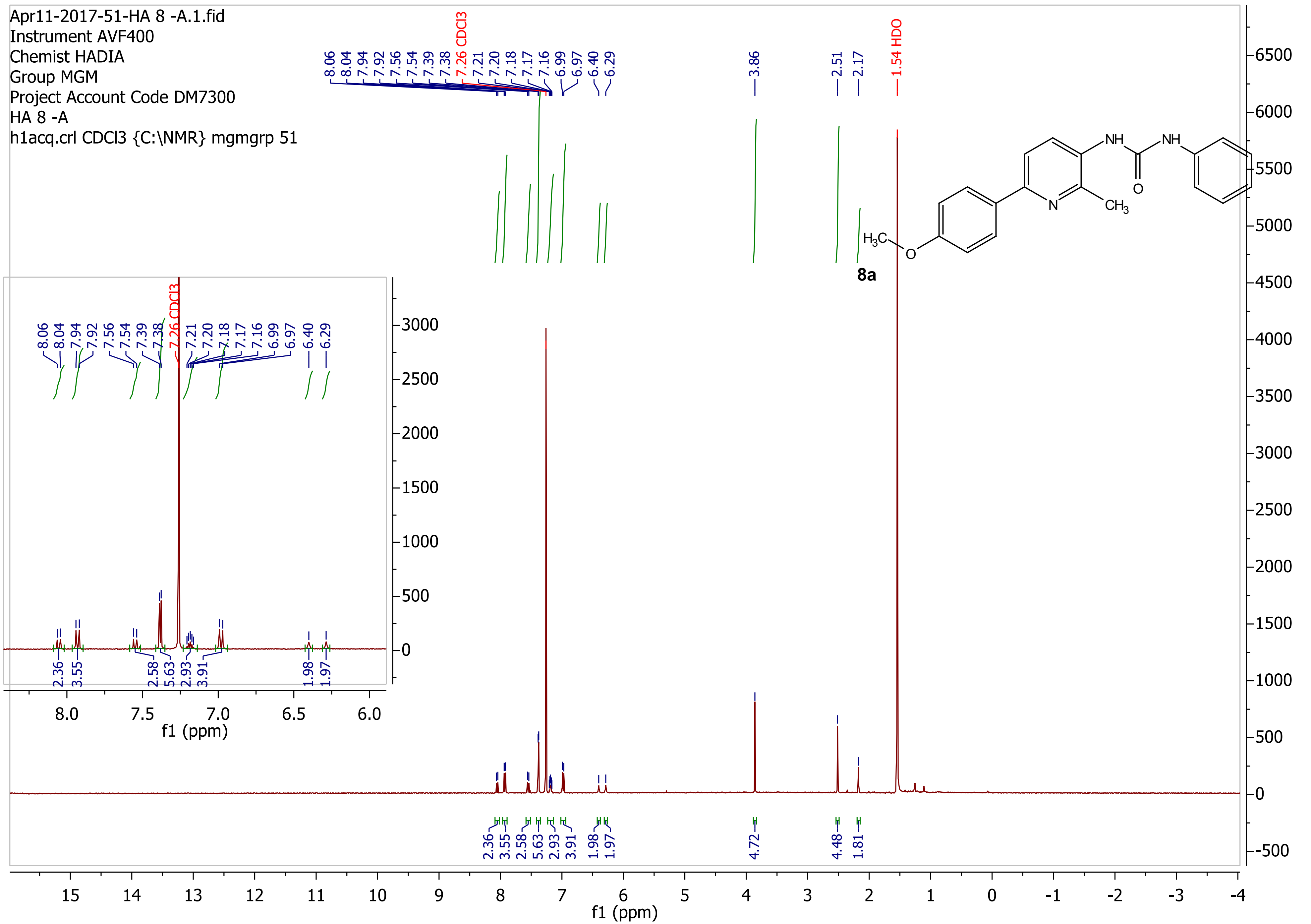
Apr11-2017-51-HA 8 -A.1.fid
Instrument AVF400
Chemist HADIA
Group MGM
Project Account Code DM7300
HA 8 -A
h1acq.crl CDCl3 {C:\NMR} mgmgrp 51
8.06 8.04 7.94 7.92 7.56 7.54 7.39 7.38 7.26 CDCl3 7.21 7.20 7.18 7.17 7.16 6.99 6.97 6.40 6.29
3.86
2.51
2.17
1.54 HDO
NH
NH
O
N
CH3
H3C
O
8a
2.36 3.55 2.58 5.63 2.93 3.91 1.98 1.97
4.72
4.48
1.81
f1 (ppm)

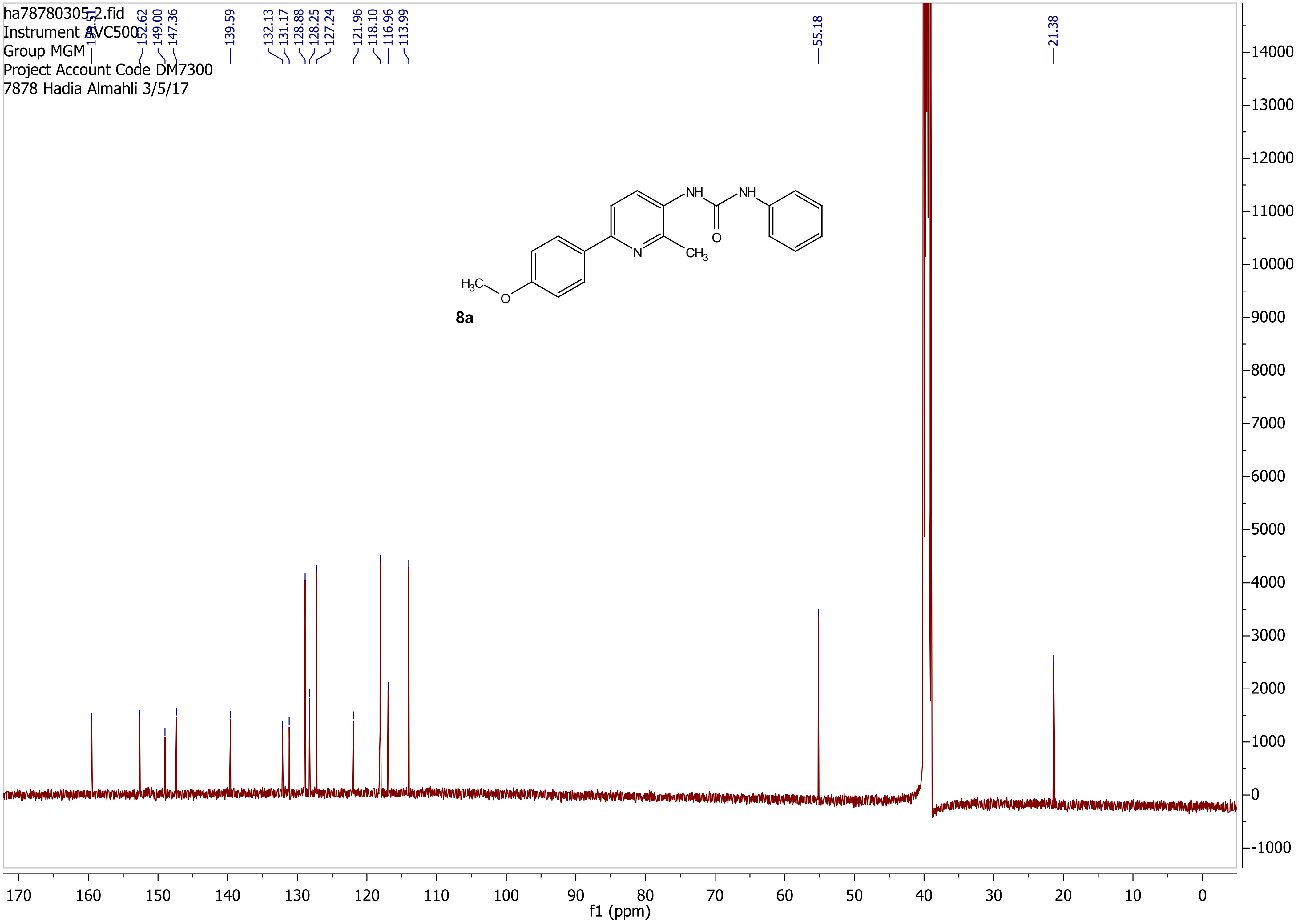
ha78780305_2.fid
Instrument AVC500
Group MGM
Project Account Code DM7300
7878 Hadia Almahli 3/5/17
152.62
149.00
147.36
139.59
132.13
131.17
128.88
128.25
127.24
121.96
118.10
116.96
113.99
55.18
21.38
NH
NH
O
N
CH3
H3C
O
8a
f1 (ppm)

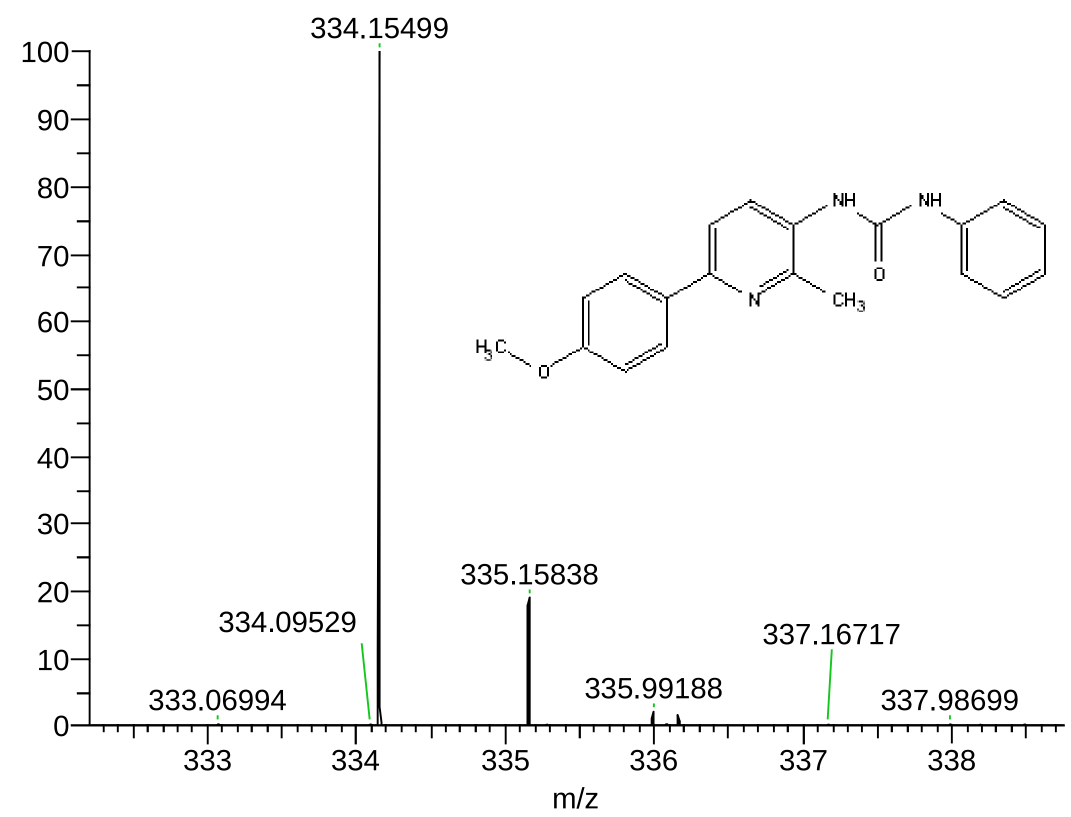

NL: 1.20E6
ESI64320 #13-26 RT: 0.14-0.29 AV: 7 NL: 2.52E7
T: FTMS {1,1} + p ESI Full ms [80.00-1600.00]

Measured Spectrum

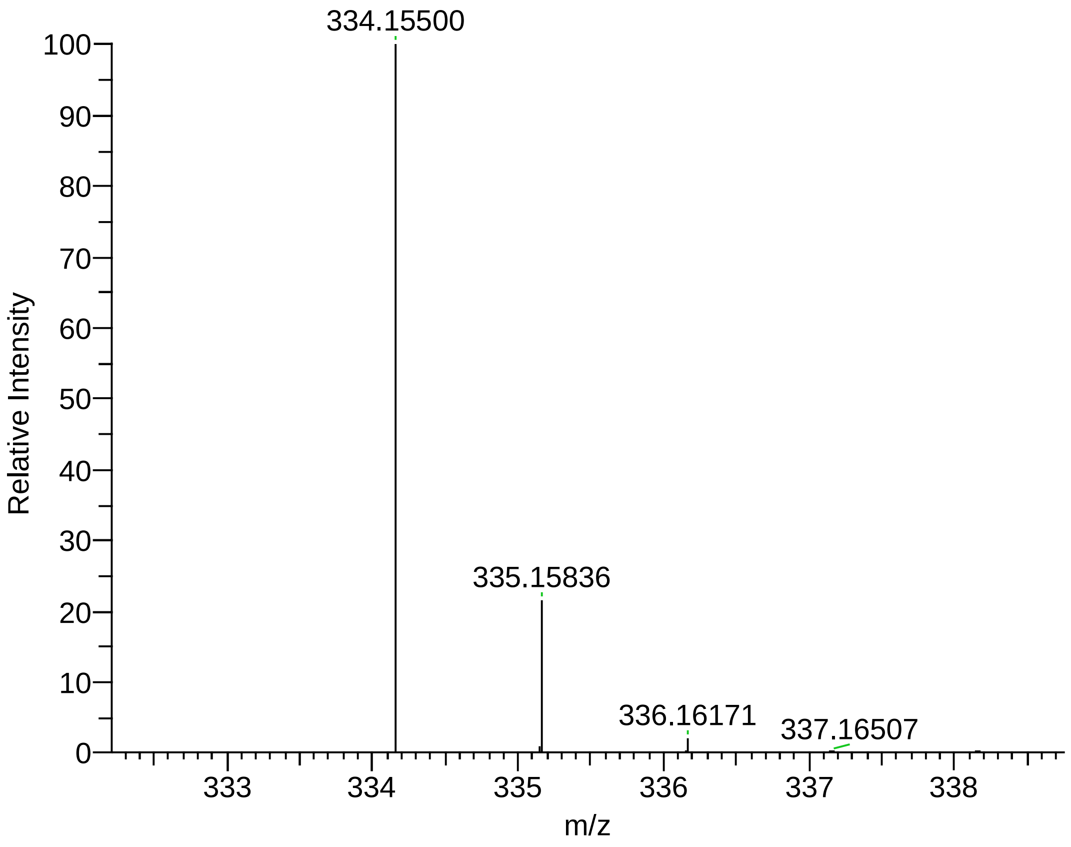

NL: 7.92E5
C20H20O2N3: C_{20} H_{20} O_2 N_3 Chrg 1 R: 1000000 Res. Pwr. @FWHM

Theoretical Spectrum

m/z	Formula	RDB	Delta ppm	Theo. Mass
334.15500	$C_{20}H_{20}O_2N_3$	12.5	-0.01	334.15500

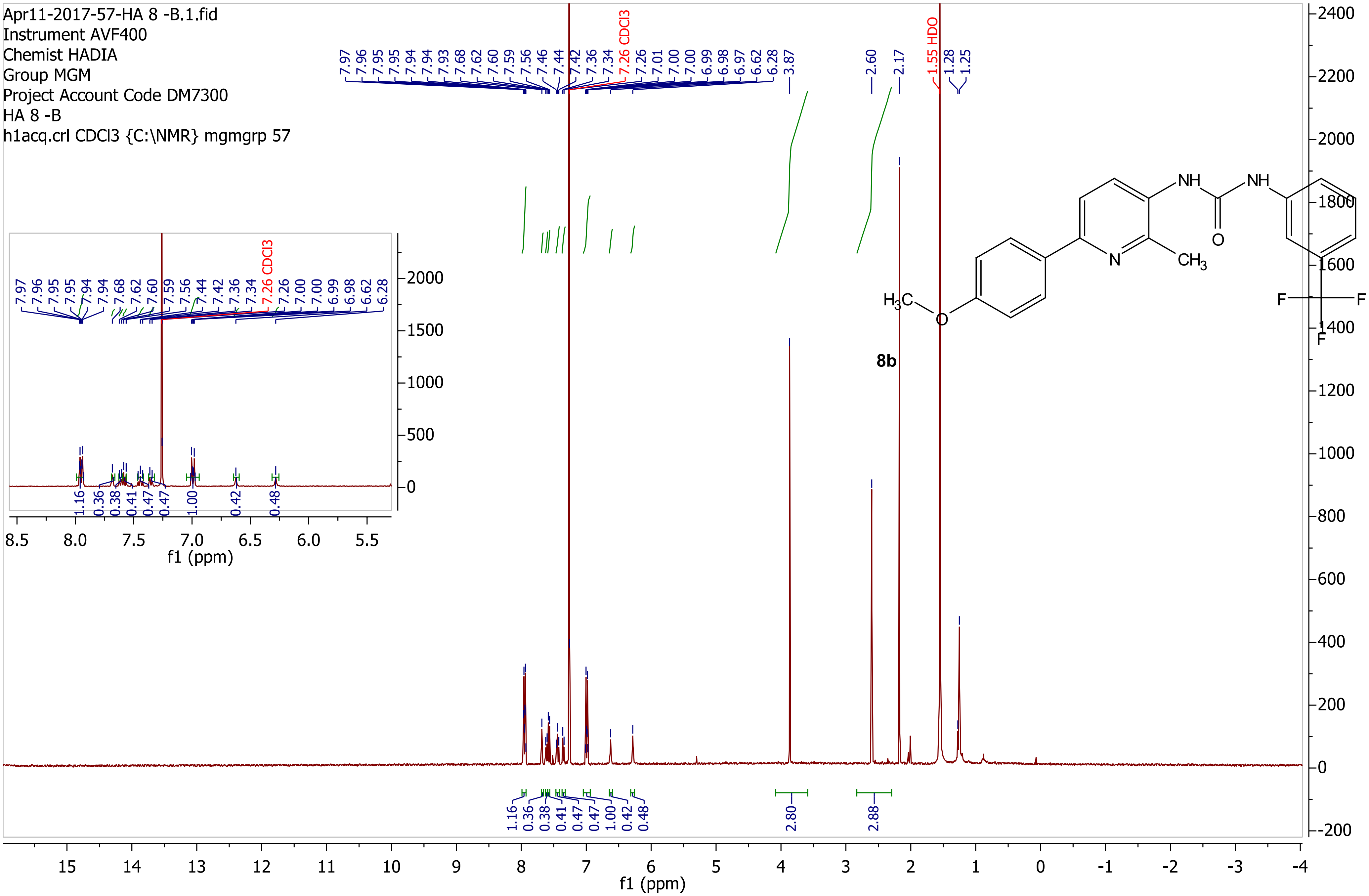

Apr11-2017-57-HA 8 -B.1.fid
Instrument AVF400
Chemist HADIA
Group MGM
Project Account Code DM7300
HA 8 -B
h1acq.crl CDCl3 {C:\NMR} mgmgrp 57
7.26 CDCl3
1.55 HDO
3.87
2.60
2.17
1.28
1.25
8b
f1 (ppm)

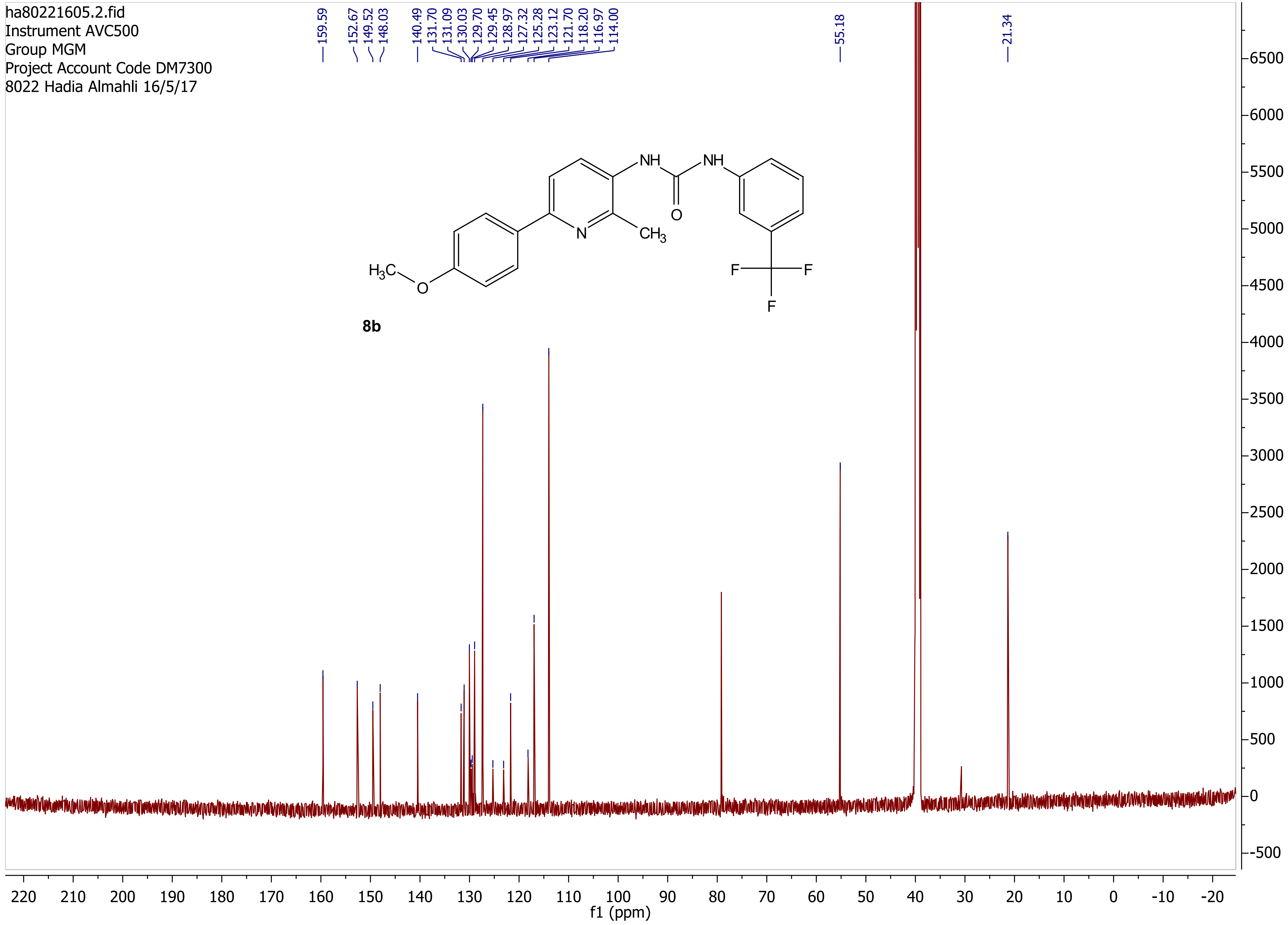
ha80221605.2.fid
Instrument AVC500
Group MGM
Project Account Code DM7300
8022 Hadia Almahli 16/5/17
159.59
152.67
149.52
148.03
140.49
131.70
131.09
130.03
129.70
129.45
128.97
127.32
125.28
123.12
121.70
118.20
116.97
114.00
55.18
21.34
NH
NH
O
N
CH3
H3C
O
F
F
F
8b
f1 (ppm)

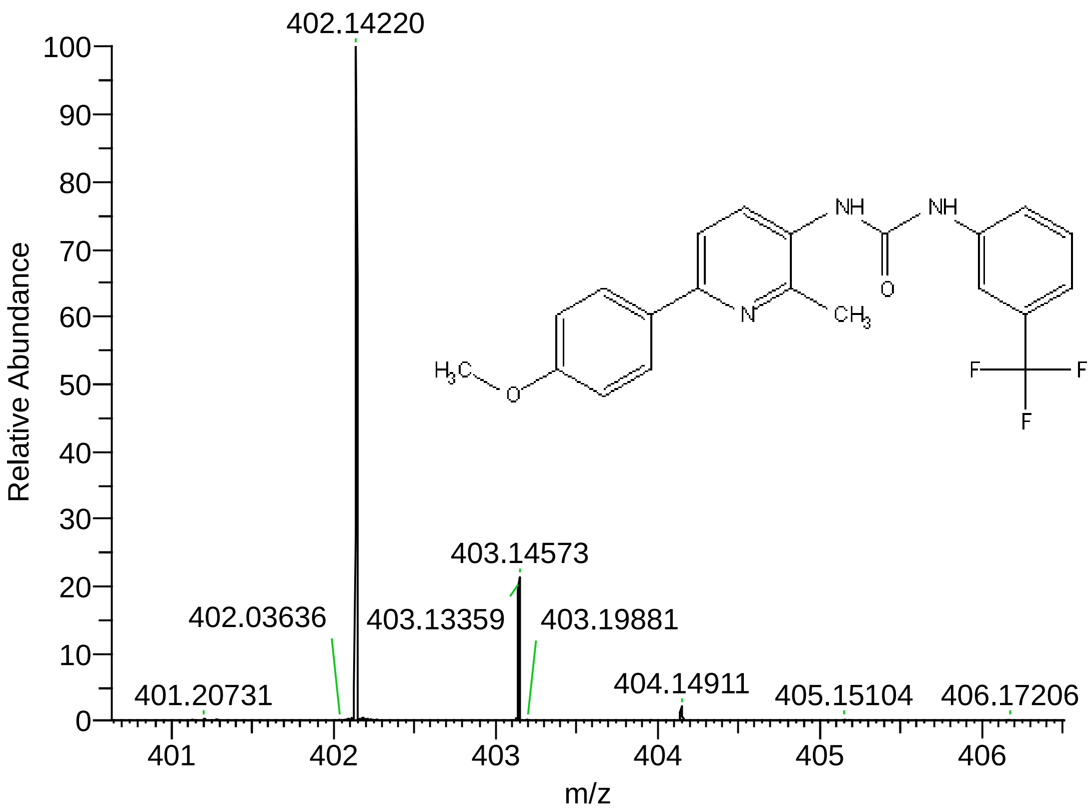

NL: 5.28E6
ESI64321 #15-25 RT: 0.17-0.28 AV: 6 NL: 3.05E+007
T: FTMS {1,1} + p ESI Full ms [80.00-1600.00]

Measured Spectrum

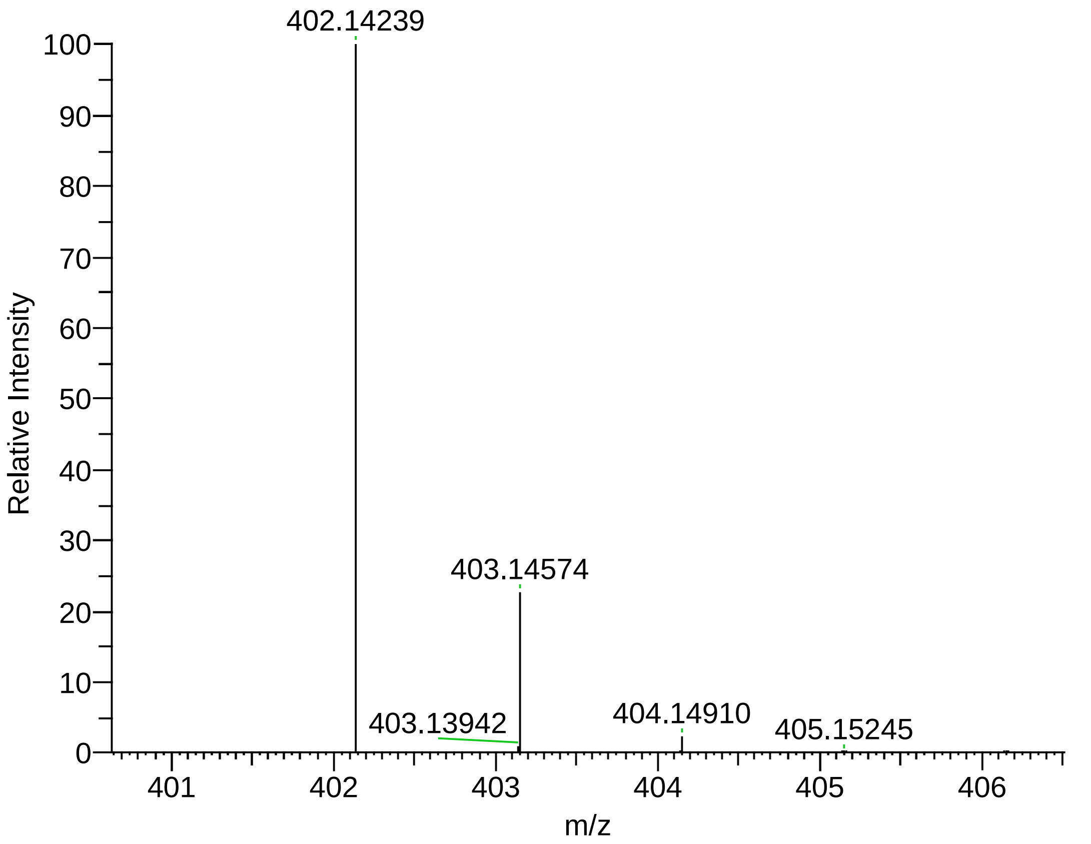

NL: 7.83E5
C21H19O2N3F3: C_{21} H_{19} O_2 N_3 F_3 Chrg 1
R: 1000000 Res. Pwr. @FWHM

Theoretical Spectrum

m/z	Formula	RDB	Delta ppm	Theo. Mass
402.14221	$C_{21}H_{19}O_2N_3F_3$	12.5	-0.44	402.14239

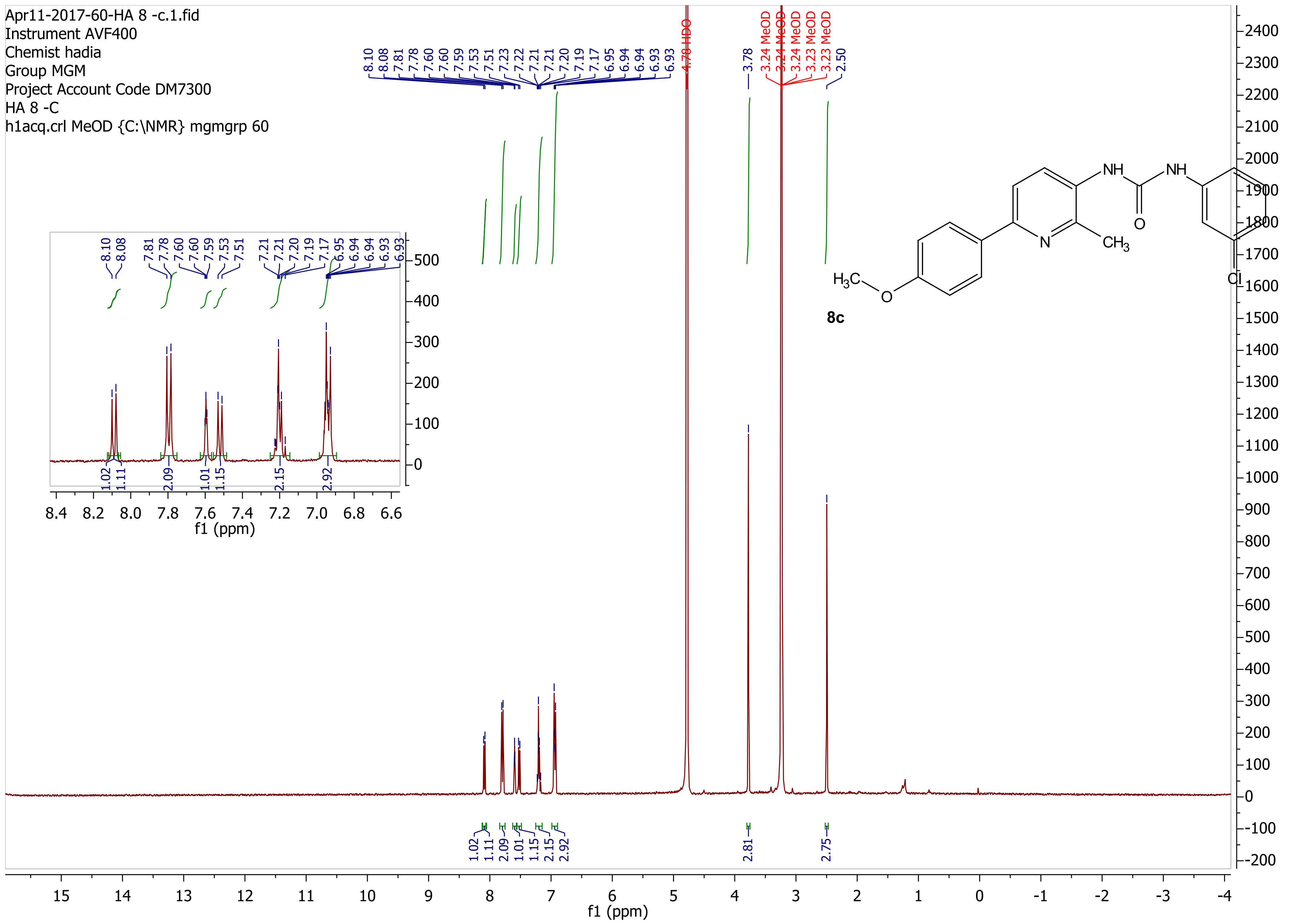
Apr11-2017-60-HA 8 -c.1.fid
Instrument AVF400
Chemist hadia
Group MGM
Project Account Code DM7300
HA 8 -C
h1acq.crl MeOD {C:\NMR} mgmgrp 60
8.10 8.08 7.81 7.78 7.60 7.60 7.59 7.53 7.51 7.23 7.22 7.21 7.21 7.20 7.19 7.17 6.95 6.94 6.94 6.93 6.93
4.78 HDO
3.78
3.24 MeOD 3.24 MeOD 3.24 MeOD 3.23 MeOD 3.23 MeOD
2.50
8c
1.02 1.11 2.09 1.01 1.15 2.15 2.92 2.81 2.75
f1 (ppm)

ha78800405.2.fid
Instrument AVC500
Group MGM
Project Account Code DM7300
7880 Hadia Almahli 4/5/17

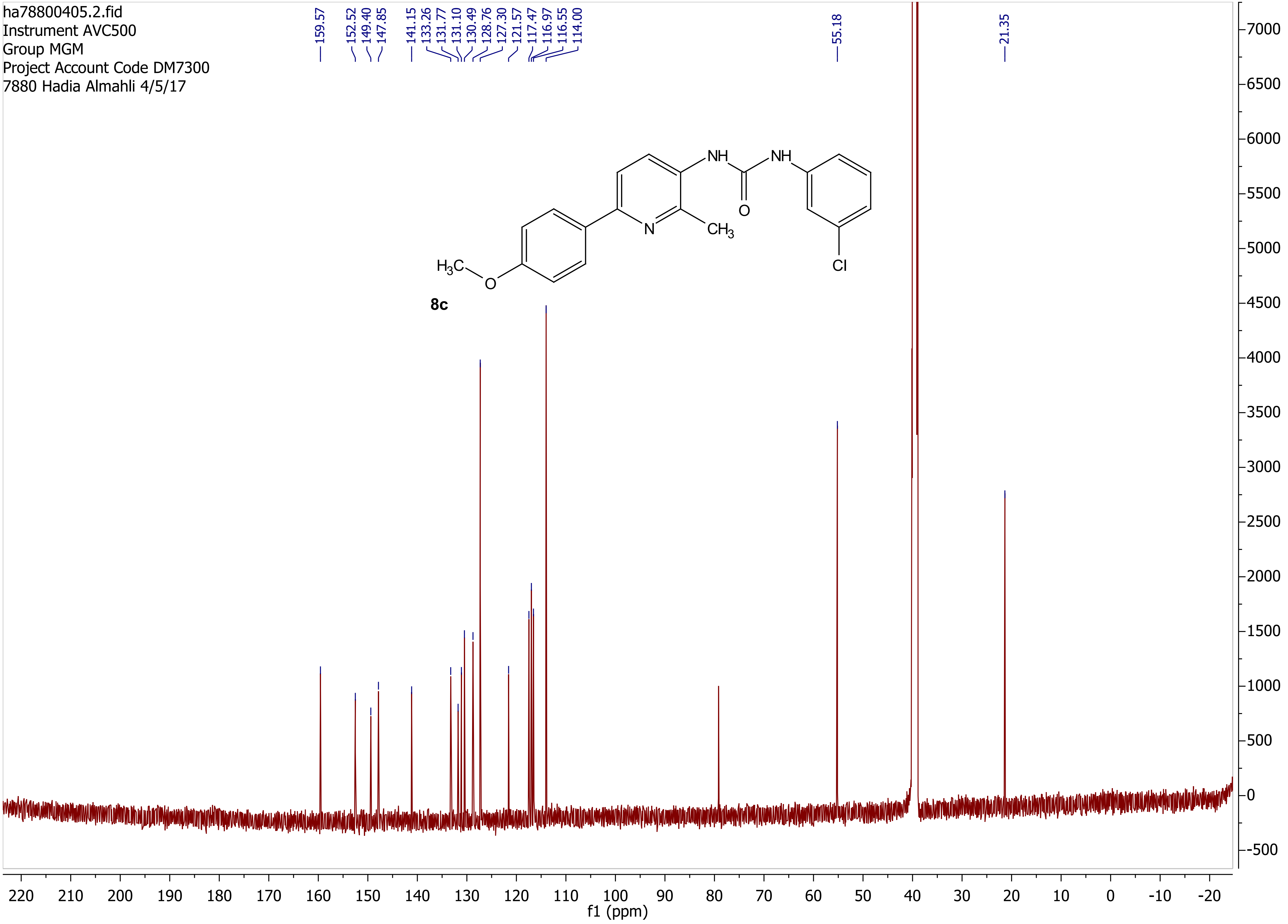

NL: 1.63E6
ESI64322 #15-25 RT: 0.17-0.28 AV: 6 NL: 3.25E+007
T: FTMS {1,1} + p ESI Full ms [80.00-1600.00]

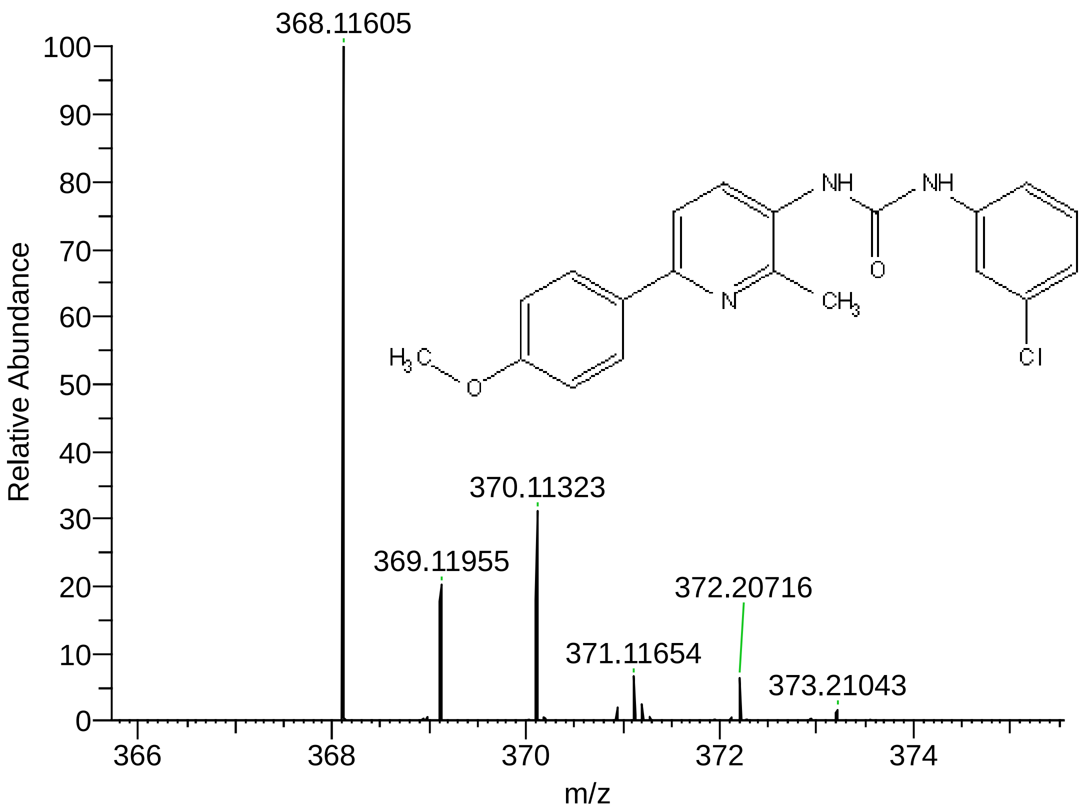

Measured Spectrum

NL: 6.00E5
C20H19O2N3Cl1: C_{20} H_{19} O_2 N_3 Cl Chrg 1
R: 1000000 Res. Pwr. @FWHM

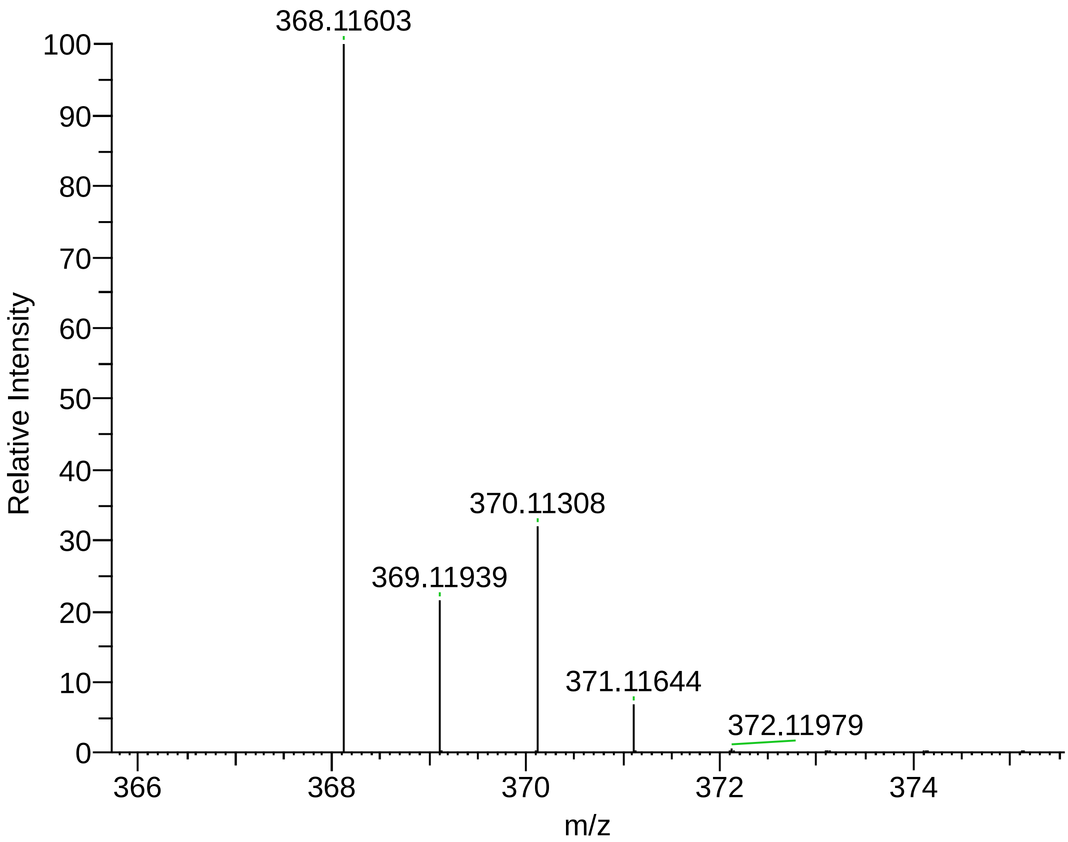

Theoretical Spectrum

m/z	Formula	RDB	Delta ppm	Theo. Mass
368.11606	$C_{20}H_{19}O_2N_3{}^{35}Cl$	12.5	0.07	368.11603

Apr11-2017-9-HA 8 -D.1.fid
Instrument AVF400
Chemist HADIA
Group MGM
Project Account Code DM7300
HA 8 -D
h1acq.crl CDCl3 {C:\NMR} mgmgrp 9

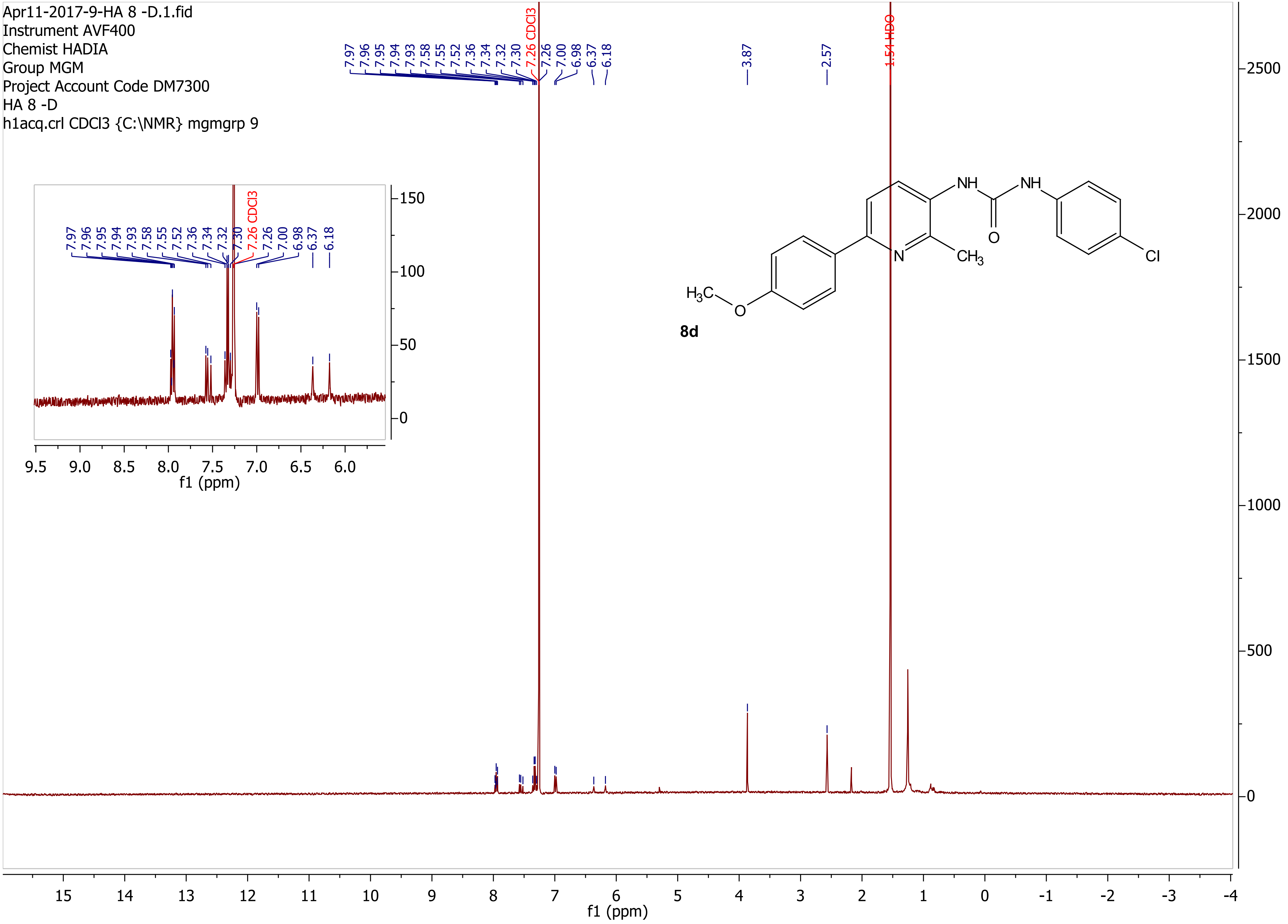

ha78760305.2.fid
Instrument AVC500
Group MGM
Project Account Code DM7300
7876 Hadia Almahli 3/5/17

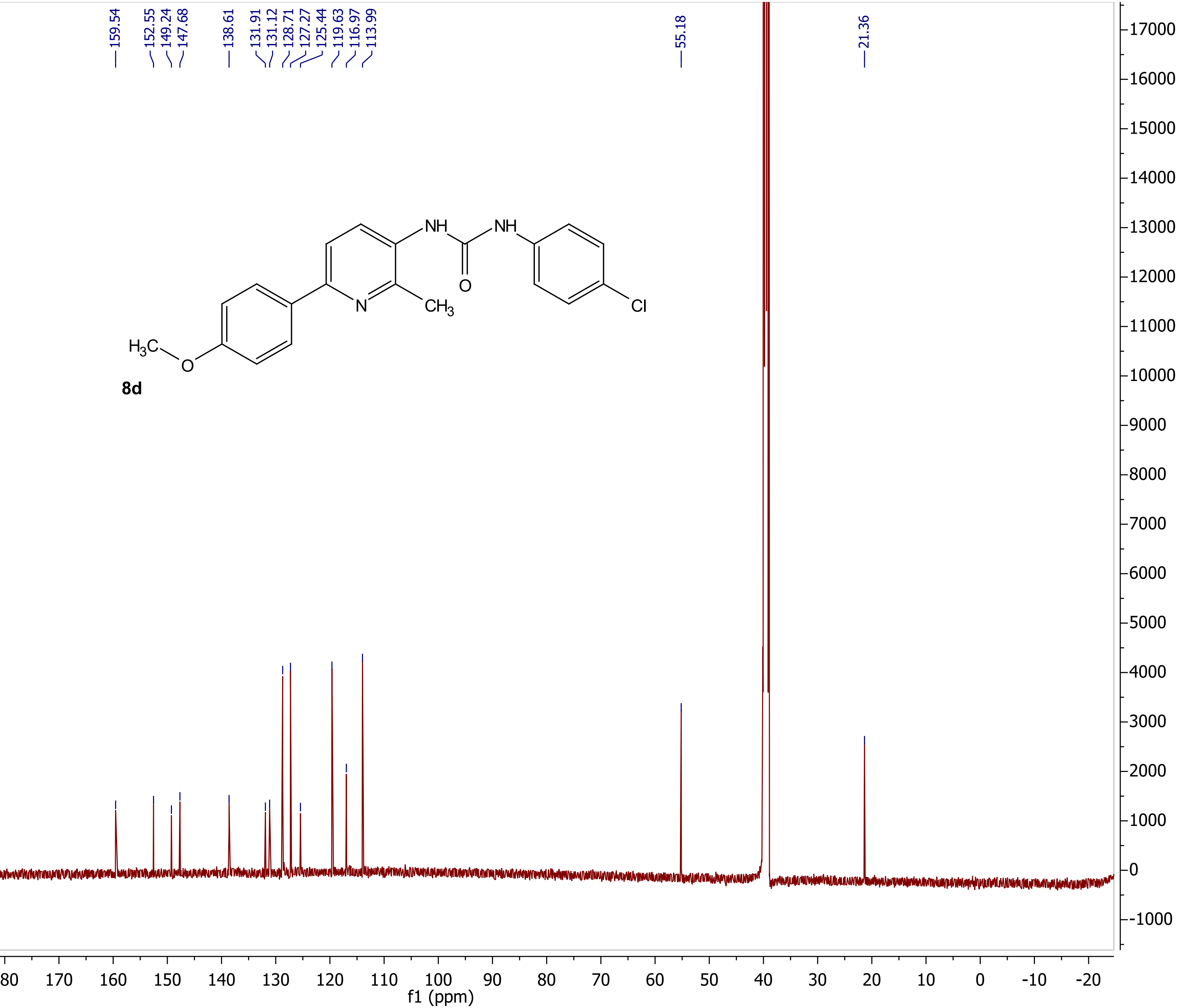

8d

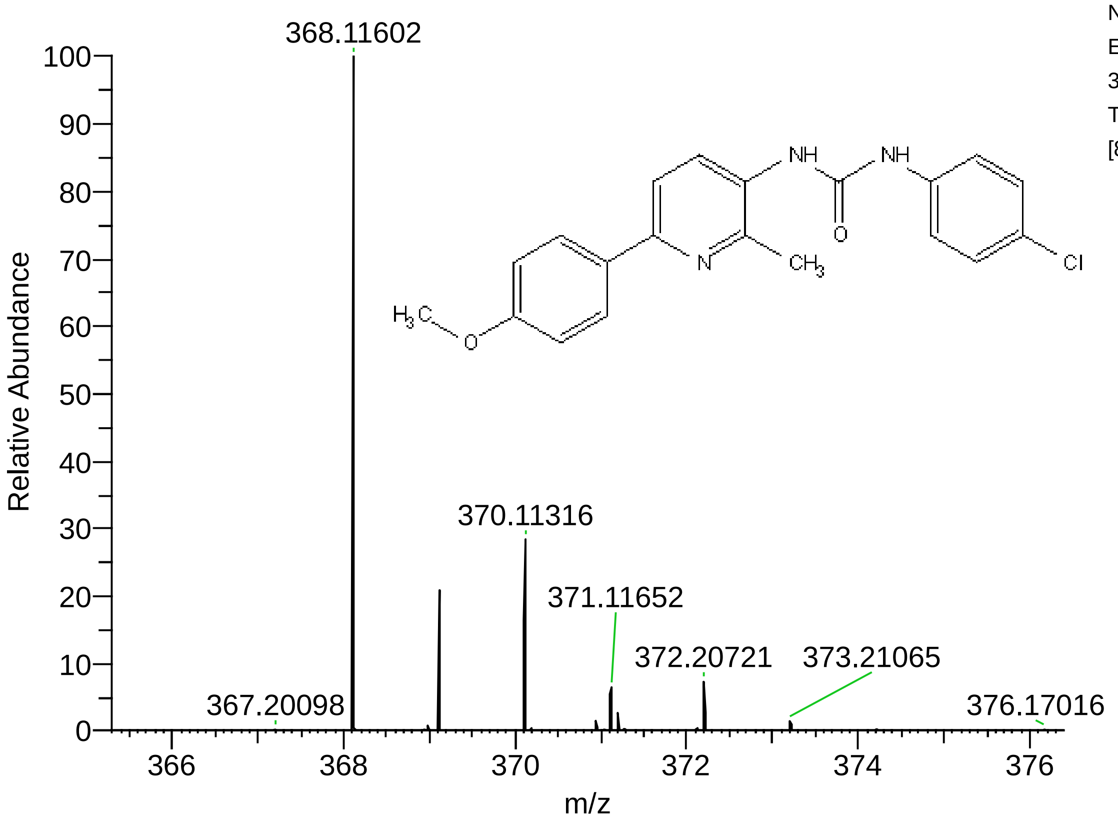

NL: 1.62E6
ESI64323 #15-25 RT: 0.17-0.28 AV: 6 NL: 3.43E+007
T: FTMS {1,1} + p ESI Full ms [80.00-1600.00]

Measured Spectrum

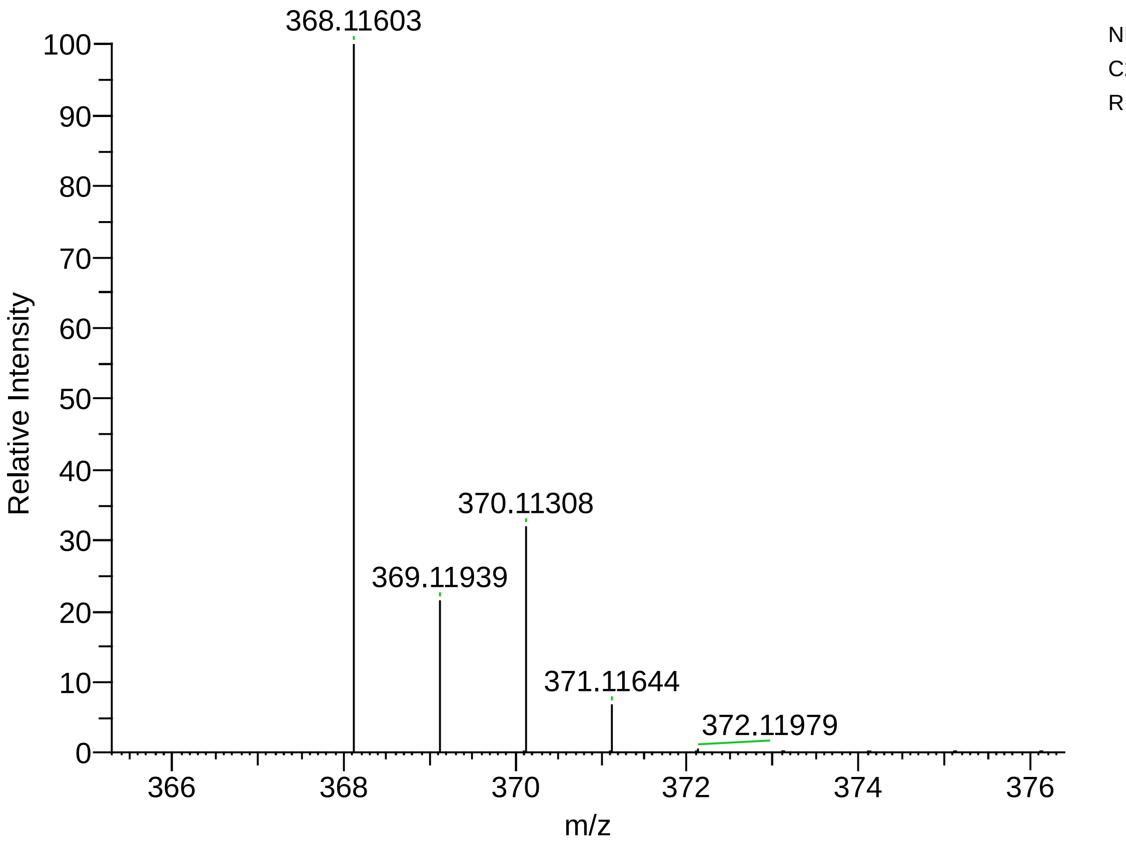

NL: 6.00E5
C20H19O2N3Cl1: C_{20} H_{19} O_2 N_3 Cl Chrg 1
R: 1000000 Res. Pwr. @FWHM

Theoretical Spectrum

m/z	Formula	RDB	Delta ppm	Theo. Mass
368.11603	$C_{20}H_{19}O_2N_3{}^{35}Cl$	12.5	-0.01	368.11603

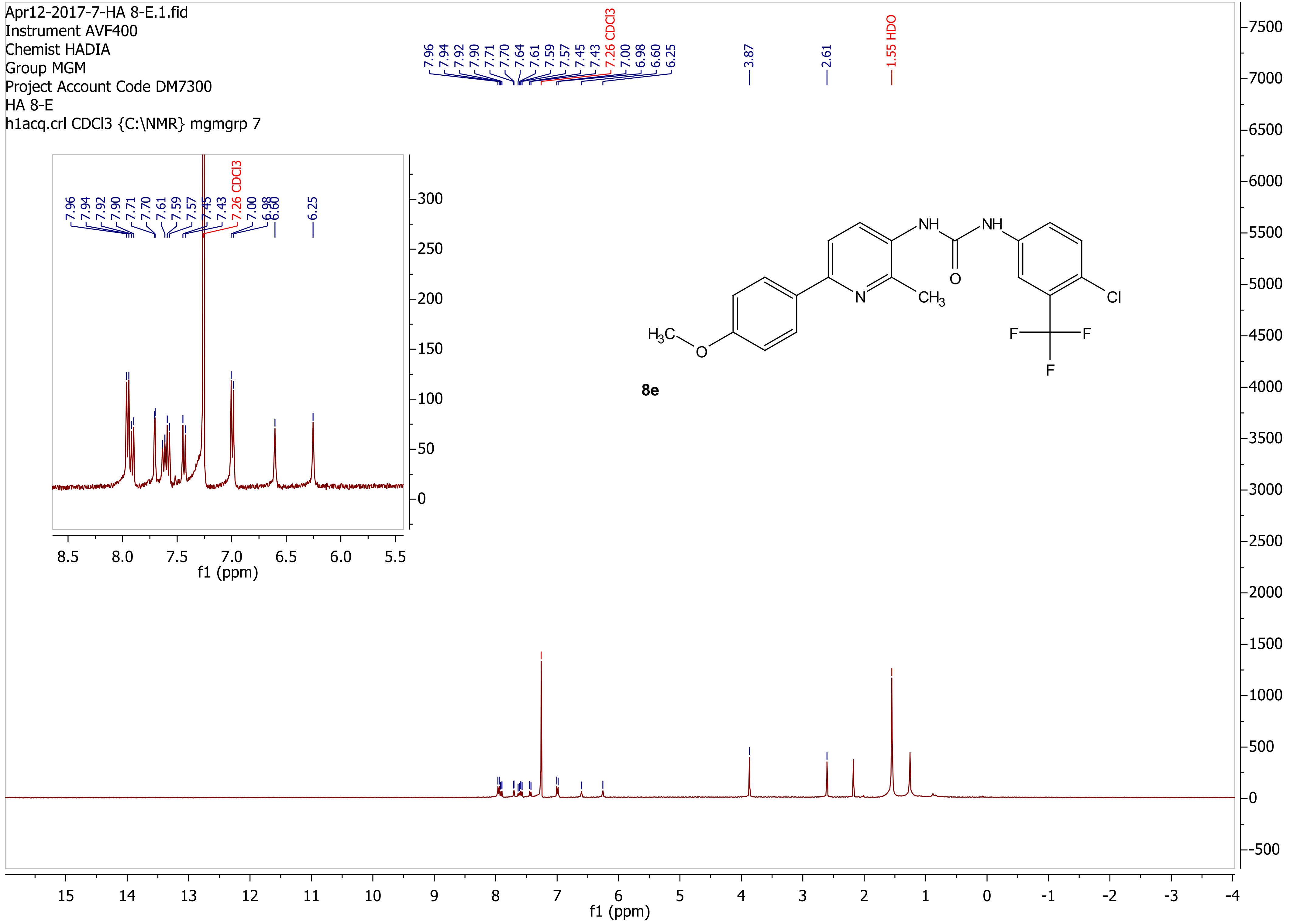
Apr12-2017-7-HA 8-E.1.fid
Instrument AVF400
Chemist HADIA
Group MGM
Project Account Code DM7300
HA 8-E
h1acq.crl CDCl3 {C:\NMR} mgmgrp 7
7.96 7.94 7.92 7.90 7.71 7.70 7.64 7.61 7.59 7.57 7.45 7.43 7.26 CDCl3 7.00 6.98 6.60 6.25
3.87
2.61
1.55 HDO
8e
f1 (ppm)

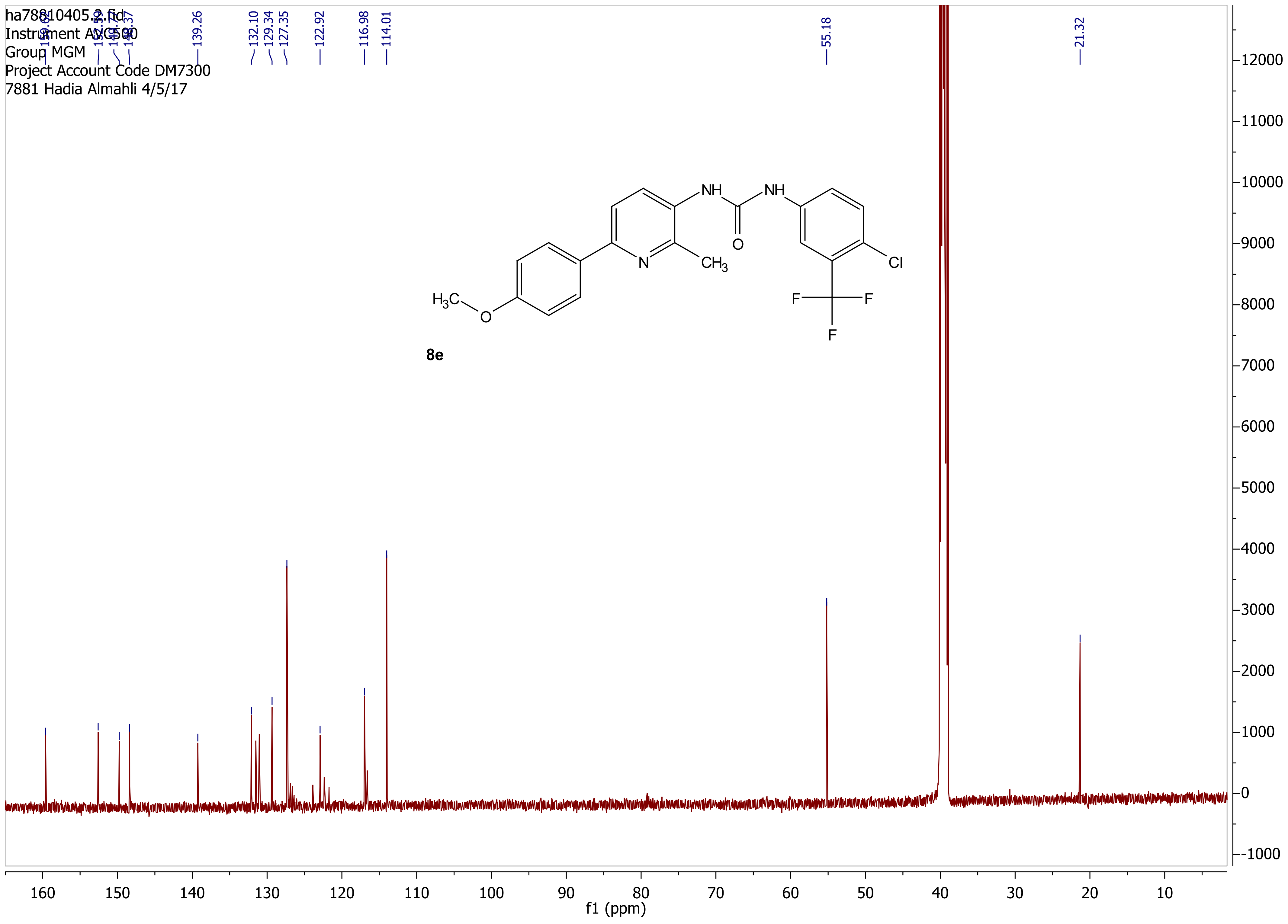
ha78810405.2.fid
Instrument AV500
Group MGM
Project Account Code DM7300
7881 Hadia Almahli 4/5/17
159.62
152.59
149.77
148.37
139.26
132.10
129.34
127.35
122.92
116.98
114.01
55.18
21.32
NH
NH
O
N
CH3
Cl
H3C
O
F
F
F
8e
f1 (ppm)

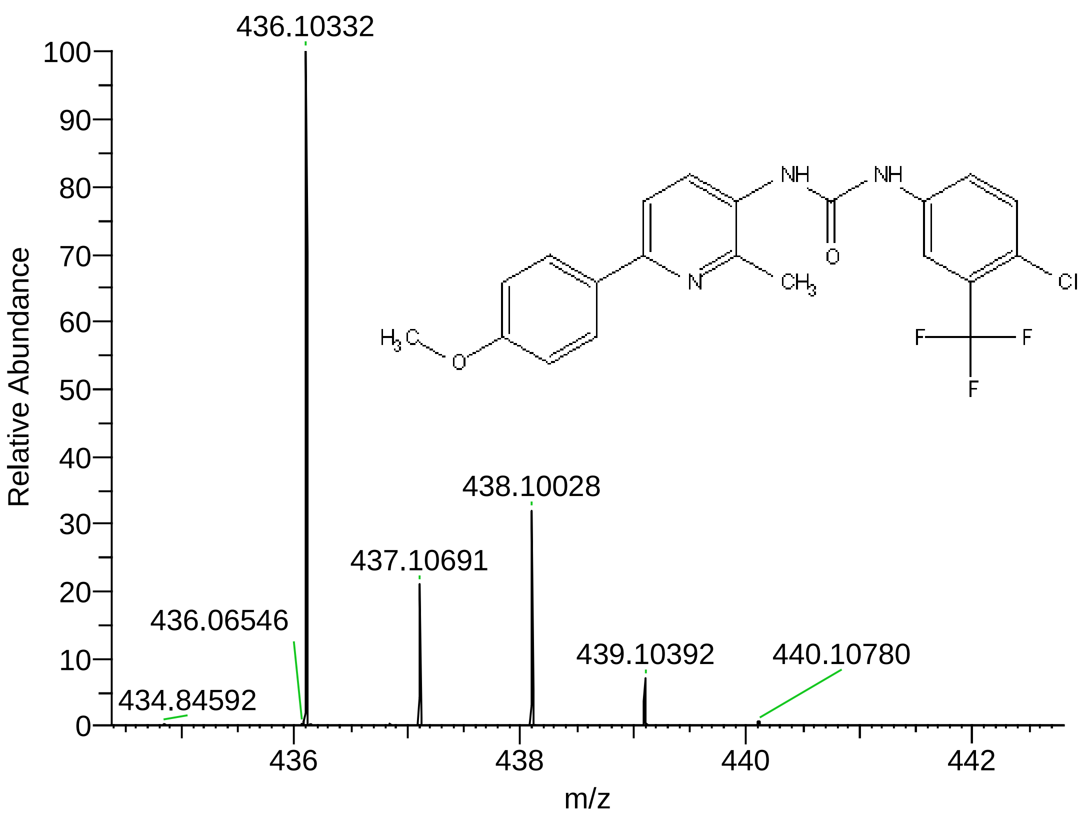

NL: 1.79E6
ESI64324 #15-25 RT: 0.17-0.28 AV: 6 NL: 3.14E+007
T: FTMS {1,1} + p ESI Full ms [80.00-1600.00]

Measured Spectrum

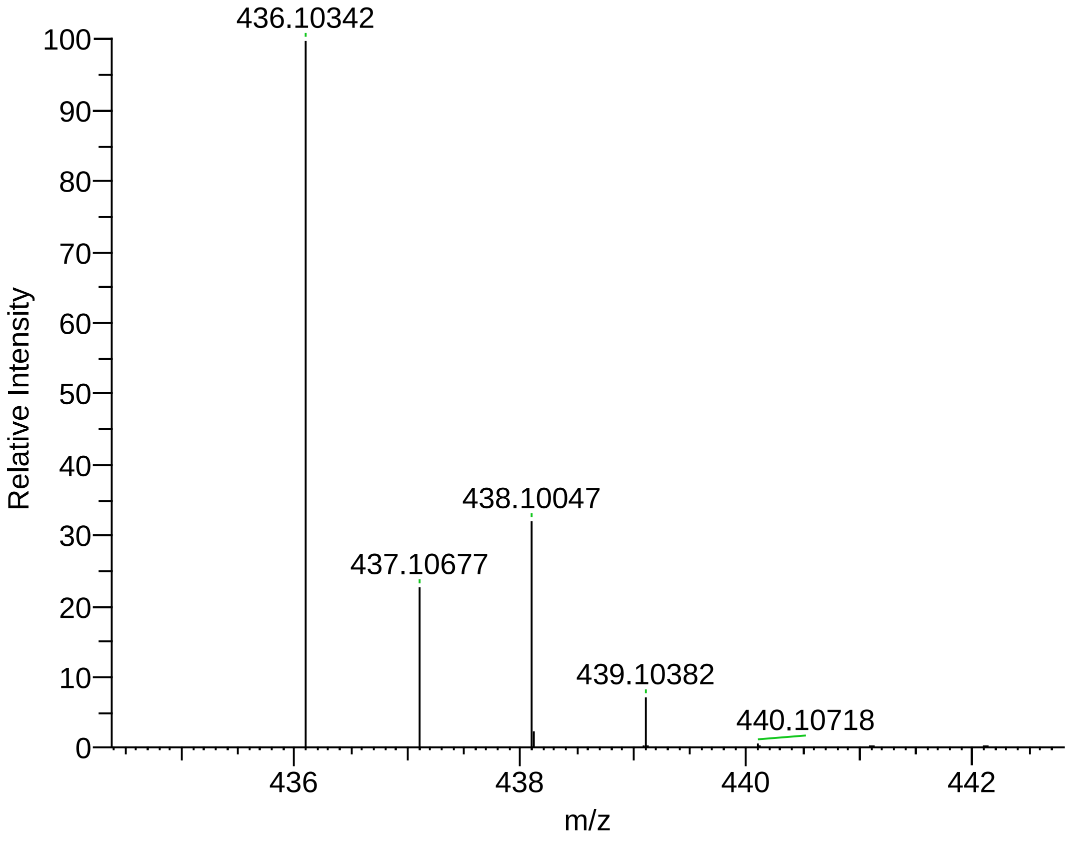

NL: 5.94E5
C21H18O2N3Cl1F3: $C_{21}H_{18}O_2N_3ClF_3$
Chrg 1 R: 1000000 Res. Pwr. @FWHM

Theoretical Spectrum

m/z	Formula	RDB	Delta ppm	Theo. Mass
436.10330	$C_{21}H_{18}O_2N_3{}^{35}ClF_3$	12.5	-0.26	436.10342

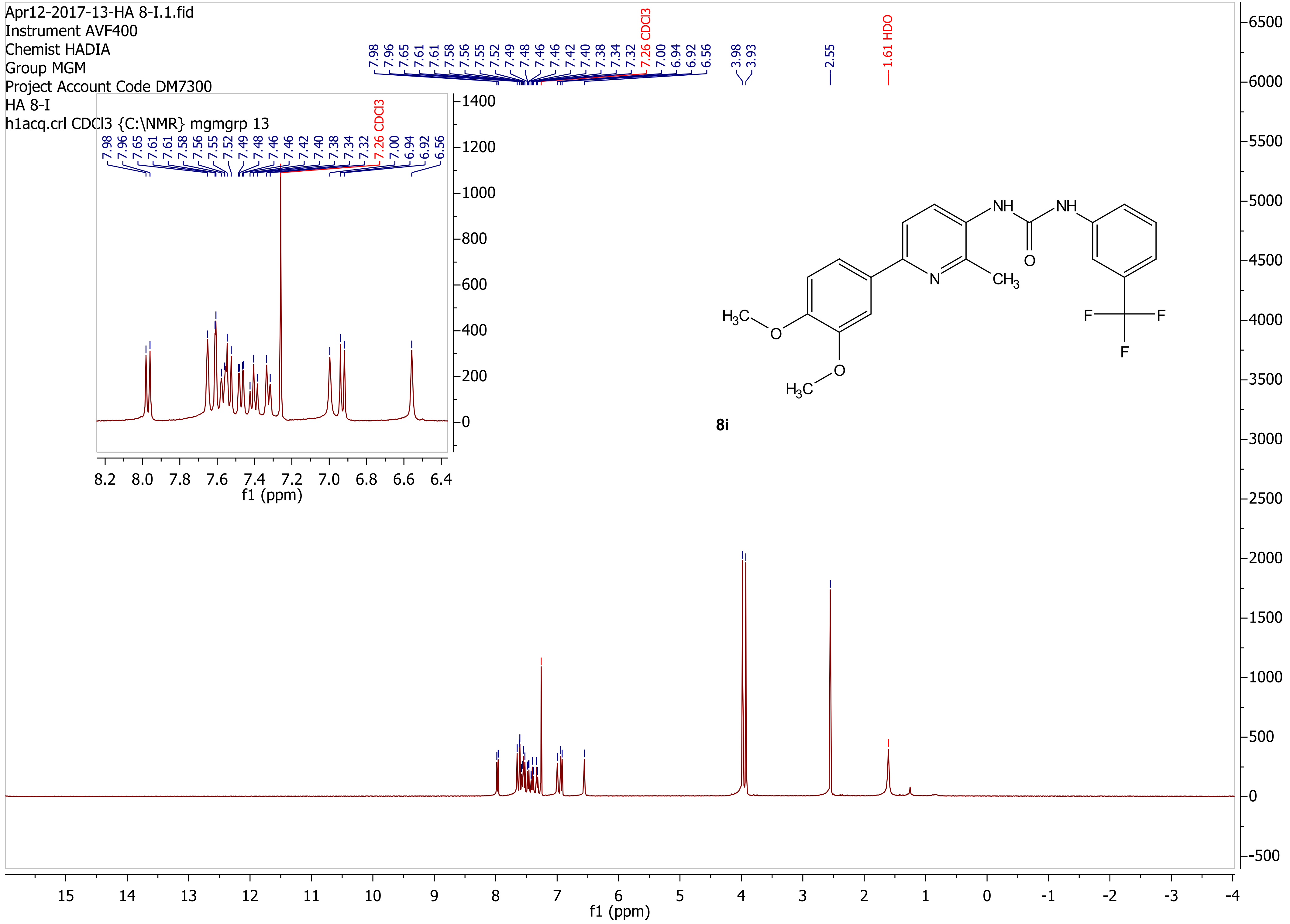

Apr12-2017-13-HA 8-I.1.fid
Instrument AVF400
Chemist HADIA
Group MGM
Project Account Code DM7300
HA 8-I
h1acq.crl CDCl3 {C:\NMR} mgmgrp 13
7.98 7.96 7.65 7.61 7.61 7.58 7.56 7.55 7.52 7.49 7.48 7.46 7.46 7.42 7.40 7.38 7.34 7.32 7.26 CDCl3 7.00 6.94 6.92 6.56
3.98 3.93
2.55
1.61 HDO
f1 (ppm)
NH
NH
O
N
CH3
H3C
O
O
H3C
F
F
F
8i

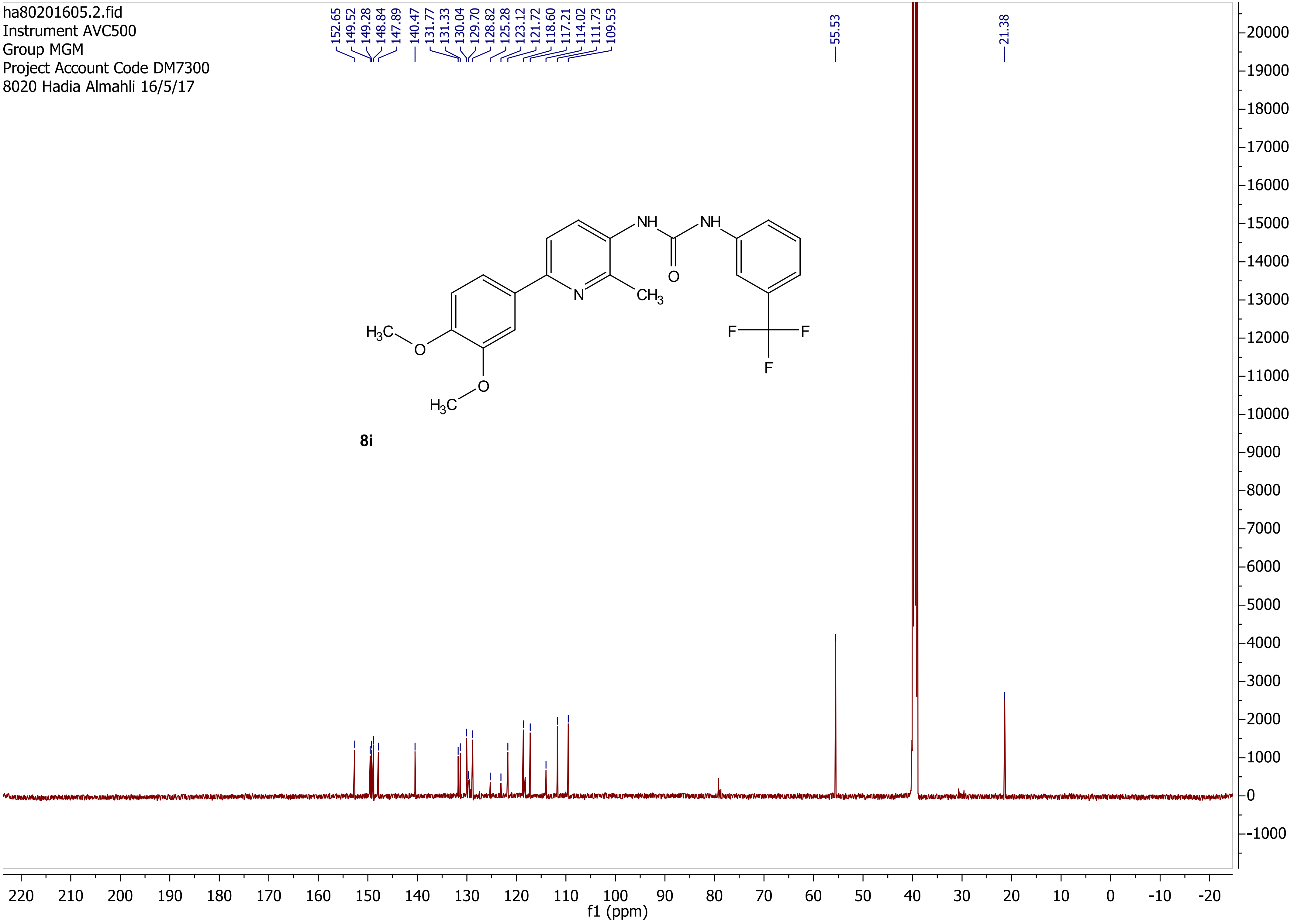
ha80201605.2.fid
Instrument AVC500
Group MGM
Project Account Code DM7300
8020 Hadia Almahli 16/5/17
152.65
149.52
149.28
148.84
147.89
140.47
131.77
131.33
130.04
129.70
128.82
125.28
123.12
121.72
118.60
117.21
114.02
111.73
109.53
55.53
21.38
NH
NH
O
N
CH3
H3C
O
O
H3C
F
F
F
8i
20000
19000
18000
17000
16000
15000
14000
13000
12000
11000
10000
9000
8000
7000
6000
5000
4000
3000
2000
1000
0
-1000
220 210 200 190 180 170 160 150 140 130 120 110 100 90 80 70 60 50 40 30 20 10 0 -10 -20
f1 (ppm)

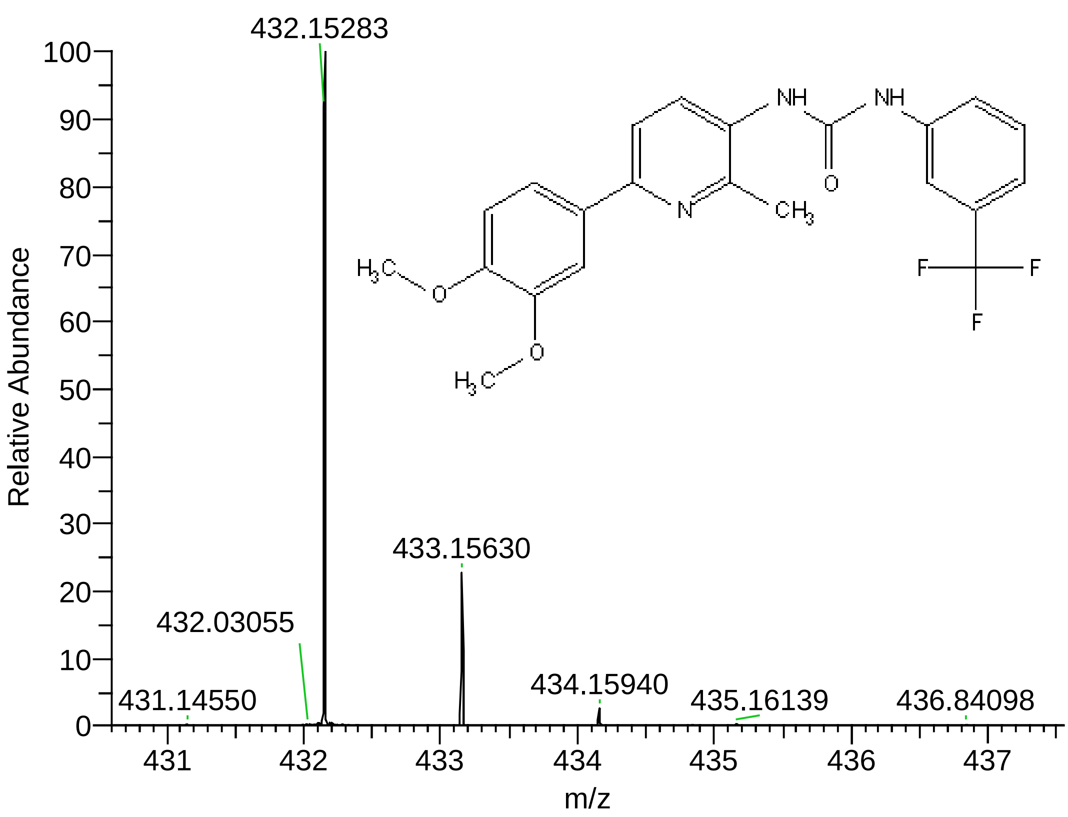

NL: 6.97E6
ESI64328 #15-25 RT: 0.17-0.28 AV: 6 NL: 3.11E+007
T: FTMS {1,1} + p ESI Full ms [80.00-1600.00]

Measured Spectrum

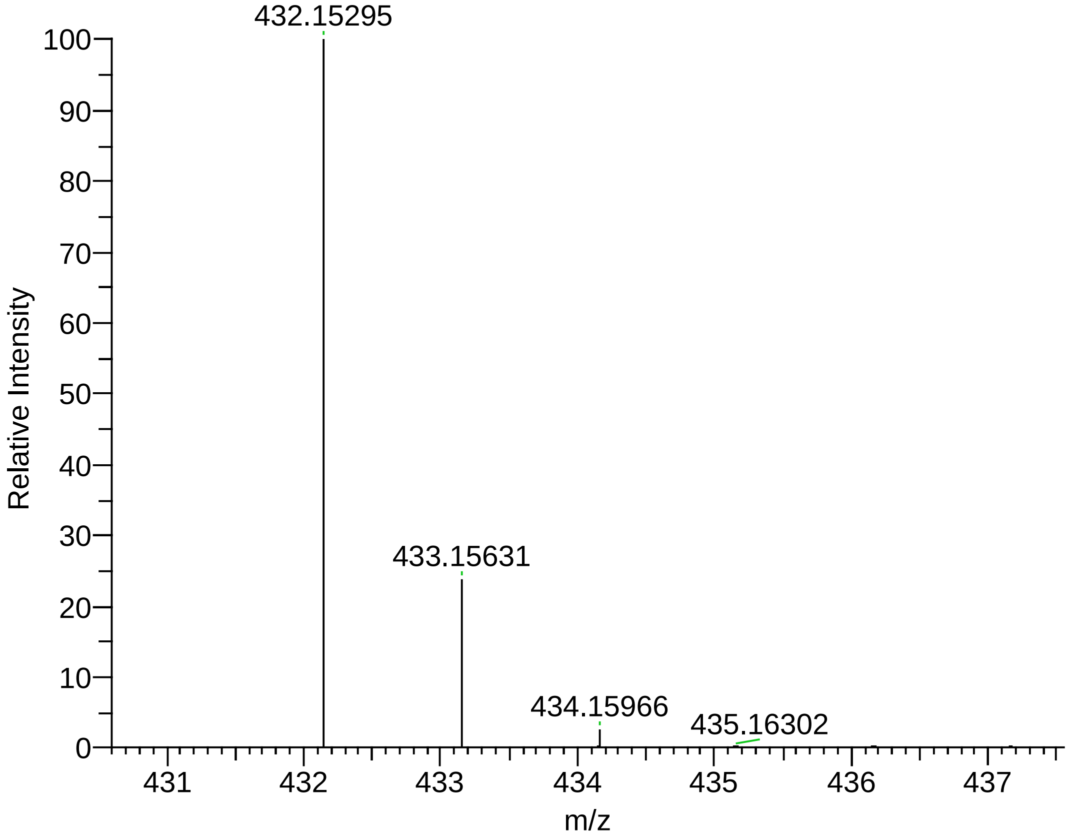

NL: 7.73E5
C22H21O3N3F3: C_{22} H_{21} O_3 N_3 F_3 Chrg 1
R: 1000000 Res. Pwr. @FWHM

Theoretical Spectrum

m/z	Formula	RDB	Delta ppm	Theo. Mass
432.15283	$C_{22}H_{21}O_3N_3F_3$	12.5	-0.28	432.15295

Apr12-2017-14-HA 8-J.1.fid
Instrument AVF400
Chemist HADIA
Group MGM
Project Account Code DM7300
HA 8-J
h1acq.crl CDCl3 {C:\NMR} mgmgrp 14

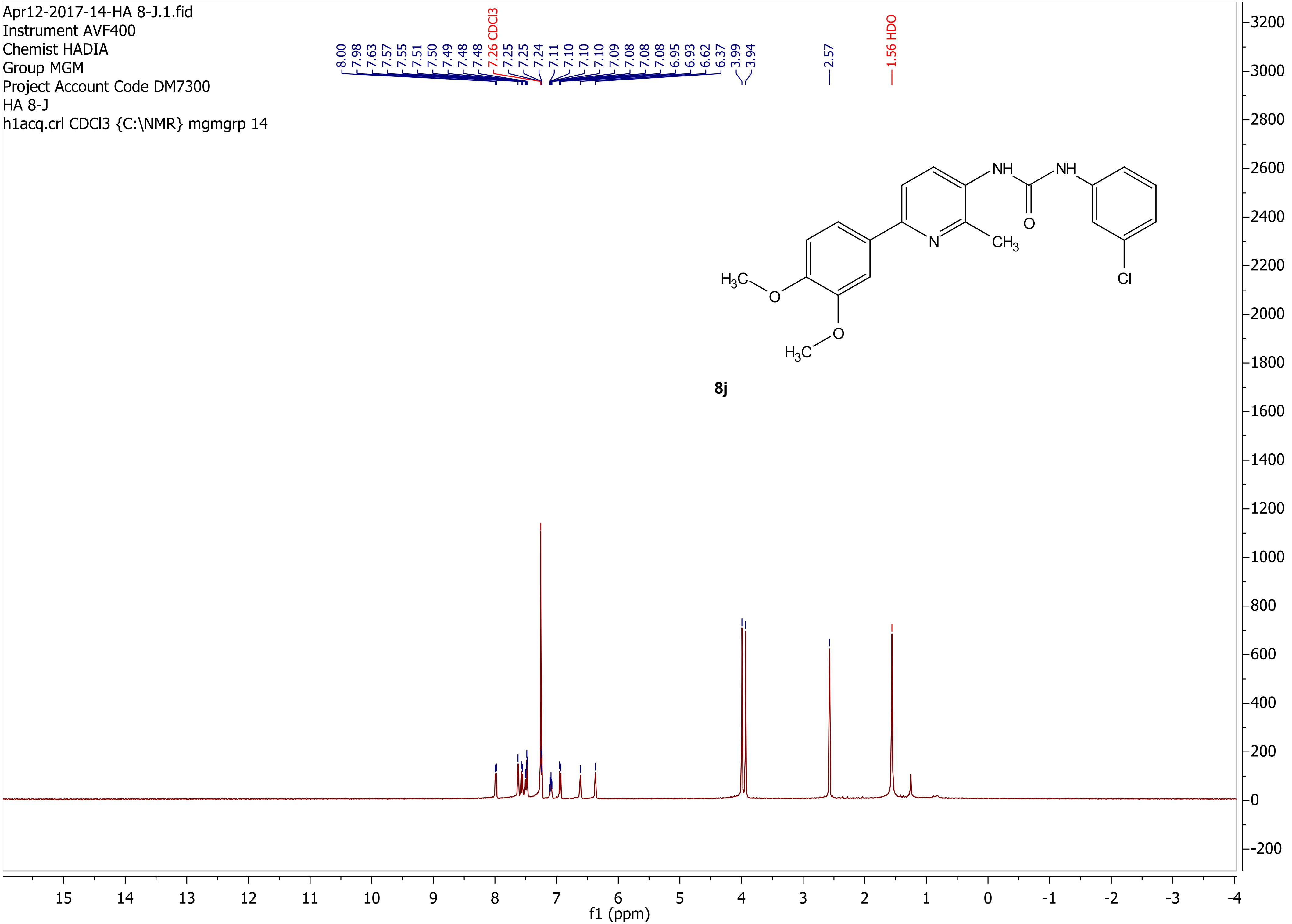

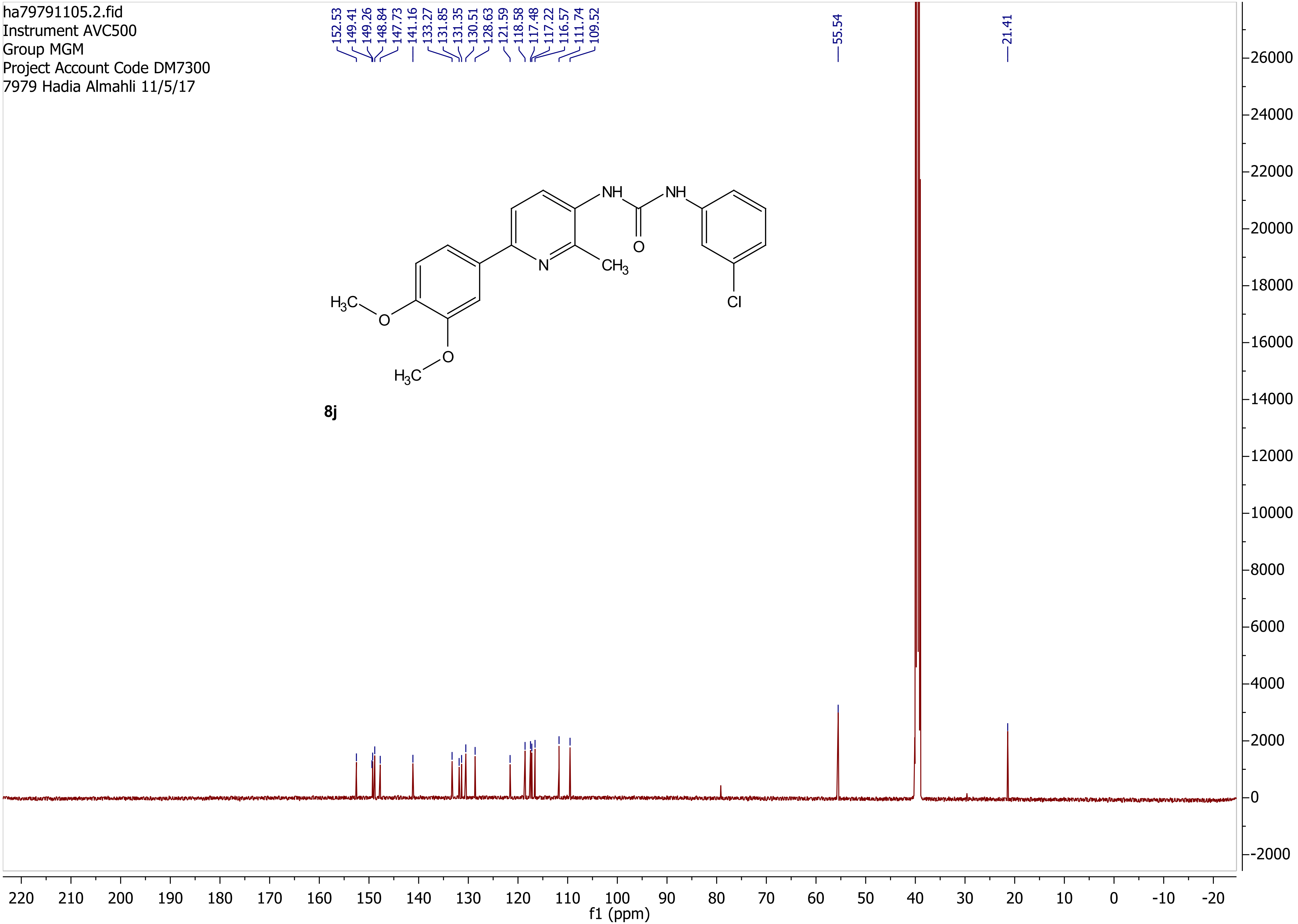

ha79791105.2.fid
Instrument AVC500
Group MGM
Project Account Code DM7300
7979 Hadia Almahli 11/5/17
152.53
149.41
149.26
148.84
147.73
141.16
133.27
131.85
131.35
130.51
128.63
121.59
118.58
117.48
117.22
116.57
111.74
109.52
55.54
21.41
NH
NH
O
N
CH3
Cl
H3C
O
O
H3C
8j
f1 (ppm)

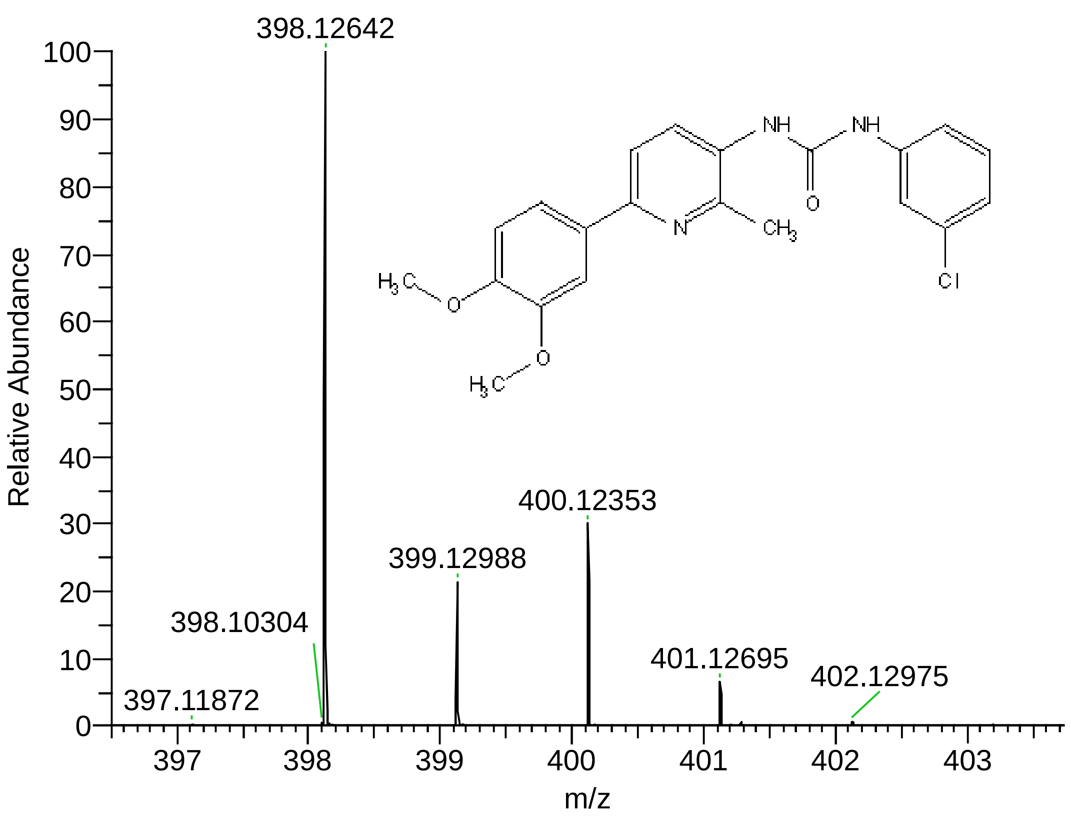

NL: 2.12E6
ESI64329 #15-25 RT: 0.17-0.28 AV: 6 NL: 2.93E+007
T: FTMS {1,1} + p ESI Full ms [80.00-1600.00]

Measured Spectrum

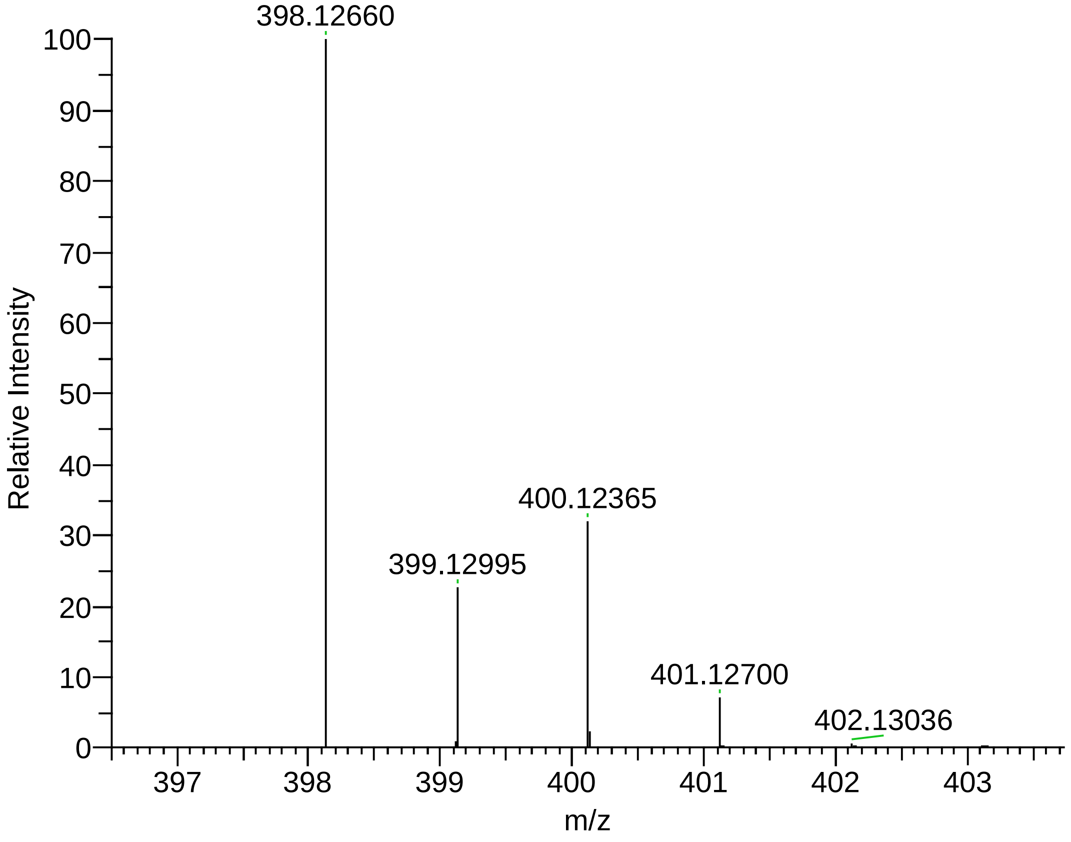

NL: 5.92E5
C21H21O3N3Cl1: C_{21} H_{21} O_3 N_3 Cl Chrg 1
R: 1000000 Res. Pwr. @FWHM

Theoretical Spectrum

m/z	Formula	RDB	Delta ppm	Theo. Mass
398.12643	$C_{21}H_{21}O_3N_3{}^{35}Cl$	12.5	-0.41	398.12660

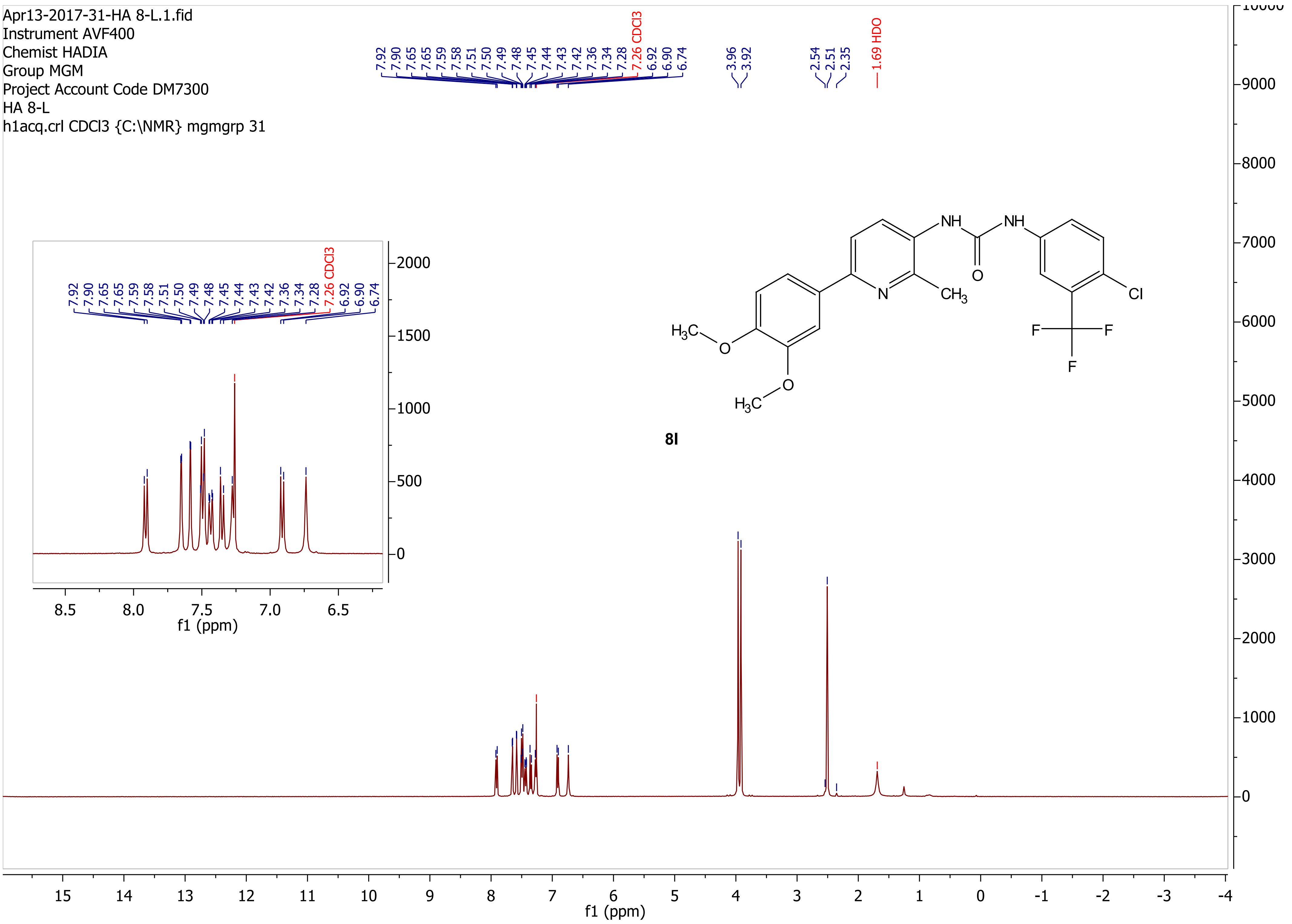
Apr13-2017-31-HA 8-L.1.fid
Instrument AVF400
Chemist HADIA
Group MGM
Project Account Code DM7300
HA 8-L
h1acq.crl CDCl3 {C:\NMR} mgmgrp 31
7.92 7.90 7.65 7.65 7.59 7.58 7.51 7.50 7.49 7.48 7.45 7.44 7.43 7.42 7.36 7.34 7.28 7.26 CDCl3 6.92 6.90 6.74
3.96 3.92
2.54 2.51 2.35
1.69 HDO
NH
NH
O
N
CH3
Cl
F
F
F
H3C
O
O
H3C
8l
f1 (ppm)
f1 (ppm)

ha79811105.2.fid
Instrument AVC500
Group MGM
Project Account Code DM7300
7981 Hadia Almahli 11/5/17

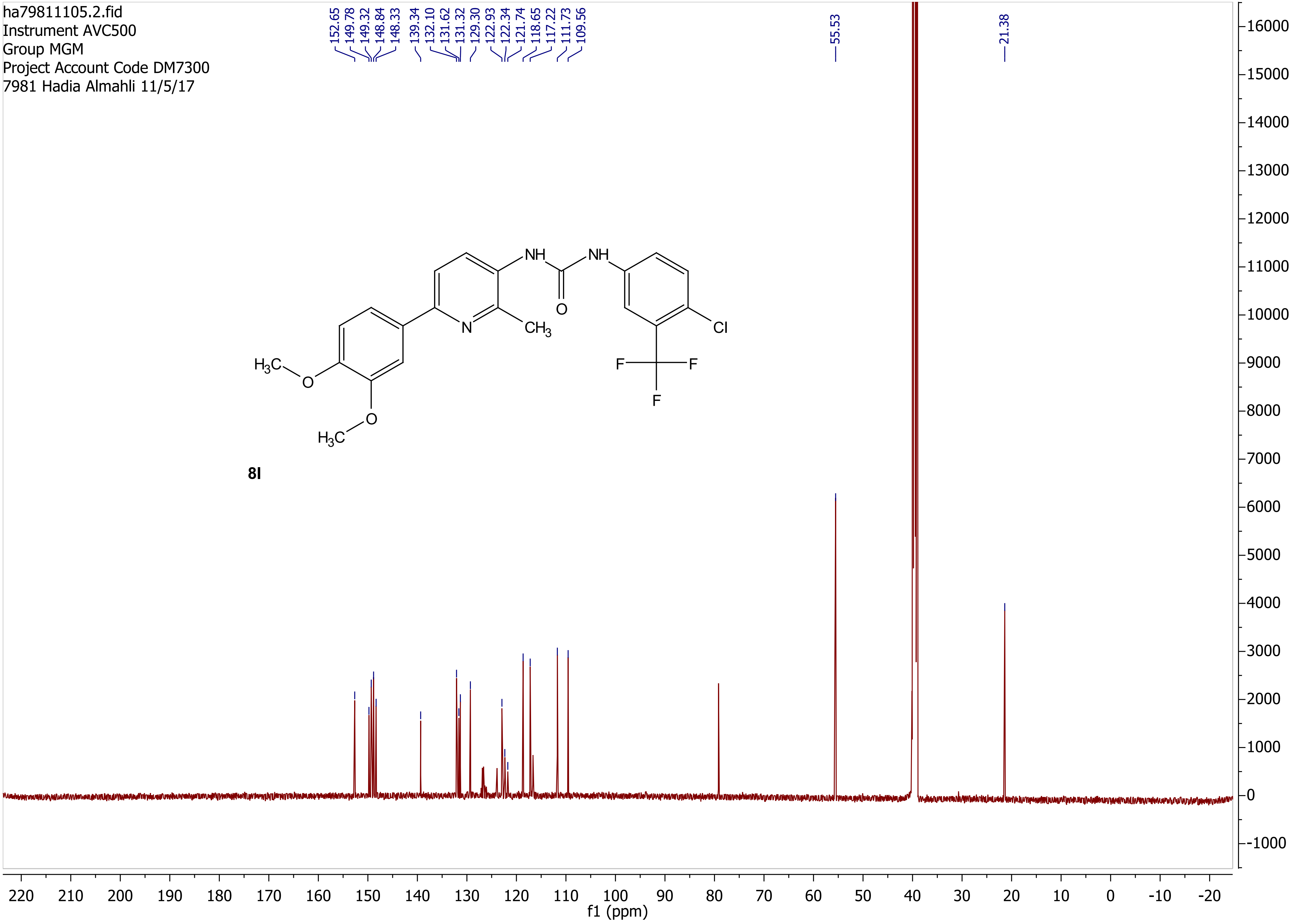

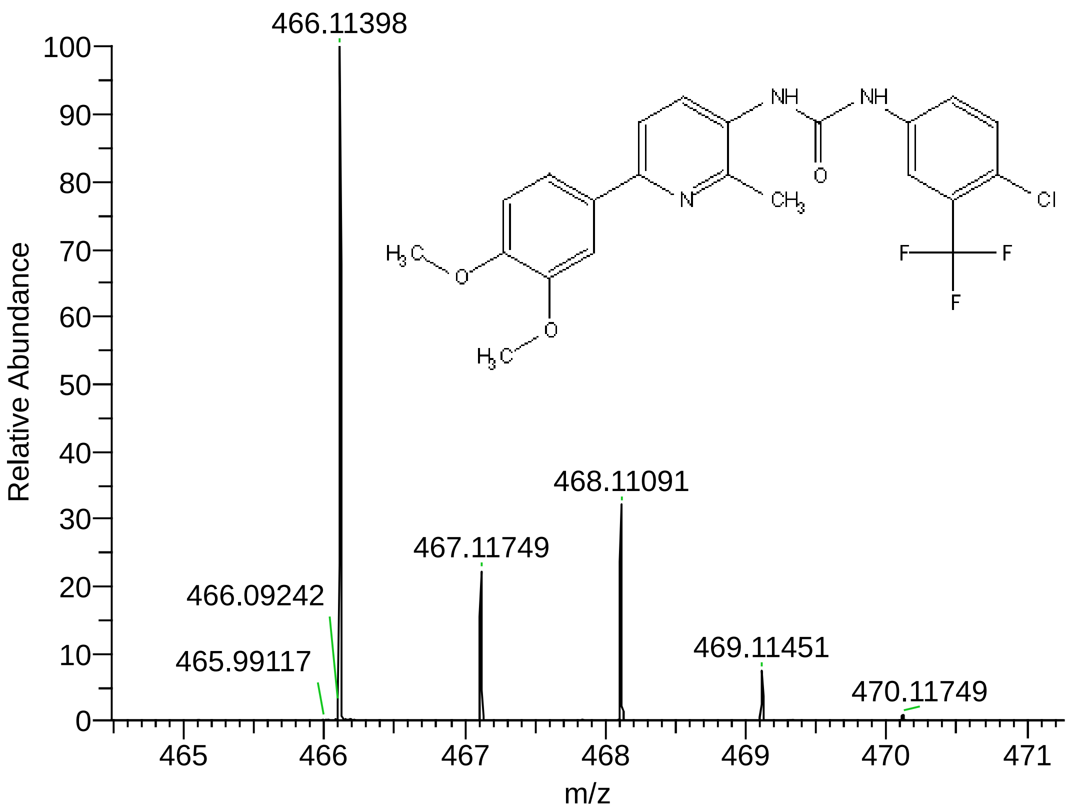

NL: 3.61E6
ESI64331 #15-25 RT: 0.17-0.28 AV: 6 NL: 2.93E+007
T: FTMS {1,1} + p ESI Full ms [80.00-1600.00]

Measured Spectrum

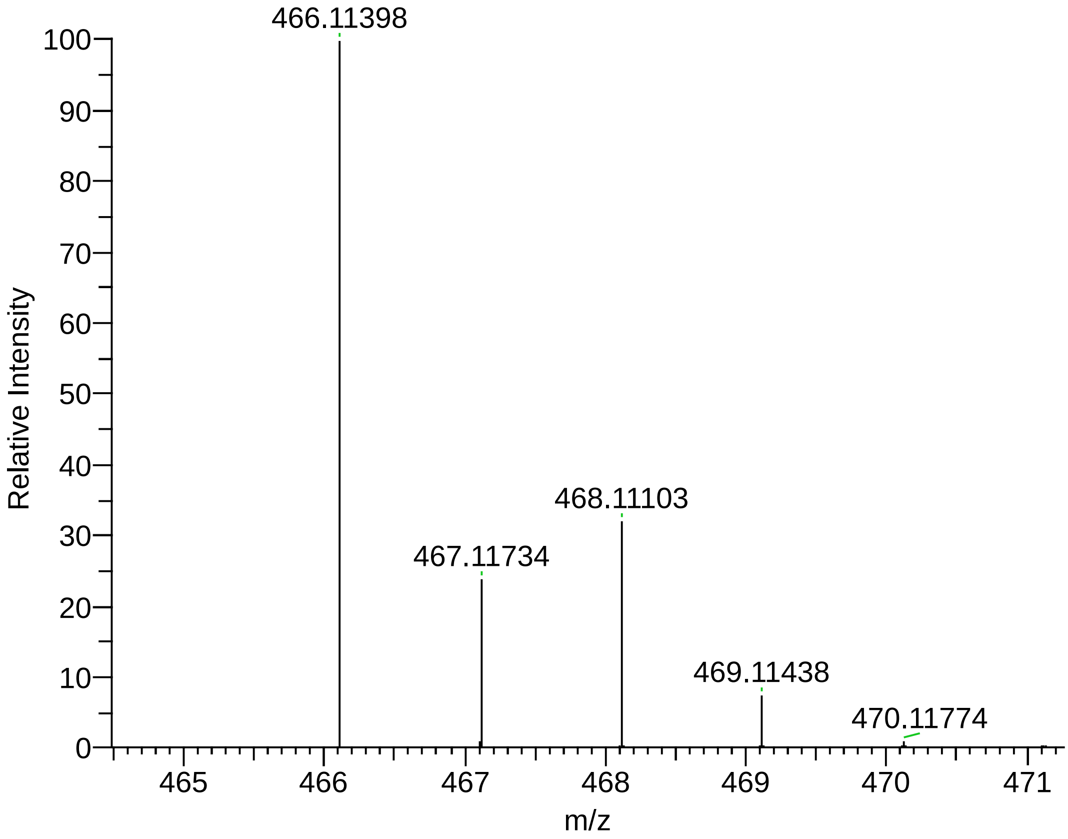

NL: 5.86E5
C22H20O3N3Cl1F3: C_{22} H_{20} O_3 N_3 Cl F_3
Chrg 1 R: 1000000 Res. Pwr. @FWHM

Theoretical Spectrum

m/z	Formula	RDB	Delta ppm	Theo. Mass
466.11398	$C_{22}H_{20}O_3N_3{}^{35}ClF_3$	12.5	0.01	466.11398

Developmental Therapeutics Program<br>One Dose Mean Graph	NSC: D-793907 / 1	Conc: 1.00E-5 Molar	Test Date: Nov 14, 2016
	Experiment ID: 1611OS15		Report Date: Dec 01, 2016

Panel/Cell Line	Growth Percent
**Leukemia**	
CCRF-CEM	42.94
HL-60(TB)	49.75
K-562	32.55
MOLT-4	33.74
RPMI-8226	22.58
SR	36.30
**Non-Small Cell Lung Cancer**	
A549/ATCC	42.47
EKVX	41.42
HOP-62	67.99
HOP-92	68.79
NCI-H226	58.09
NCI-H23	47.63
NCI-H322M	64.44
NCI-H460	48.67
NCI-H522	28.48
**Colon Cancer**	
COLO 205	84.52
HCC-2998	81.09
HCT-116	26.93
HCT-15	40.87
HT29	47.65
KM12	45.61
SW-620	81.01
**CNS Cancer**	
SF-268	74.51
SF-295	47.56
SF-539	80.84
SNB-19	67.81
SNB-75	77.64
U251	50.16
**Melanoma**	
LOX IMVI	47.72
MALME-3M	90.58
M14	37.25
MDA-MB-435	69.59
SK-MEL-2	75.95
SK-MEL-28	69.29
SK-MEL-5	41.63
UACC-257	61.43
UACC-62	63.91
**Ovarian Cancer**	
IGROV1	91.33
OVCAR-3	41.93
OVCAR-4	42.94
OVCAR-5	83.05
OVCAR-8	61.89
NCI/ADR-RES	53.15
SK-OV-3	82.08
**Renal Cancer**	
786-0	77.34
A498	52.08
RXF 393	85.91
SN12C	67.99
TK-10	76.66
UO-31	55.08
**Prostate Cancer**	
PC-3	24.40
DU-145	68.79
**Breast Cancer**	
MCF7	41.47
MDA-MB-231/ATCC	68.55
HS 578T	88.85
BT-549	66.40
T-47D	26.51
MDA-MB-468	27.53
Mean	57.47
Delta	34.89
Range	68.75

Mean Growth Percent - Growth Percent

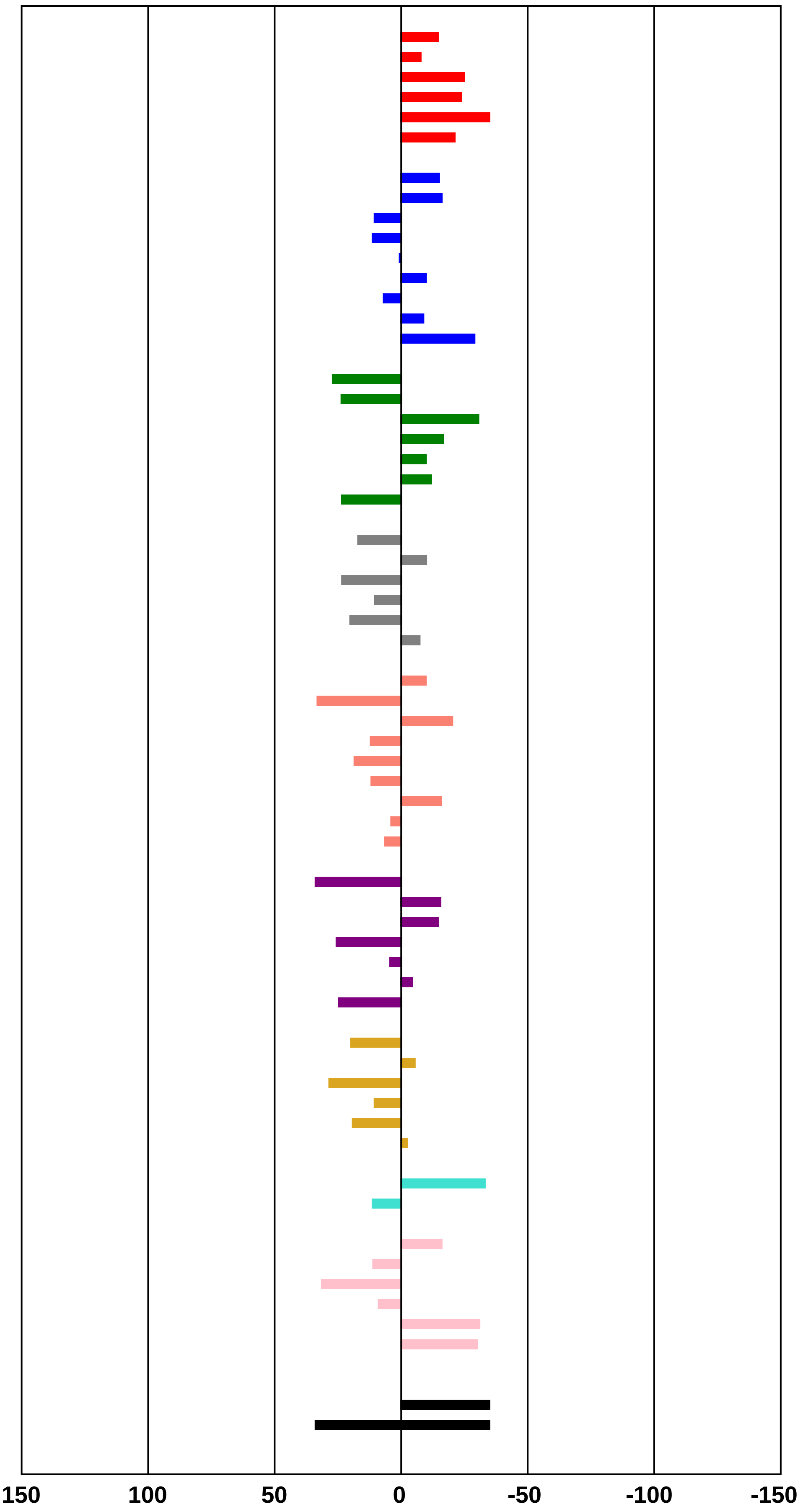

Developmental Therapeutics Program

One Dose Mean Graph

NSC: D-793908 / 1 | **Conc:** 1.00E-5 Molar | **Test Date:** Nov 14, 2016

Experiment ID: 1611OS15 | **Report Date:** Dec 01, 2016

Panel/Cell Line	Growth Percent
Leukemia	
CCRF-CEM	81.43
HL-60(TB)	79.74
K-562	84.35
MOLT-4	72.42
RPMI-8226	90.15
SR	43.88
Non-Small Cell Lung Cancer	
A549/ATCC	68.26
EKVX	92.90
HOP-62	81.25
HOP-92	81.02
NCI-H226	97.81
NCI-H23	94.08
NCI-H322M	65.27
NCI-H460	64.55
NCI-H522	67.47
Colon Cancer	
COLO 205	102.39
HCC-2998	104.24
HCT-116	76.10
HCT-15	85.74
HT29	85.13
KM12	93.09
SW-620	77.06
CNS Cancer	
SF-268	96.43
SF-295	97.10
SF-539	84.16
SNB-19	94.20
SNB-75	84.87
U251	76.51
Melanoma	
LOX IMVI	85.30
MALME-3M	104.47
M14	86.55
MDA-MB-435	88.00
SK-MEL-2	104.03
SK-MEL-28	89.84
SK-MEL-5	87.05
UACC-257	78.79
UACC-62	71.19
Ovarian Cancer	
IGROV1	83.85
OVCAR-3	101.66
OVCAR-4	74.67
OVCAR-5	93.01
OVCAR-8	93.26
NCI/ADR-RES	92.03
SK-OV-3	82.66
Renal Cancer	
786-0	90.10
A498	79.24
RXF 393	98.51
SN12C	98.33
TK-10	102.41
UO-31	89.14
Prostate Cancer	
PC-3	76.01
DU-145	98.63
Breast Cancer	
MCF7	74.74
MDA-MB-231/ATCC	97.34
HS 578T	98.36
BT-549	96.71
T-47D	72.90
MDA-MB-468	91.54
Mean	86.24
Delta	42.36
Range	60.59

Mean Growth Percent - Growth Percent

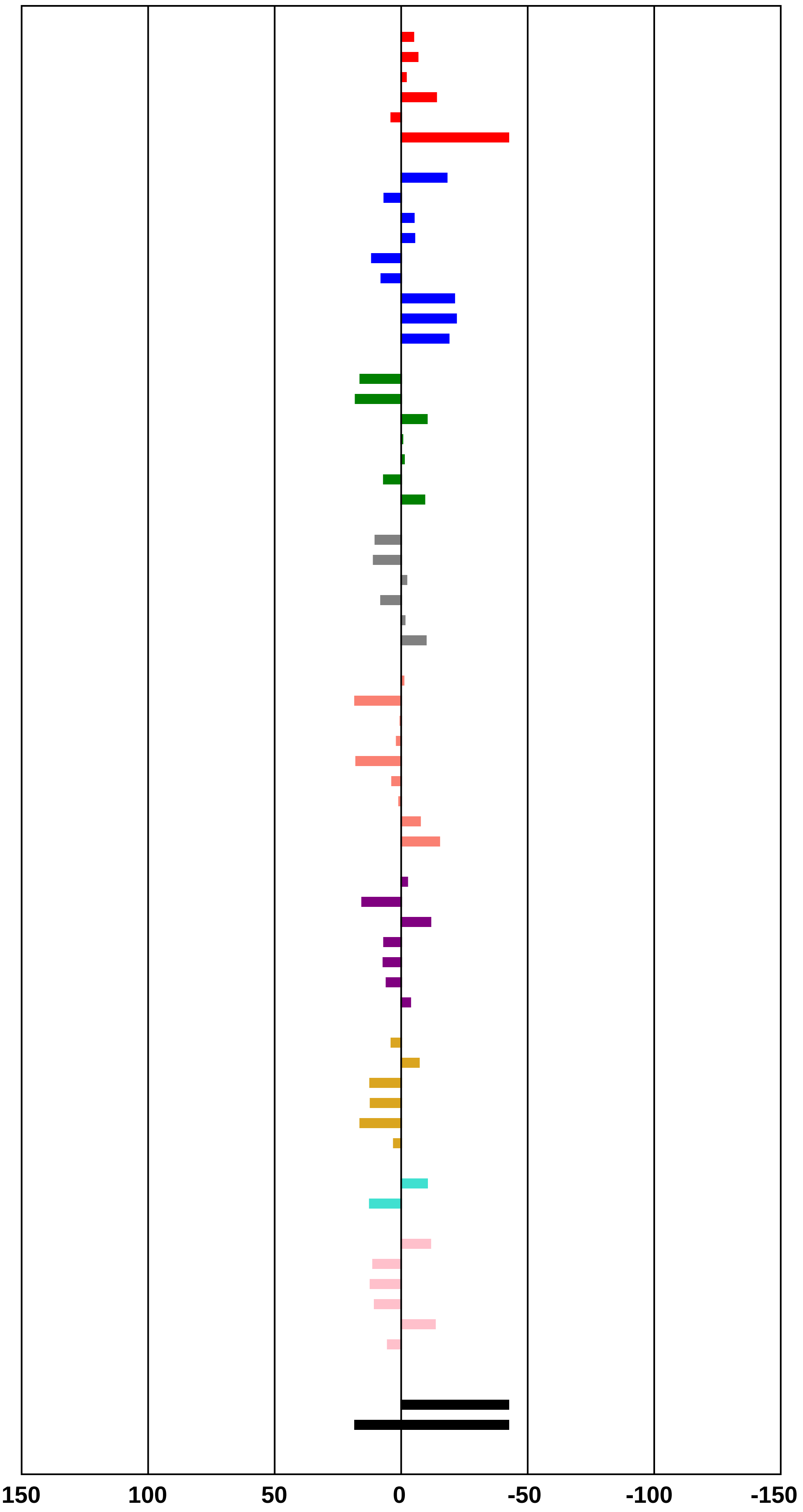

Developmental Therapeutics Program	NSC: D-793925 / 1	Conc: 1.00E-5 Molar	Test Date: Nov 14, 2016
One Dose Mean Graph	Experiment ID: 1611OS15		Report Date: Dec 01, 2016

Panel/Cell Line	Growth Percent
Leukemia	
CCRF-CEM	29.88
HL-60(TB)	32.27
K-562	16.70
MOLT-4	24.60
RPMI-8226	9.87
SR	35.68
Non-Small Cell Lung Cancer	
A549/ATCC	38.71
EKVX	49.19
HOP-62	62.10
HOP-92	27.03
NCI-H226	65.24
NCI-H23	30.94
NCI-H322M	71.44
NCI-H460	30.90
NCI-H522	24.81
Colon Cancer	
COLO 205	80.09
HCC-2998	69.17
HCT-116	23.73
HCT-15	37.80
HT29	46.44
KM12	37.36
SW-620	67.04
CNS Cancer	
SF-268	73.73
SF-295	46.97
SF-539	81.32
SNB-19	57.33
SNB-75	73.44
U251	33.63
Melanoma	
LOX IMVI	64.51
MALME-3M	82.56
M14	31.87
MDA-MB-435	71.95
SK-MEL-2	62.13
SK-MEL-28	71.43
SK-MEL-5	55.66
UACC-257	57.07
UACC-62	65.57
Ovarian Cancer	
IGROV1	52.86
OVCAR-3	27.65
OVCAR-4	45.11
OVCAR-5	89.11
OVCAR-8	57.40
NCI/ADR-RES	49.78
SK-OV-3	68.11
Renal Cancer	
786-0	56.08
A498	57.74
RXF 393	81.37
SN12C	57.41
TK-10	65.92
UO-31	39.33
Prostate Cancer	
PC-3	14.67
DU-145	54.52
Breast Cancer	
MCF7	36.68
MDA-MB-231/ATCC	85.04
HS 578T	76.98
BT-549	66.19
T-47D	21.49
MDA-MB-468	38.52
Mean	51.42
Delta	41.55
Range	79.24

Mean Growth Percent - Growth Percent

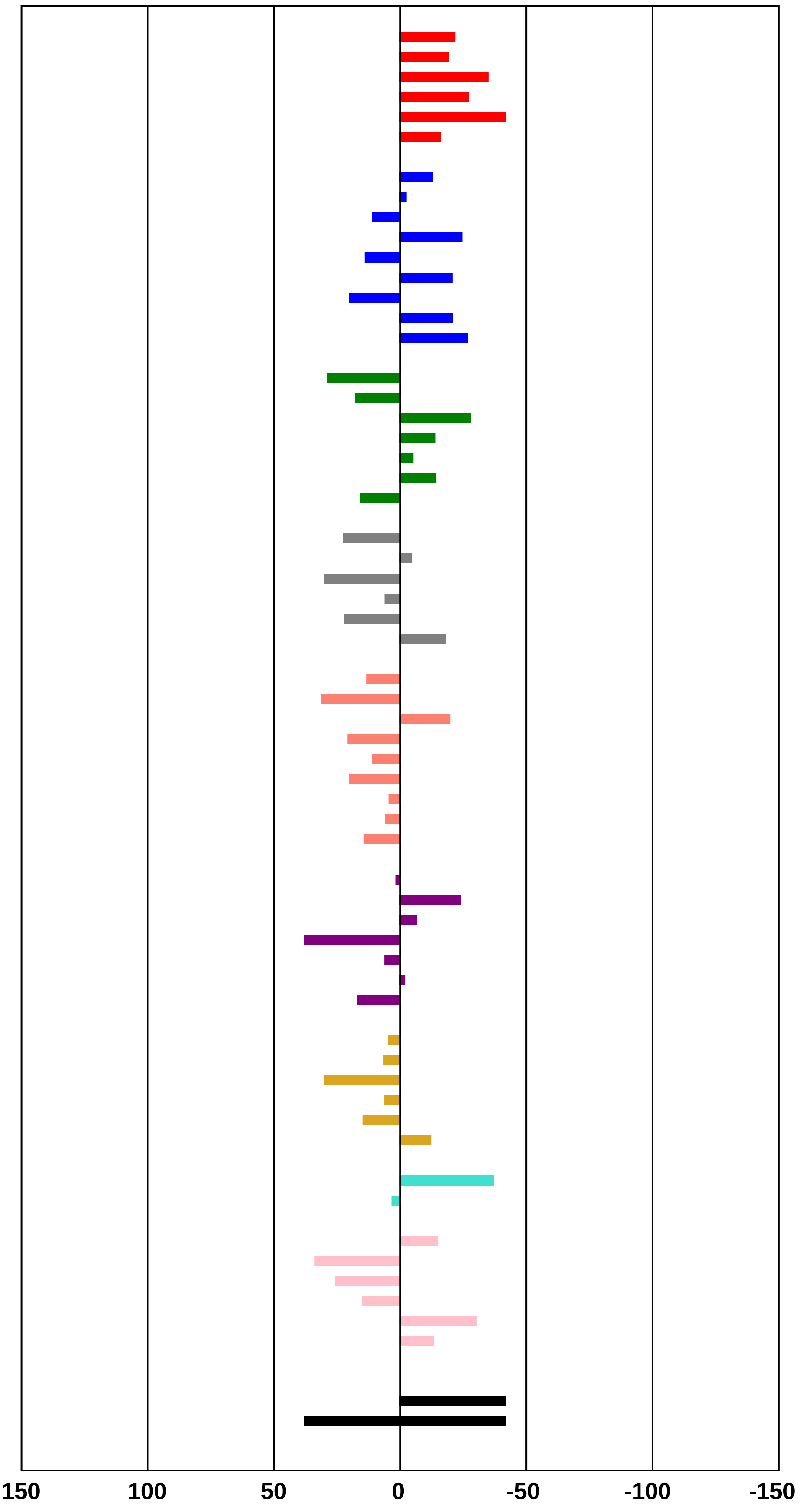

Developmental Therapeutics Program	NSC: D-793919 / 1	Conc: 1.00E-5 Molar	Test Date: Nov 14, 2016
One Dose Mean Graph	Experiment ID: 1611OS15		Report Date: Dec 01, 2016

Panel/Cell Line	Growth Percent
Leukemia	
CCRF-CEM	50.94
HL-60(TB)	61.08
K-562	31.93
MOLT-4	39.58
RPMI-8226	34.61
SR	51.13
Non-Small Cell Lung Cancer	
A549/ATCC	60.26
EKVX	66.90
HOP-62	92.32
HOP-92	55.33
NCI-H226	72.63
NCI-H23	71.94
NCI-H322M	92.59
NCI-H460	61.77
NCI-H522	40.25
Colon Cancer	
COLO 205	88.09
HCC-2998	84.39
HCT-116	17.63
HCT-15	48.77
HT29	59.96
KM12	66.00
SW-620	91.19
CNS Cancer	
SF-268	75.96
SF-295	65.28
SF-539	77.24
SNB-19	78.84
SNB-75	66.71
U251	63.88
Melanoma	
LOX IMVI	70.96
MALME-3M	95.26
M14	31.21
MDA-MB-435	73.17
SK-MEL-2	73.78
SK-MEL-28	75.52
SK-MEL-5	58.49
UACC-257	81.62
UACC-62	81.66
Ovarian Cancer	
IGROV1	91.46
OVCAR-3	69.16
OVCAR-4	59.07
OVCAR-5	94.03
OVCAR-8	81.32
NCI/ADR-RES	66.87
SK-OV-3	101.37
Renal Cancer	
786-0	69.08
A498	61.34
RXF 393	77.55
SN12C	79.32
TK-10	79.75
UO-31	74.95
Prostate Cancer	
PC-3	27.45
DU-145	82.57
Breast Cancer	
MCF7	62.46
MDA-MB-231/ATCC	69.50
HS 578T	74.54
BT-549	71.52
T-47D	53.57
MDA-MB-468	49.56
Mean	67.33
Delta	49.70
Range	83.74

Mean Growth Percent - Growth Percent

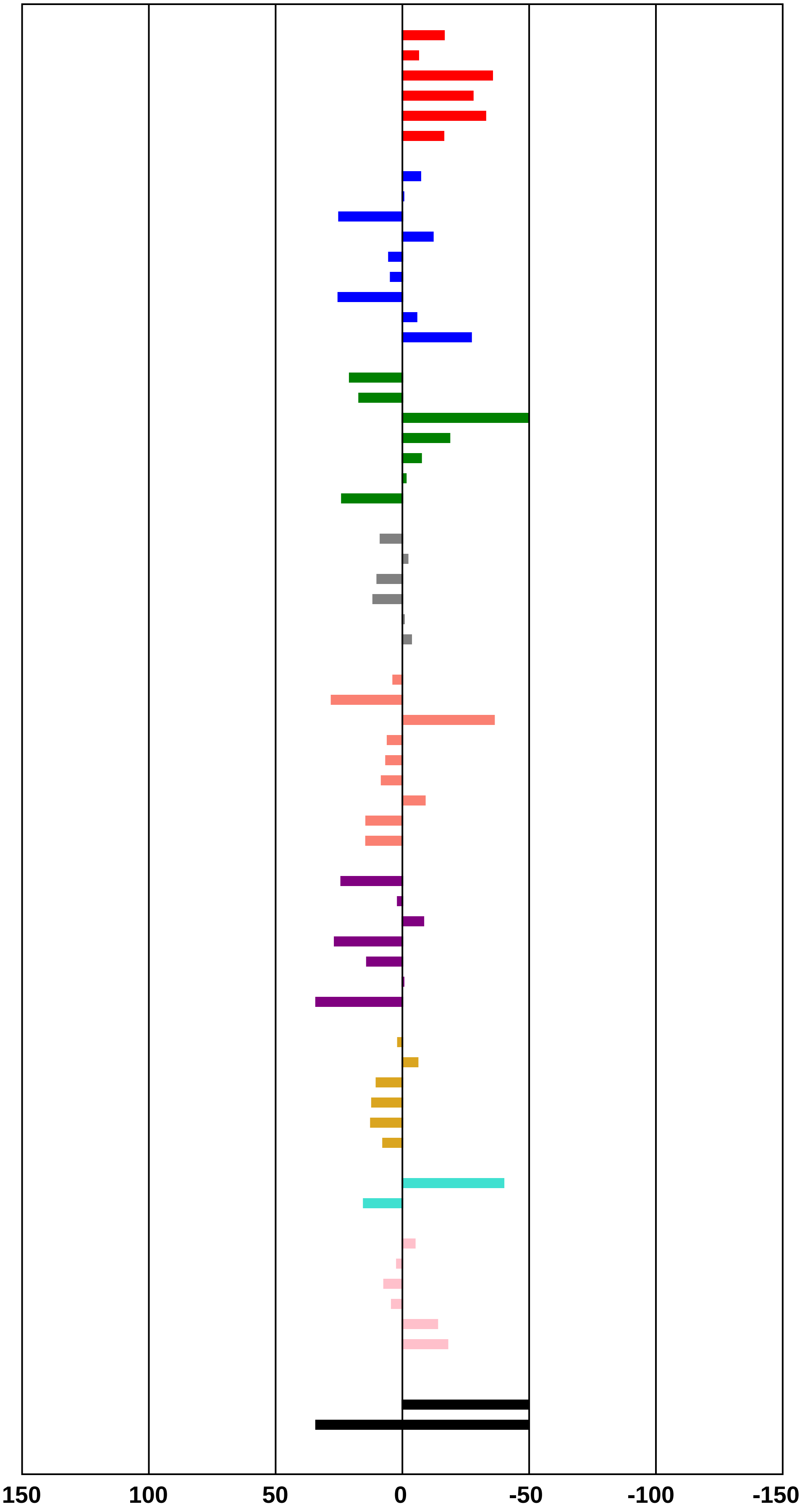

Developmental Therapeutics Program	NSC: D-793924 / 1	Conc: 1.00E-5 Molar	Test Date: Nov 14, 2016
One Dose Mean Graph	Experiment ID: 1611OS15		Report Date: Dec 01, 2016

Panel/Cell Line	Growth Percent
Leukemia	
CCRF-CEM	68.29
HL-60(TB)	63.92
K-562	40.86
MOLT-4	42.64
RPMI-8226	51.32
SR	52.07
Non-Small Cell Lung Cancer	
A549/ATCC	61.64
EKVX	83.81
HOP-62	77.77
HOP-92	60.18
NCI-H226	91.65
NCI-H23	89.49
NCI-H322M	85.92
NCI-H460	83.94
NCI-H522	59.88
Colon Cancer	
COLO 205	93.06
HCC-2998	88.09
HCT-116	63.31
HCT-15	60.62
HT29	74.98
KM12	78.93
SW-620	98.24
CNS Cancer	
SF-268	87.00
SF-295	83.73
SF-539	89.70
SNB-19	97.00
SNB-75	84.12
U251	83.31
Melanoma	
LOX IMVI	80.89
MALME-3M	98.93
M14	50.80
MDA-MB-435	87.66
SK-MEL-2	84.73
SK-MEL-28	79.52
SK-MEL-5	68.63
UACC-257	71.26
UACC-62	75.01
Ovarian Cancer	
IGROV1	100.28
OVCAR-3	90.37
OVCAR-4	91.75
OVCAR-5	88.21
OVCAR-8	90.16
NCI/ADR-RES	84.81
SK-OV-3	83.62
Renal Cancer	
786-0	71.87
A498	69.84
RXF 393	82.62
SN12C	86.84
TK-10	99.31
UO-31	79.44
Prostate Cancer	
PC-3	39.30
DU-145	101.79
Breast Cancer	
MCF7	75.86
MDA-MB-231/ATCC	87.05
HS 578T	95.46
BT-549	91.71
T-47D	49.90
MDA-MB-468	71.36
Mean	78.01
Delta	38.71
Range	62.49

Mean Growth Percent - Growth Percent

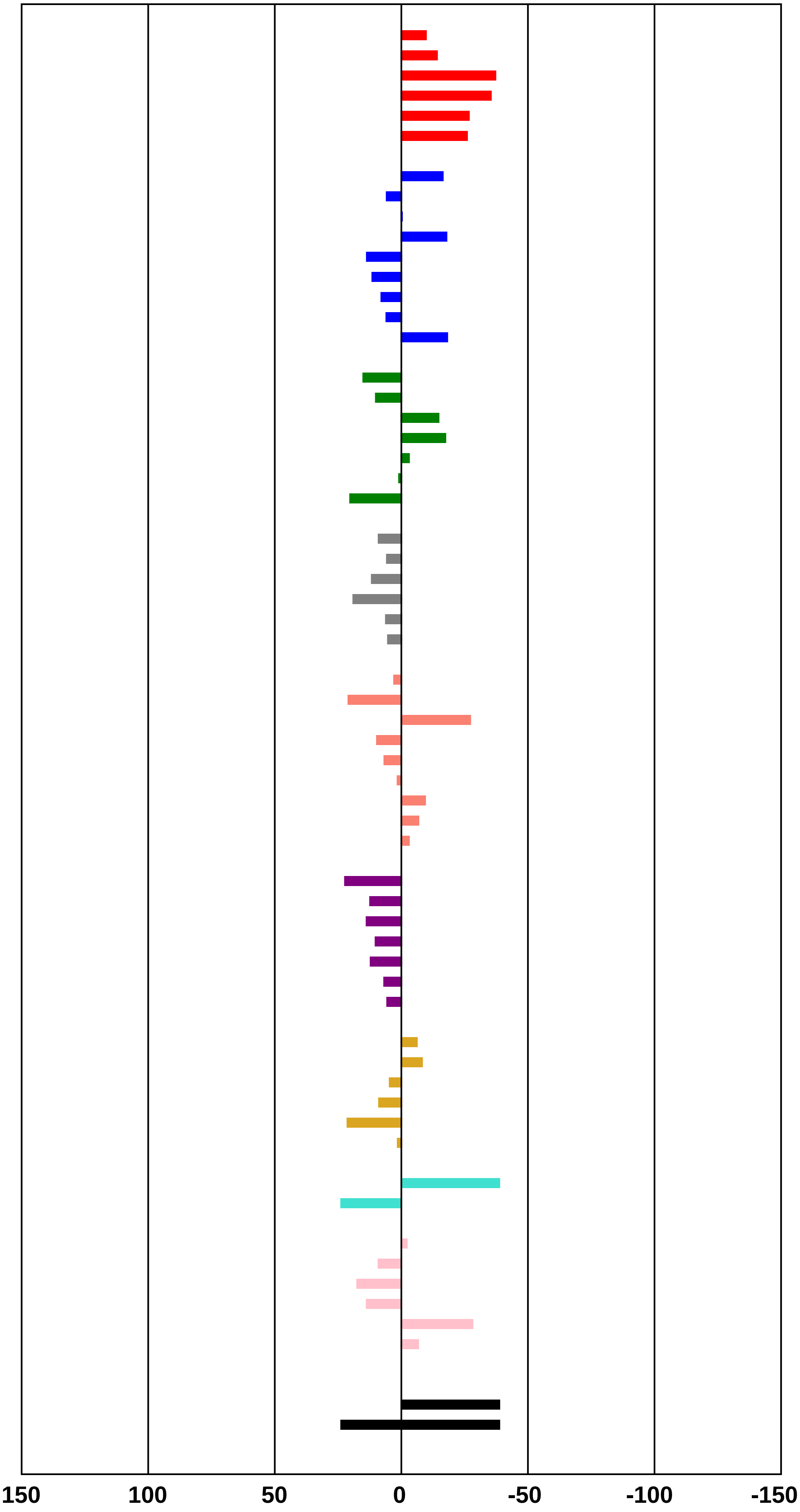

Developmental Therapeutics Program

One Dose Mean Graph

**NSC:** D-793924 / 1	**Conc:** 1.00E-5 Molar
**Test Date:** Nov 14, 2016	**Experiment ID:** 1611OS15
**Report Date:** Dec 01, 2016	

Panel/Cell Line	Growth Percent
Leukemia	
CCRF-CEM	68.29
HL-60(TB)	63.92
K-562	40.86
MOLT-4	42.64
RPMI-8226	51.32
SR	52.07
Non-Small Cell Lung Cancer	
A549/ATCC	61.64
EKVX	83.81
HOP-62	77.77
HOP-92	60.18
NCI-H226	91.65
NCI-H23	89.49
NCI-H322M	85.92
NCI-H460	83.94
NCI-H522	59.88
Colon Cancer	
COLO 205	93.06
HCC-2998	88.09
HCT-116	63.31
HCT-15	60.62
HT29	74.98
KM12	78.93
SW-620	98.24
CNS Cancer	
SF-268	87.00
SF-295	83.73
SF-539	89.70
SNB-19	97.00
SNB-75	84.12
U251	83.31
Melanoma	
LOX IMVI	80.89
MALME-3M	98.93
M14	50.80
MDA-MB-435	87.66
SK-MEL-2	84.73
SK-MEL-28	79.52
SK-MEL-5	68.63
UACC-257	71.26
UACC-62	75.01
Ovarian Cancer	
IGROV1	100.28
OVCAR-3	90.37
OVCAR-4	91.75
OVCAR-5	88.21
OVCAR-8	90.16
NCI/ADR-RES	84.81
SK-OV-3	83.62
Renal Cancer	
786-0	71.87
A498	69.84
RXF 393	82.62
SN12C	86.84
TK-10	99.31
UO-31	79.44
Prostate Cancer	
PC-3	39.30
DU-145	101.79
Breast Cancer	
MCF7	75.86
MDA-MB-231/ATCC	87.05
HS 578T	95.46
BT-549	91.71
T-47D	49.90
MDA-MB-468	71.36
Mean	78.01
Delta	38.71
Range	62.49

Mean Growth Percent - Growth Percent

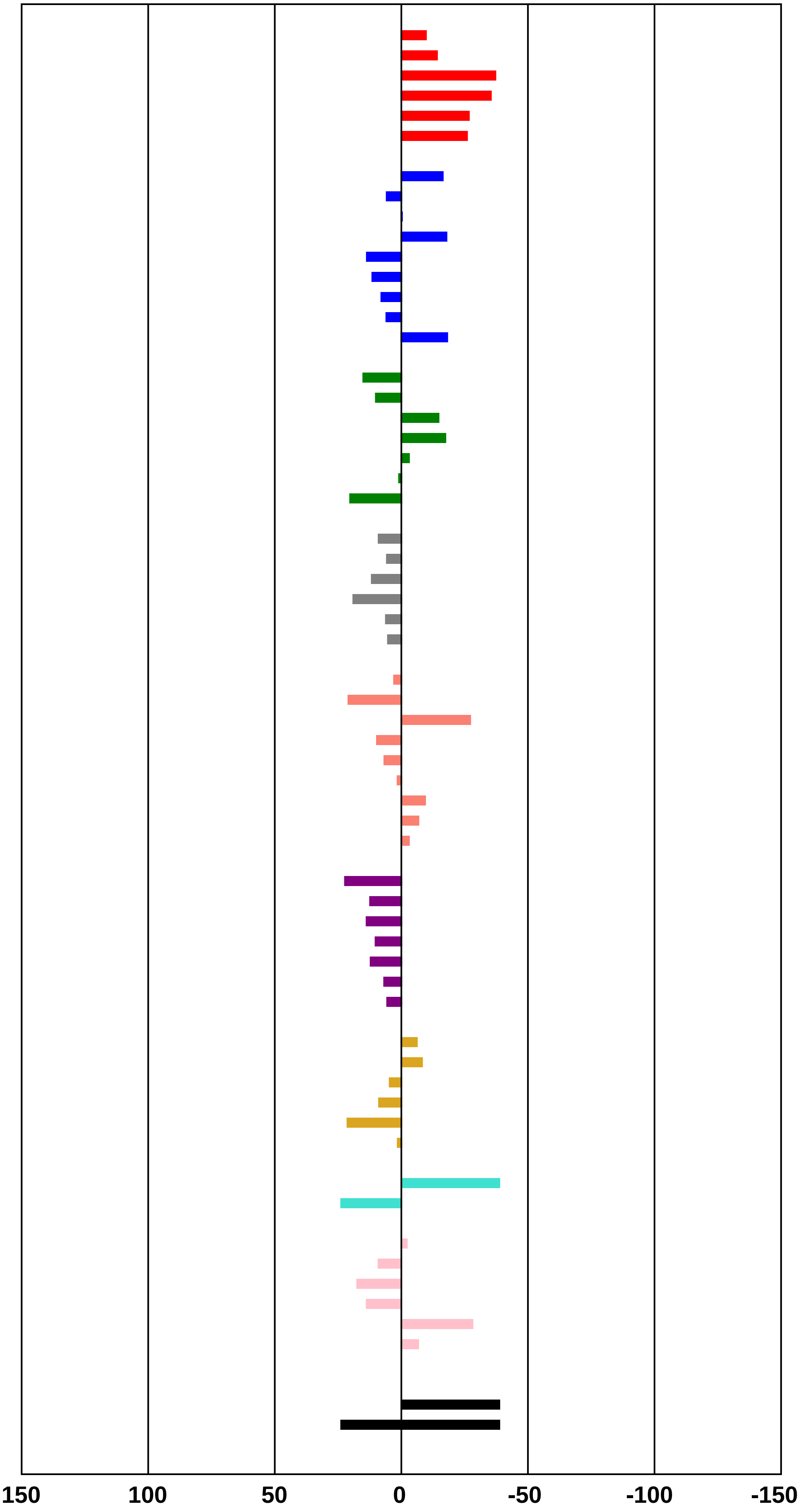

Developmental Therapeutics Program

One Dose Mean Graph

**NSC:** D-793915 / 1	**Conc:** 1.00E-5 Molar	**Test Date:** Nov 14, 2016
**Experiment ID:** 1611OS15		**Report Date:** Dec 01, 2016

Panel/Cell Line	Growth Percent
Leukemia	
CCRF-CEM	55.04
HL-60(TB)	52.58
K-562	49.47
MOLT-4	43.68
RPMI-8226	43.88
SR	60.09
Non-Small Cell Lung Cancer	
A549/ATCC	44.62
EKVX	67.30
HOP-62	77.10
HOP-92	70.53
NCI-H226	72.73
NCI-H23	71.36
NCI-H322M	87.05
NCI-H460	52.19
NCI-H522	43.02
Colon Cancer	
COLO 205	93.52
HCC-2998	93.63
HCT-116	45.30
HCT-15	49.62
HT29	56.20
KM12	70.44
SW-620	90.92
CNS Cancer	
SF-268	75.77
SF-295	71.54
SF-539	87.81
SNB-19	78.75
SNB-75	80.45
U251	55.26
Melanoma	
LOX IMVI	78.57
MALME-3M	90.89
M14	47.02
MDA-MB-435	77.84
SK-MEL-2	87.87
SK-MEL-28	73.58
SK-MEL-5	63.85
UACC-257	56.26
UACC-62	77.31
Ovarian Cancer	
IGROV1	83.06
OVCAR-3	57.00
OVCAR-4	75.13
OVCAR-5	85.15
OVCAR-8	81.32
NCI/ADR-RES	69.67
SK-OV-3	84.22
Renal Cancer	
786-0	75.10
A498	57.69
RXF 393	72.65
SN12C	78.52
TK-10	88.19
UO-31	72.37
Prostate Cancer	
PC-3	33.54
DU-145	80.60
Breast Cancer	
MCF7	58.28
MDA-MB-231/ATCC	88.41
HS 578T	81.01
BT-549	88.97
T-47D	49.20
MDA-MB-468	48.57
Mean	68.99
Delta	35.45
Range	60.09

Mean Growth Percent - Growth Percent

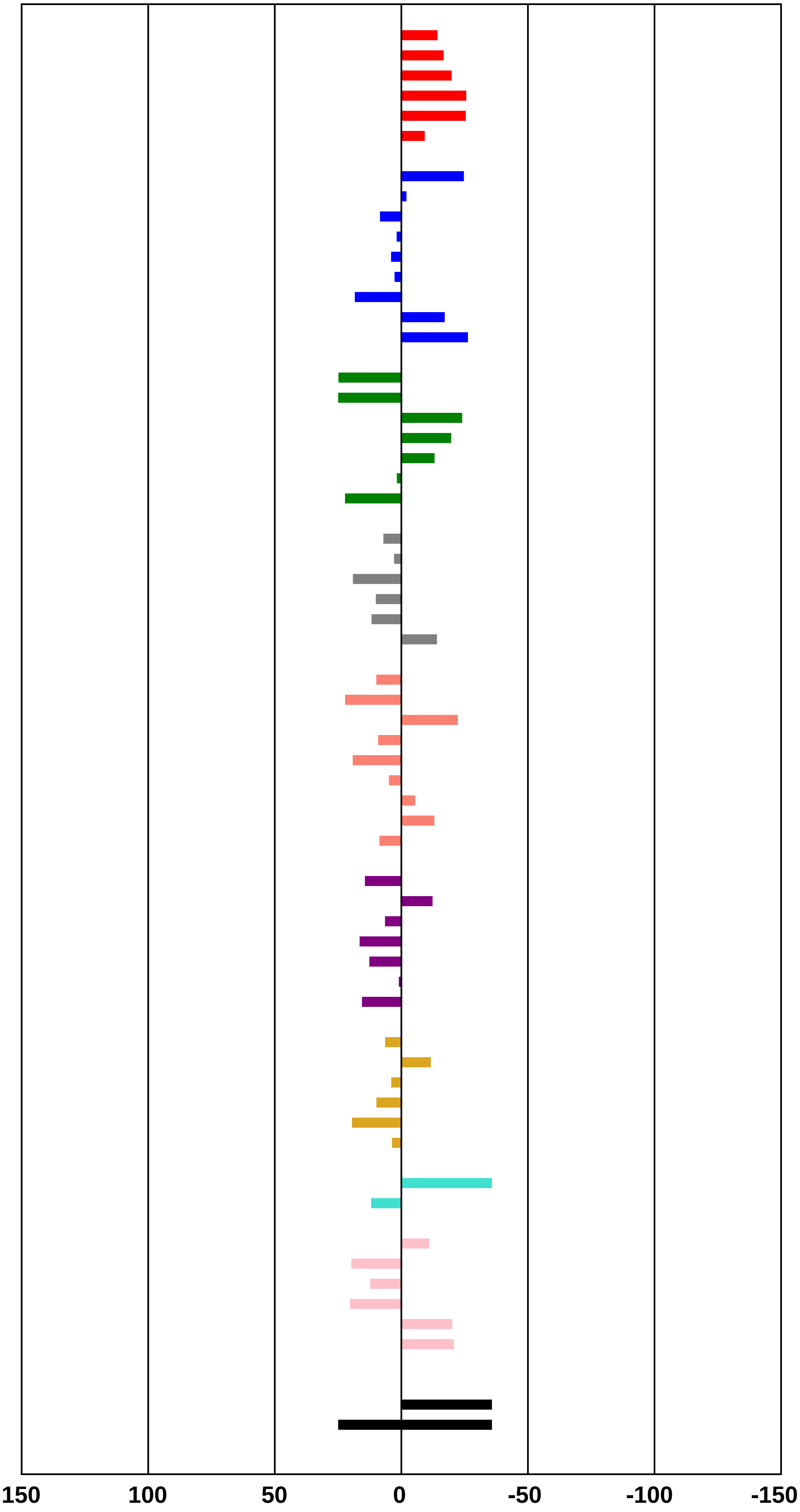

Developmental Therapeutics Program	NSC: D-793929 / 1	Conc: 1.00E-5 Molar	Test Date: Nov 14, 2016
One Dose Mean Graph	Experiment ID: 1611OS15		Report Date: Dec 01, 2016

Panel/Cell Line	Growth Percent
Leukemia	
CCRF-CEM	89.11
HL-60(TB)	73.33
K-562	78.49
MOLT-4	65.34
RPMI-8226	74.18
SR	82.12
Non-Small Cell Lung Cancer	
A549/ATCC	90.54
EKVX	100.68
HOP-62	94.75
HOP-92	81.00
NCI-H226	94.44
NCI-H23	90.97
NCI-H322M	95.22
NCI-H460	102.12
NCI-H522	53.07
Colon Cancer	
COLO 205	119.56
HCC-2998	97.15
HCT-116	78.84
HCT-15	91.11
HT29	75.95
KM12	96.43
SW-620	101.68
CNS Cancer	
SF-268	94.56
SF-295	99.68
SF-539	93.97
SNB-19	115.50
SNB-75	104.39
U251	94.15
Melanoma	
LOX IMVI	92.56
MALME-3M	92.61
M14	90.93
MDA-MB-435	100.45
SK-MEL-2	87.95
SK-MEL-28	99.69
SK-MEL-5	84.32
UACC-257	97.19
UACC-62	93.89
Ovarian Cancer	
IGROV1	112.08
OVCAR-3	101.69
OVCAR-4	126.33
OVCAR-5	104.54
OVCAR-8	102.37
NCI/ADR-RES	92.25
SK-OV-3	99.49
Renal Cancer	
786-0	92.27
A498	85.18
RXF 393	108.45
SN12C	93.61
TK-10	96.16
UO-31	80.45
Prostate Cancer	
PC-3	59.08
DU-145	99.53
Breast Cancer	
MCF7	90.01
MDA-MB-231/ATCC	106.37
HS 578T	99.31
BT-549	87.21
T-47D	81.11
MDA-MB-468	99.00
Mean	92.83
Delta	39.76
Range	73.26

Mean Growth Percent - Growth Percent

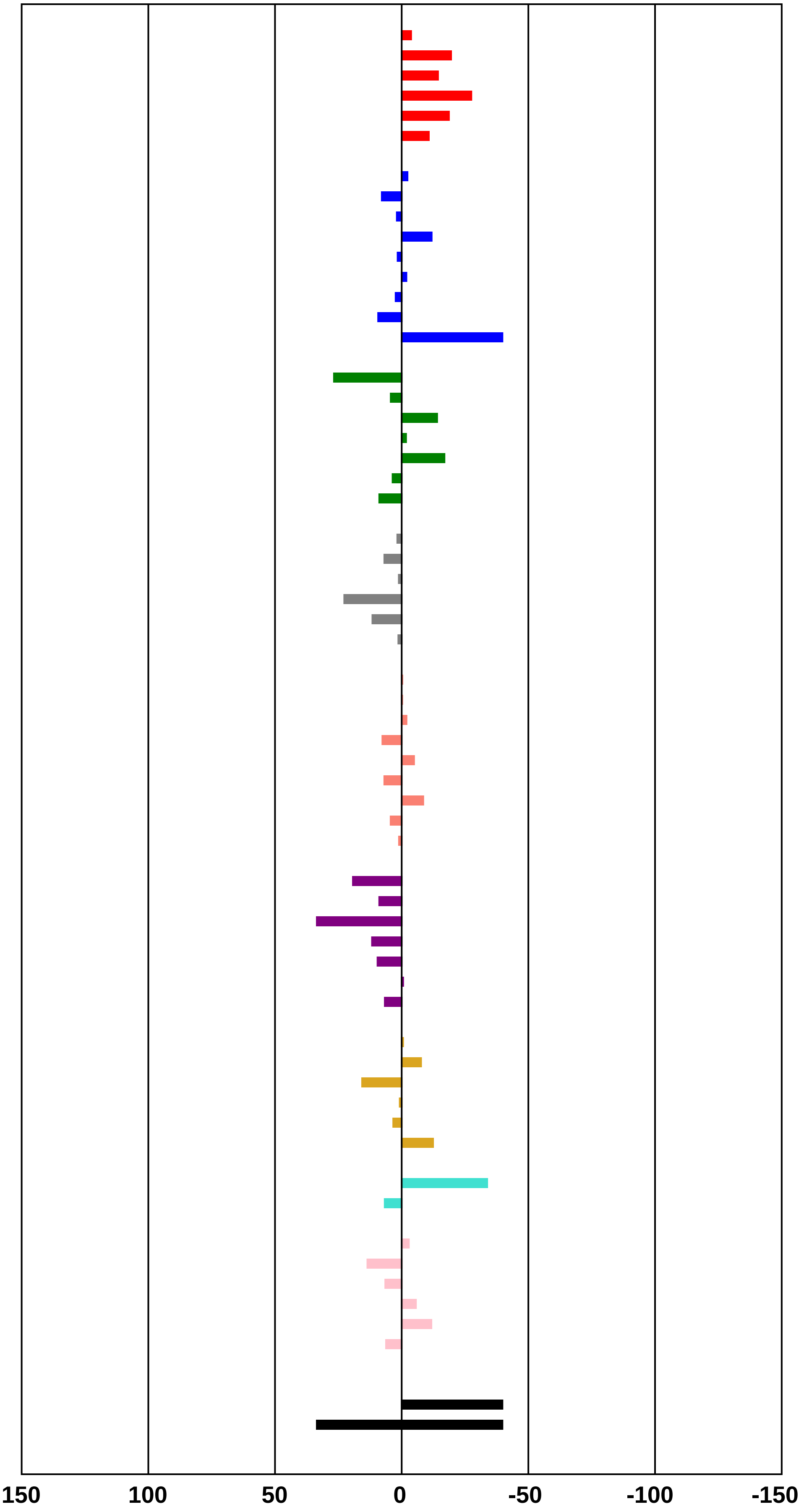

Developmental Therapeutics Program	NSC: D-793926 / 1	Conc: 1.00E-5 Molar	Test Date: Nov 14, 2016
One Dose Mean Graph	Experiment ID: 1611OS15		Report Date: Dec 01, 2016

Panel/Cell Line	Growth Percent
Leukemia	
CCRF-CEM	97.16
HL-60(TB)	85.74
K-562	73.25
MOLT-4	85.62
RPMI-8226	83.77
SR	57.29
Non-Small Cell Lung Cancer	
A549/ATCC	57.02
EKVX	91.15
HOP-62	56.53
HOP-92	73.37
NCI-H226	58.04
NCI-H23	67.43
NCI-H322M	86.96
NCI-H460	17.58
NCI-H522	49.88
Colon Cancer	
COLO 205	91.95
HCC-2998	83.10
HCT-116	49.40
HCT-15	91.77
HT29	68.76
KM12	45.29
SW-620	64.84
CNS Cancer	
SF-268	59.25
SF-295	101.75
SF-539	70.63
SNB-19	71.47
SNB-75	47.52
U251	22.08
Melanoma	
LOX IMVI	88.83
MALME-3M	44.12
M14	72.14
MDA-MB-435	65.17
SK-MEL-2	79.55
SK-MEL-28	86.76
SK-MEL-5	22.98
UACC-257	55.47
UACC-62	59.87
Ovarian Cancer	
IGROV1	77.14
OVCAR-3	102.08
OVCAR-4	29.55
OVCAR-5	98.04
OVCAR-8	47.38
NCI/ADR-RES	84.96
SK-OV-3	74.76
Renal Cancer	
786-0	11.07
A498	71.94
RXF 393	58.20
SN12C	64.38
TK-10	60.42
UO-31	81.72
Prostate Cancer	
PC-3	81.42
DU-145	72.24
Breast Cancer	
MCF7	65.14
MDA-MB-231/ATCC	40.48
HS 578T	39.96
BT-549	50.44
T-47D	60.36
MDA-MB-468	83.00
Mean	66.18
Delta	55.11
Range	91.01

Mean Growth Percent - Growth Percent

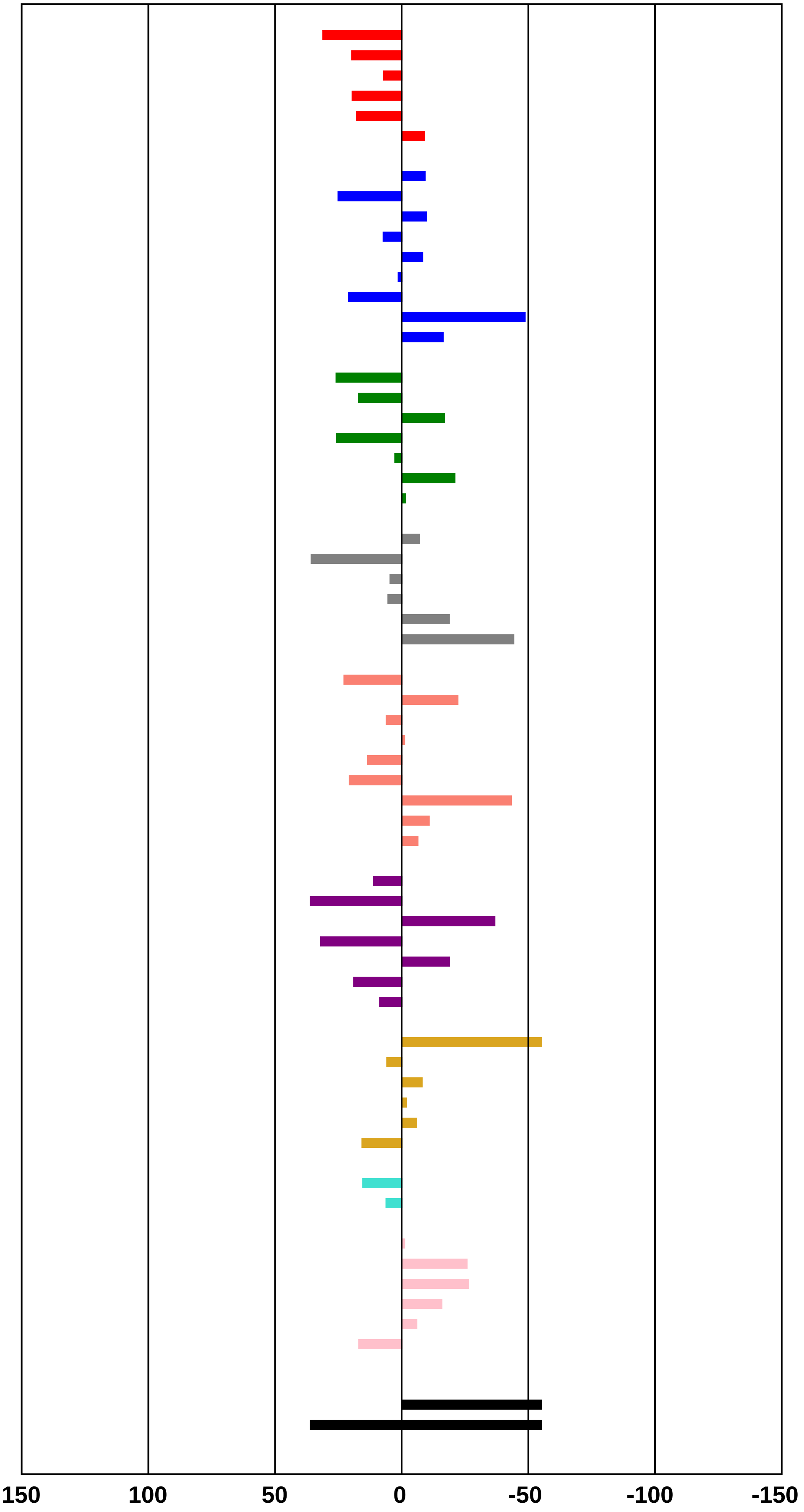

Supplement: Supplementary file 1 [file molecules-23-01459-s001.pdf]
